# Rigidifying Qubit Candidates in a Cu‐Porphyrin Nanohoop: Dipolar Coupling in Spin Pairs and Spin‐Polarized Ground State

**DOI:** 10.1002/anie.202522950

**Published:** 2025-12-09

**Authors:** Xingmao Chang, Ashley J. Redman, Linda Zedler, Louis Blechschmidt, Adriana Sacristán‐Martín, Fabian Schwer, Inhar Imaz, Markus P. B. Wiedmaier, Xavi Ribas, Daniel Maspoch, Benjamin Dietzek‐Ivanšić, Sabine Richert, Max von Delius

**Affiliations:** ^1^ Institute of Organic Chemistry and Center for Integrated Quantum Science and Technology Ulm University Albert‐Einstein‐Allee 11 89081 Ulm Germany; ^2^ Institute of Physical Chemistry II Ulm University Albert‐Einstein‐Allee 47 Ulm 89081 Germany; ^3^ Leibniz Institute of Photonic Technology Albert‐Einstein‐Straße 9 Jena 07745 Germany; ^4^ Institute for Physical Chemistry Friedrich Schiller University Jena Helmholtzweg 4 07743 Jena Germany; ^5^ Catalan Institute of Nanoscience and Nanotechnology (ICN2) CSIC and The Barcelona Institute of Science and Technology Bellaterra Catalonia 08193 Spain; ^6^ ICREA Pg. Lluís Companys 23 Barcelona 08010 Spain; ^7^ Institut de Química Computacional i Catàlisi and Departament de Química Universitat de Girona Catalonia E‐17003 Spain

**Keywords:** Copper porphyrins, Electron paramagnetic resonance spectroscopy, Molecular quantum bits, Rigid macrocycles, Transient absorption spectroscopy

## Abstract

Assembling molecular qubit candidates with precise control over the position and orientation of spin centers is an important contemporary challenge for synthesis. In this work, we show that the rigidity of highly strained macrocycles from the cycloparaphenylene family gives rise to distinct spin‐spin and light‐spin interactions that make such few‐qubit systems a promising testing ground for future quantum technologies. We synthesized conjugated nanohoop **Cu[3]CPTA** that comprises three Cu(II)porphyrin centers (*S* = ½) with a Cu–Cu distance of ca. 18 Å (by single‐crystal X‐ray diffraction). Continuous‐wave (cw) and pulse electron‐paramagnetic resonance (EPR) studies revealed that dipolar coupling in spin pairs is so well defined in this nanohoop that the Cu–Cu distance can be determined accurately via double electron–electron resonance (DEER). By transient cwEPR, we observed a rare case of a light‐induced polarization of a doublet ground state in frozen solution. The fact that ground state polarization was significantly less pronounced in a comparable, but more flexible macrocycle will inspire future efforts to better understand and harness this effect.

## Graphical abstract

A conjugated nanohoop, **Cu[3]CPTA**, containing three Cu(II) porphyrin spin centers (*S* = ½) was synthesized. We show that the exceptionally high “nanohoop rigidity” results in well‐defined dipolar coupling (DEER) and a rare light‐induced spin polarization of the doublet ground state in frozen solution (transient cwEPR).

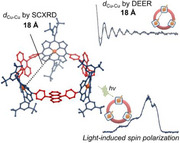

Molecular quantum bit (qubit) candidates promise better tunability and scalability than traditional solid‐state qubit platforms.^[^ ^1^, ^2^ ^]^ Molecular systems, such as paramagnetic (e.g., Cu) porphyrin complexes,^[^ ^3^, ^4^, ^5^, ^6^ ^]^ therefore hold promise for quantum sensing and quantum information processing, yet significant challenges remain. A key obstacle is the spatially precise integration of qubit candidates into well‐defined arrays, eventually comprising thousands or millions of qubits. Meeting this challenge will require further advances in organic synthesis^[^ ^7^, ^8^, ^9^ ^]^ and dynamic covalent^[^ ^10^, ^11^ ^]^ as well as supramolecular self‐assembly.^[^ ^12^, ^13^, ^14^, ^15^, ^16^ ^]^

Conjugated nanohoops have been extensively studied to understand their properties and to explore applications in organic electronics or biomedical imaging.^[^ ^17^, ^18^, ^19^, ^20^, ^21^, ^22^, ^23^, ^24^, ^25^, ^26^, ^27^, ^28^ ^]^ Relatively large (*d* = 2.5–5 nm) diyne‐linked six‐ and ten‐porphyrin macrocycles have been previously investigated by Anderson and Timmel, revealing quantum interference across two conjugation paths^[^ ^29^ ^]^ and hyperfine coupling with template ligands.^[^ ^30^, ^31^ ^]^ However, beyond host‐guest chemistry,^[^ ^20^, ^32^, ^33^ ^]^ the conformational rigidity of smaller *para*‐phenylene‐porphyrin nanohoops has not yet been exploited to position molecular qubit candidates in precise relative location and orientation.

Herein, we report the synthesis of a conjugated nanohoop with a well‐defined diameter of ca. 21 Å, comprising three Cu(II)‐porphyrins (*S* = ½) that are linked by three phenylene‐anthracene‐phenylene bridges. Comparison with suitable reference compounds revealed that the rigid structure not only influences the optical properties (Soret and Q bands) but also gives rise to well‐defined dipolar coupling and a rare case of light‐induced polarization of a doublet ground state.

**Cu[3]CPTA** was synthesized following a route previously developed for nickel(II) porphyrin macrocycles (Figure 1a).^[^ ^34^ ^]^ Two key building blocks were prepared via standard procedures:^[^ ^34^, ^35^, ^36^, ^37^, ^38^ ^]^ a quinoidal anthracene precursor (**1**) containing two boronic pinacol esters (Bpin), and a free‐base porphyrin with two bromine substituents in *trans* position (**2**). Suzuki–Miyaura cross‐coupling and removal of the triethylsilyl (TES)‐protecting groups furnished macrocyclic product **CPTA‐OH** that was isolated and fully characterized. **CPTA‐OH** served as a platform for metal coordination, enabling the modular synthesis of **[metal]CPTA‐OH** complexes. Stirring Cu(OAc)_2_ with **CPTA‐OH** in a CHCl_3_/CH_3_OH mixture at 50 °C led to quantitative formation of the paramagnetic **Cu‐CPTA‐OH** macrocycle. Aromatization of **Cu‐CPTA‐OH** was accomplished by heating the complex in acetic acid with NaH_2_PO_4_ and NaI at 100 °C, yielding the fully conjugated nanohoop **Cu[3]CPTA**. A porphyrin‐anthracene triad, **CuP1A2**, consisting of a copper‐coordinated porphyrin at the center and two anthracenes as “wings”, was synthesized as linear one‐spin reference compound (Scheme S2).

**Figure 1:**
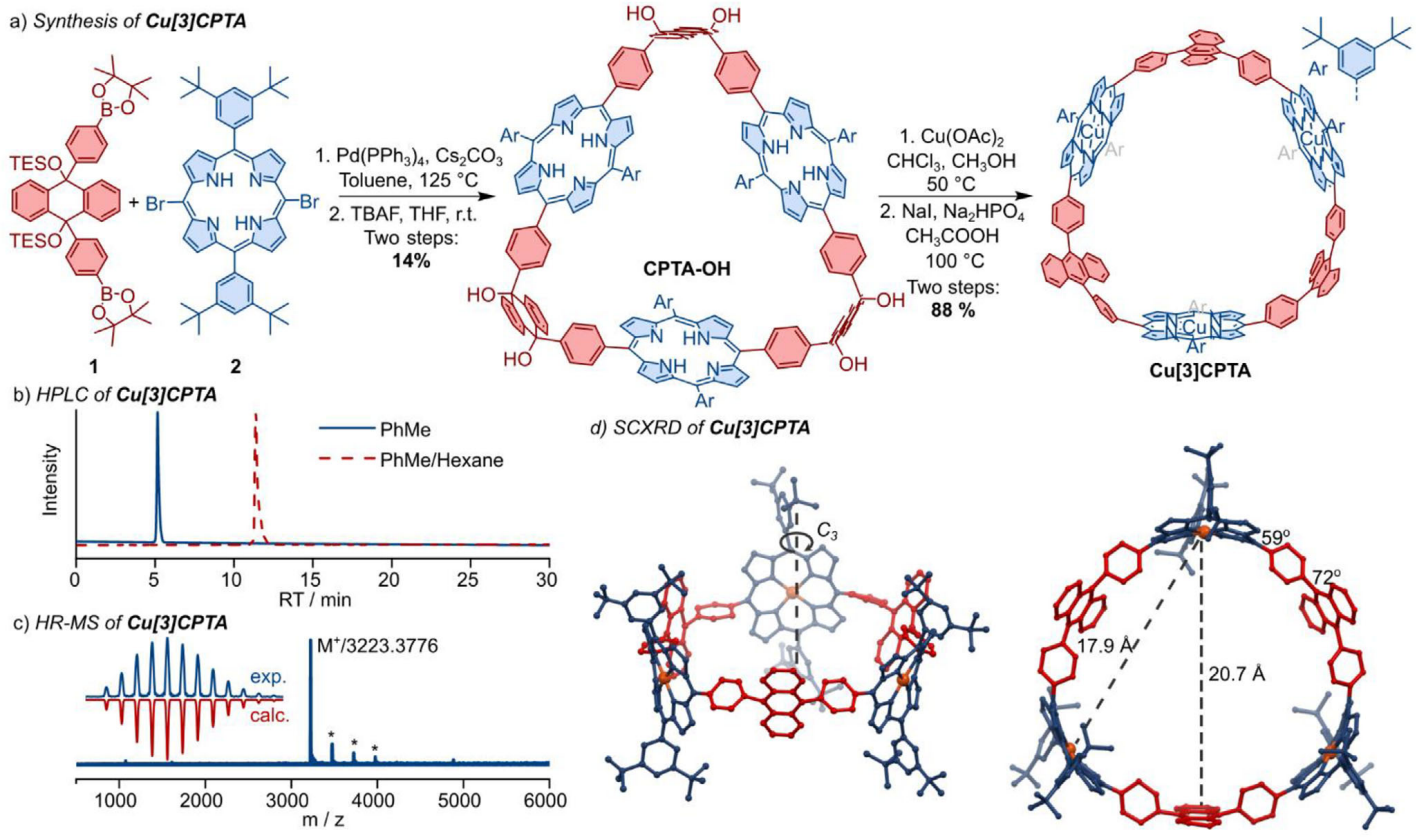
a) Synthesis of **Cu[3]CPTA**. b) HPLC chromatograms of purified **Cu[3]CPTA** in mobile phase toluene and toluene/hexane (7:3; normal phase, 0.5 mL min^−1^). c) MALDI mass spectrum (positive mode) of **Cu[3]CPTA**, including isotopic patterns. Peaks marked with asterisk correspond to [M + (n × matrix)]^+^. d) Molecular structure of **Cu[3]CPTA** as determined by SCXRD (space group: *P* 1 2_1_/*n* 1; CCDC number: 2425217).

Three paramagnetic compounds, **Cu‐CPTA‐OH**, **Cu[3]CPTA**, and **CuP1A2**, were thoroughly purified, analyzed by high‐performance liquid chromatography (HPLC; Figure 1b) and characterized by high‐resolution MALDI mass spectrometry (HR‐MALDI‐MS; Figures 1c and S1–S6). Single‐crystal (synchrotron) X‐ray diffraction (SCXRD) further confirmed the structure of **Cu[3]CPTA** (Figure 2d).^[^ ^39^ ^]^ The SCXRD data revealed a highly symmetric structure with a diameter of ca. 21 Å and quasi‐*D*_3h_ symmetry for the three porphyrin and anthracene units. The three Cu centers span approximately an equilateral triangle with three side lengths of ca. 18 Å (17.8, 17.9, 18.3 Å). These structural insights highlight the well‐defined architecture of the **Cu[3]CPTA** nanohoop, in which pairs of Cu spin centers are not only separated by a relatively precise distance but also have a precisely defined relative orientation within the three‐spin triangle (60°).

UV–vis absorption spectra show the typical redshift of nanohoop (**Cu[3]CPTA**) Soret and Q bands in comparison to non‐conjugated and non‐strained reference macrocycle **Cu‐CPTA‐OH** (Figure 2a). In contrast to free‐base precursors, the three Cu‐containing complexes exhibit no discernible photoluminescence.^[^ ^40^ ^]^ UV–vis transient absorption (TA) spectroscopy was performed upon Soret band excitation at 400 nm (Figure 2b,c, 300 K further details in the Supporting Information). As expected for a metalloporphyrin, ultrafast intersystem crossing populates a ^3^(π–π*) state localized on the porphyrin ligand. Thus, the TA spectra at 1 ps after excitation are dominated by the triplet features, namely two broad excited state absorption (ESA) regions between 440 to 535 nm and above 570 nm. In addition to the ESA features, the bleach of the Q(1,0) band is apparent in the spectra.^[^ ^41^, ^42^ ^]^ The triplet features decay to yield a new, narrow ESA feature at ca. 430 nm on a timescale of 100 ps (τ  =  66  ± 1 ps and 61 ± 1 ps for **Cu[3]CPTA** and **Cu‐CPTA‐OH**, respectively). In the literature, similar spectral signatures have been assigned to the formation of a triplet exciplex due to axial ligation of a solvent molecule to the copper centers.^[^ ^42^, ^43^ ^]^ As a consequence of the axial solvent ligation, the copper center is structurally distorted, which opens the possibility for energy transfer between the porphyrin ligand and the copper center (yielding a “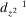, 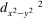
” metal‐centered excited 2(d,d) state). This electronic configuration is ascribed to the comparably long‐lived species in our spectra. Nonetheless, in 2‐MeTHF the corresponding signals decay on a sub‐ns timescale with time constants of 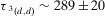 ps and 318 ± 10 ps for **Cu[3]CPTA** and **Cu‐CPTA‐OH**, respectively. Despite evidence for delocalization of the ^3^(π,π*) state in the nanohoop (see Supporting Information section 6.2 and 6.3), the overall lifetime of the excited states remain short compared to spin relaxation (vide infra).

**Figure 2:**
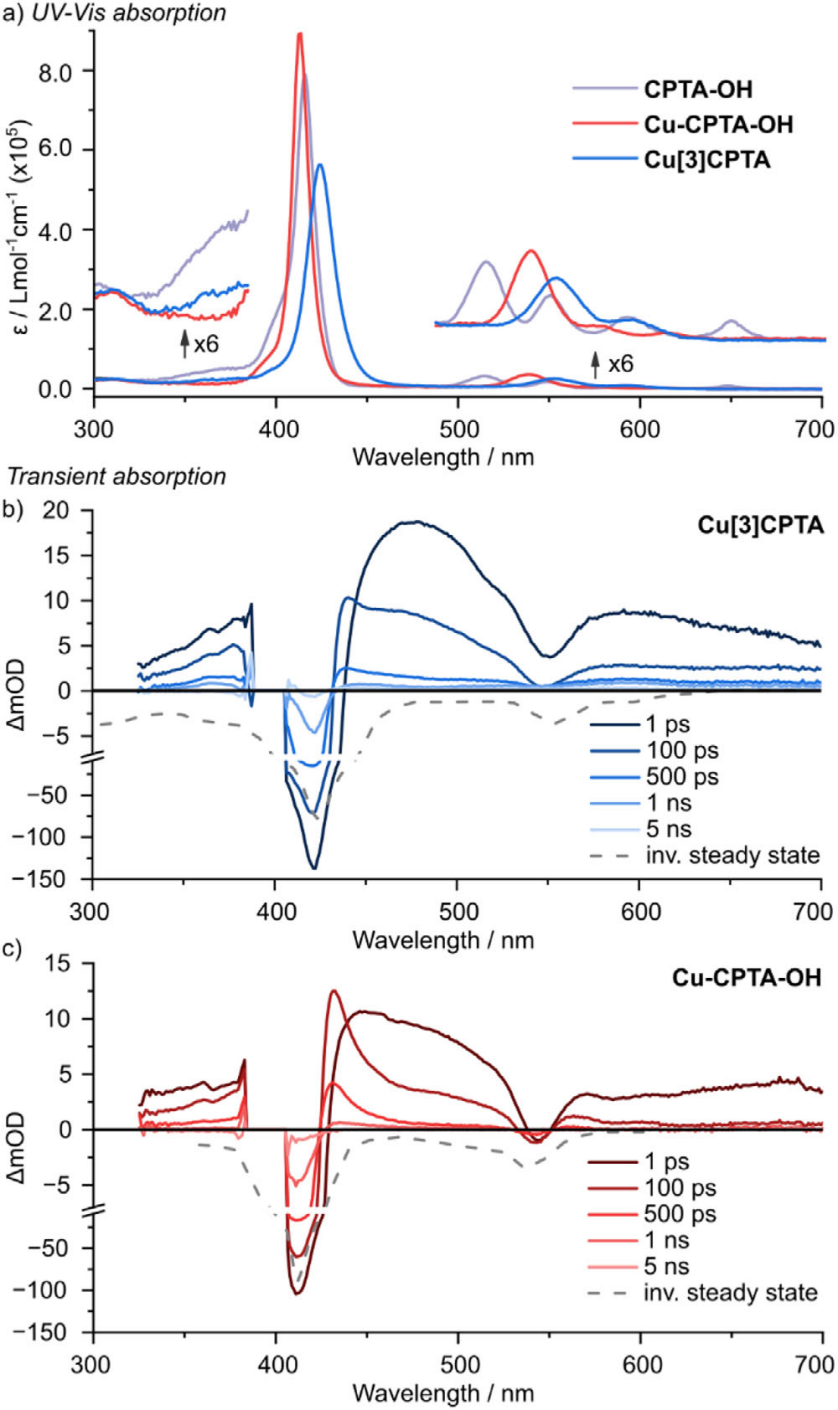
a) UV–vis absorption spectra of **CPTA‐OH**, **Cu‐CPTA‐OH**, and **Cu[3]CPTA** (2‐MeTHF, 5.0 × 10^−7 ^mol L^−1^, r.t.). b) and c) Transient absorption spectra of **Cu[3]CPTA** and **Cu‐CPTA‐OH** in 2‐MeTHF (2.2 × 10^−6^ mol L^−1^ and 1.6 × 10^−6^ mol L^−1^, resp., r.t.).

The magnetic interactions and macrocycle geometries of the paramagnetic copper porphyrin compounds were studied using continuous‐wave (cw) and pulse electron‐paramagnetic resonance (EPR) techniques. The cwEPR spectra of **CuP1A2**, **Cu‐CPTA‐OH**, and **Cu[3]CPTA**, recorded at the X‐band, are shown in Figure 3a. The spectrum of **CuP1A2** is consistent with axial Cu(II) compounds.^[^ ^30^, ^44^, ^45^ ^]^ The additional broadening observed for **Cu‐CPTA‐OH** and **Cu[3]CPTA** likely arises from the intramolecular electron–electron interactions. The coincidence of the peak positions in **CuP1A2** and **Cu‐CPTA‐OH** suggests highly conserved *g*‐ and hyperfine interaction matrices and the differences observed for **Cu[3]CPTA** might be attributed to bending of the porphyrin units within the nanohoop structure,^[^ ^30^ ^]^ as revealed by SXCRD analysis.

**Figure 3:**
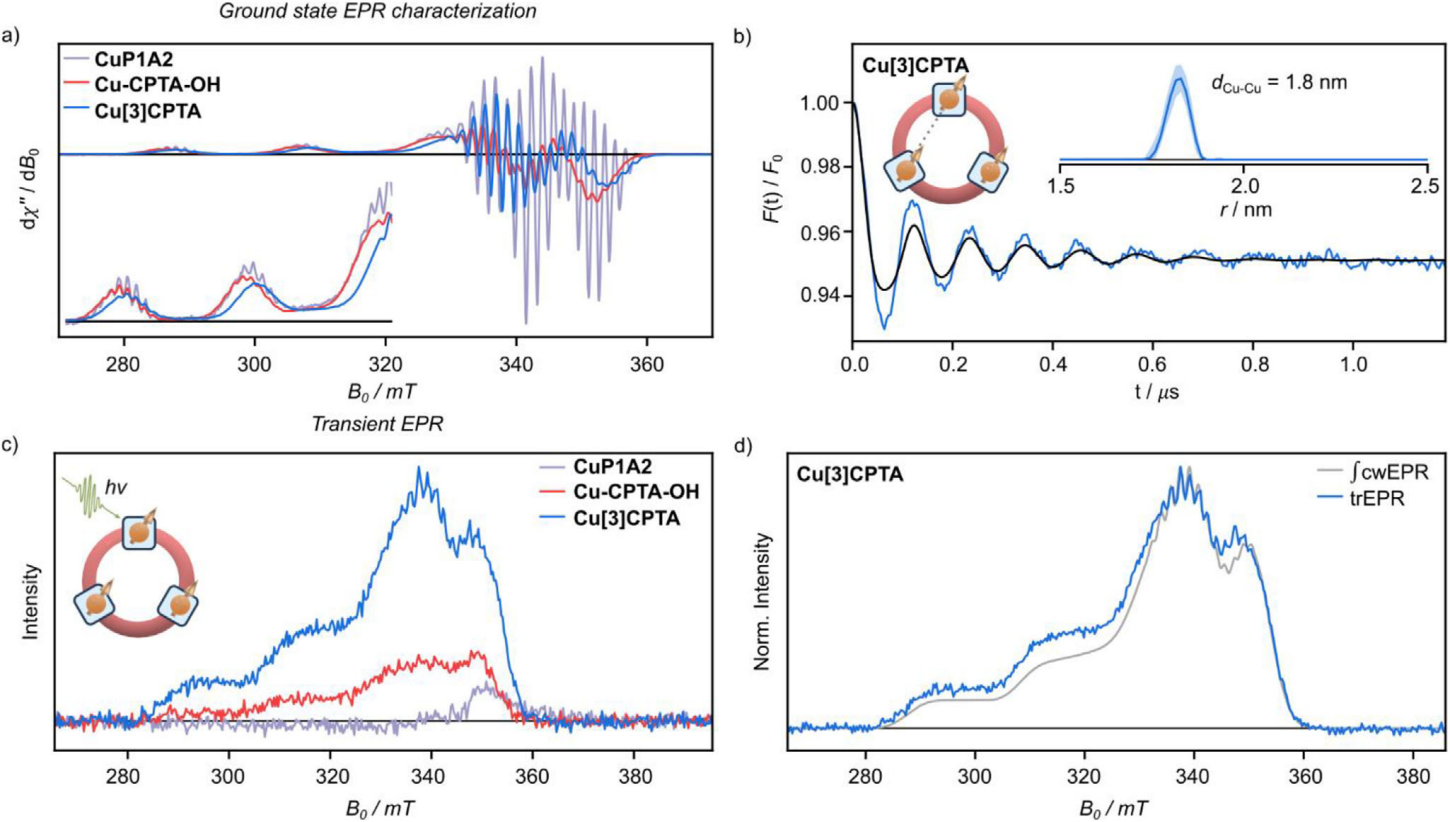
a) Comparison of the X‐band frequency (9.75 GHz) frozen solution (80 K) cwEPR spectra of **CuP1A2**, **Cu‐CPTA‐OH**, and **Cu[3]CPTA**. b) Experimental four‐pulse DEER trace recorded at Q‐band frequencies and 15 K together with a fit (black) and distance distribution (inset) obtained from DeerAnalysis for **Cu[3]CPTA**. c) Comparison of the X‐band frequency frozen solution (20 K) trEPR spectra of **CuP1A2**, **Cu‐CPTA‐OH**, and **Cu[3]CPTA** following photoexcitation at 554 or 540 nm. The data were acquired with equivalent LASER pulse energies, microwave power, and signal averaging, using samples prepared to the same molecular concentration (≈150 µM); the spectra are scaled to equivalent noise‐levels of the off‐resonant field positions. d) Comparison of the numerically integrated field‐modulated cwEPR and trEPR spectrum (400–500 ns) of **Cu[3]CPTA** following photoexcitation at 554 nm.

Phase‐memory times up to a few microseconds were found for all systems in protonated solvents, presented in the Supporting Information alongside spin—lattice relaxation data, which are comparable to the relaxation rates of copper(II) in monomeric organic complexes attributed to the small magnetic dipole–dipole modulation, associated with the low temperature and relatively small dipolar coupling.^[^ ^46^, ^47^, ^48^, ^49^ ^]^ For **Cu[3]CPTA** at 15 K in the *g*_⊥_ region, *T*_m_ = 2.2 and 4.4 µs, for 2‐MeTHF and toluene‐*d*_8_ solutions, respectively, these values compare favorably with metal‐based molecular two‐qubit systems and a flexible molecular three‐qubit system.^[^ ^50^, ^51^, ^52^, ^53^, ^54^ ^]^ Improvements to the reported decoherence times may be realized by further tuning of the solvent and by (selective) isotopic substitution or removal of the intrinsic protons, in particular those that reside outside the so‐called diffusion barrier.^[^ ^52^, ^55^, ^56^ ^]^ Power‐dependent Rabi nutation experiments were performed at fields in the *g*_⊥_ and *g*_∥_ regions (Figures S42–S47). In each case, the observed Rabi frequency varied linearly with the driving microwave field strength, indicating the oscillations are pure Rabi type, thus demonstrating that our systems can be manipulated coherently and placed into any arbitrary superposition, as required for quantum gate operations.

Four‐pulse double electron–electron resonance (DEER) measurements were performed at 15 K at the Q‐band to investigate the separation and orientation of the Cu centers in **Cu‐CPTA‐OH** and **Cu[3]CPTA**. Figure 3b presents the experimental DEER data for **Cu[3]CPTA** with pump and detection pulses set to excite spins in the *g*_⊥_ region. Given the low modulation depth, it is assumed that the observed modulation arises from the pair contribution only.^[^ ^57^ ^]^ The data display a well‐defined modulation and were considered using a model‐free analysis as implemented in DeerAnalysis.^[^ ^58^ ^]^ The obtained distance distribution, with a maximum at 1.8 nm, is in good agreement with the SCXRD data, suggesting that both the effects of orientation selection on the distance distribution and the presence of an exchange interaction between the copper centers are negligible. The narrow width of the distance distribution (FWHM < 0.1 nm) confirms that the molecular ring structure is fairly rigid in solution and the inter‐spin distances well defined. Additional analysis considering orientation selection is discussed in the Supporting Information. The shorter period and larger damping of the dipolar modulation in DEER data obtained for **Cu‐CPTA‐OH**, see Supporting Information, is consistent with theoretical simulations that indicate a structural flexibility giving rise to a distribution of geometries with considerably smaller copper–copper separations. The two qubit gate time can be determined from the first minimum in the dipolar time‐trace, for **Cu[3]CPTA** this is 64 ns which compares favorably with the phase‐memory time and the single‐qubit manipulation time (dependent on the available microwave power, typically low tens of nanoseconds).^[^ ^59^ ^]^

Following photo‐excitation, the three copper systems display a weak light‐induced signal observed using direct‐detection transient cwEPR (trEPR, Figure 3c). The transient signals are in absorption and resemble the EPR spectrum of a Cu(II)‐porphyrin doublet ground state. To illustrate this similarity, the trEPR spectrum obtained for **Cu[3]CPTA** is compared with the integrated field‐modulated cwEPR spectrum in Figure 3d, clearly confirming the observation of ground state spin polarization upon photoexcitation of the copper nanohoop. The light‐induced spin‐polarization decays according to the *T*_1_ lifetime of the ground‐state copper center.

A light‐induced polarization of a doublet ground state has only rarely been observed in solid matrices and the context of molecular qubit candidates^[^ ^60^, ^61^, ^62^ ^]^ but is sought‐after as it provides an attractive way of initializing the qubit system in a pure and well‐defined spin state. The formation of ground‐state polarization is suggested to occur via spin‐selective population and depopulation of a transient triplet–doublet pair excited state followed by decay to the ground state, where electronic transitions occur on timescales faster than the spin relaxation.^[^ ^60^, ^63^, ^64^ ^]^ Several previous reports on light‐induced polarization of doublet ground‐states have interpreted the observations via the reversed quartet mechanism; however, a range of signs and magnitudes in the observed polarization effects have been demonstrated.^[^ ^60^, ^64^, ^65^, ^66^, ^67^, ^68^, ^69^, ^70^, ^71^, ^72^ ^]^ The exact mechanism for the ground‐state polarization is an open question that relies on a detailed knowledge of the electronic structure of the excited states, including spin–orbit coupling effects.^[^ ^63^, ^64^, ^73^, ^74^ ^]^ A global kinetic analysis of the transient absorption data, Figure 2 and Supporting Information, supports this scenario also for the investigated copper structures; although our data is limited to the solution state (at 150 K, 225 K, 240 K, or 293 K) and slightly below the solvents freeze point at 120 K, it is reasonable to assume that the excited state lifetimes of the investigated copper structures at 20 K (∼0.3 ns at r.t.) will still be significantly shorter compared to typical ground state spin relaxation times of copper porphyrins at the same temperature (*T*_m_ ∼2 µs, *T*_1_ ∼2 ms).^[^ ^46^ ^]^

To explore the influence of the molecular geometry on light‐induced ground state spin polarization, the signal amplitudes obtained for the three paramagnetic structures are compared in Figure 3c. For data recorded with the same number of averages and LASER pulse energies, the largest signal is obtained for **Cu[3]CPTA**, which may suggest that a rigidified macrocyclic structure with well‐defined orientation between the spin centers is beneficial for the generation of a large ground state spin polarization.

In conclusion, we present a molecular design concept for the structurally precise assembly of a discrete number of qubit candidates into an exceptionally rigid ring. The approach is complementary to the integration of qubit candidates into rigid, yet periodic frameworks (COFs or MOFs). The shape‐persistence of strained nanohoop **Cu[3]CPTA** enabled the demonstration of a two‐qubit operation in a DEER experiment, while also giving rise to enhanced light‐induced spin polarization. Further work will be needed to better understand the effects of strain‐induced shape‐persistence in macrocycles and thus develop general design principles in this emerging field.

## Supporting Information

Synthesis and characterization data, supplementary transient absorption, and EPR data are included in Supporting Information. Computational data are openly available via Zenodo under https://zenodo.org/records/15365308, reference number 15365308.

## Conflict of Interests

The authors declare no conflict of interest.

## Supporting information



Supporting Information

Supporting Information

## Data Availability

The data that support the findings of this study are openly available in Zenodo at https://zenodo.org/records/15365308 and reference number 15365308.
