# Supplementary material for: Rigidifying Qubit Candidates in a Cu‐Porphyrin Nanohoop: Dipolar Coupling in Spin Pairs and Spin‐Polarized Ground State
Source: Angew Chem Int Ed Engl. 2025 Dec 9;65(4):e22950. doi: 10.1002/anie.202522950 (PMC12828445; doi:10.1002/anie.202522950)
Supplement: Supplementary file 1 — Supporting Information [file ANIE-65-e22950-s002.pdf]

Supporting Information

# Rigidifying Qubit Candidates in a Cu-Porphyrin Nanohoop: Dipolar Coupling in Spin Pairs and Spin-Polarized Ground State

Xingmao Chang, Ashley J. Redman, Linda Zedler, Louis Blechschmidt, Adriana Sacristán-Martín, Fabian Schwer, Inhar Imaz, Markus P. B. Wiedmaier, Xavi Ribas, Daniel Maspoch, Benjamin Dietzek-Ivanšić,* Sabine Richert,* and Max von Delius*

Table of Contents


1. Materials and instrumentation ..... 3
2. Synthesis and characterization of compounds ..... 4
   2.1 Overview of synthesis ..... 4
   2.2 Synthesis of compound **1** ..... 5
   2.3 Synthesis of compound **2** ..... 7
   2.4 Synthesis of **CPTA-OH** ..... 8
   2.5 Synthesis of **Cu[3]CPTA** ..... 9
   2.6 Synthesis of compound **3** ..... 10
   2.7 Synthesis of **P1A2** ..... 10
   2.8 Synthesis of **Cu-P1A2** ..... 11
3. HR-MS and HPLC of paramagnetic compounds ..... 12
   3.1 Comparisons of HR-MS of compounds ..... 12
   3.2 HPLC of paramagnetic compounds ..... 14
4. X-Ray crystallography data (**Cu[3]CPTA**) ..... 17
5. Steady optical properties ..... 19
6. Transient absorption studies ..... 20
   6.1 Ultrafast transient absorption spectroscopy ..... 20
   6.2 Excited states dynamics ..... 23
   6.3 Lower temperature TA-Spectroscopy ..... 24
7. EPR measurements ..... 29
   7.1 Materials and methods for EPR part ..... 29
   7.2 Additional cwEPR data ..... 32
   7.3 Additional trEPR data ..... 32
   7.4 Relaxation time measurements ..... 35
   7.5 Additional DEER data ..... 36
   7.6 Rabi nutations ..... 43
8. DFT calculations ..... 47
9. Supplementary NMR and mass spectra ..... 48
10. References ..... 57

## 1. Materials and instrumentation

All reagents and solvents were commercially available and used without further purification. Anhydrous solvents were dried prior to use in an MBraun SPS-800 instrument.

NMR data were acquired on a Bruker 400 MHz AVANCE spectrometer in $CHCl_3$-$d_1$, THF-$d_8$, DMSO-$d_6$, or $CH_2Cl_2$-$d_2$.

HRMS (MALDI-TOF) data were recorded on a Bruker Daltonic Autoflex maX using trans-2-[3-(4-tert-Butylphenyl)-2-methyl-2-propenylidene]malononitrile (DCTB) as matrix.

HPLC experiments were performed on a Shimadzu 2020 HPLC-MS equipped with a Buckyprep M column with flow rate 0.5 mL/min.

UV-Vis absorption spectra were recorded on a Perkin Elmer Lambda 365 instrument. Photoluminescence measurements were carried out on a PerkinElmer FL6500 instrument.

All information for SCXRD, transient absorption, and EPR measurements are provided in Sections 4, 6, and 7, respectively.

## 2. Synthesis and characterization of compounds

### 2.1 Overview of synthesis

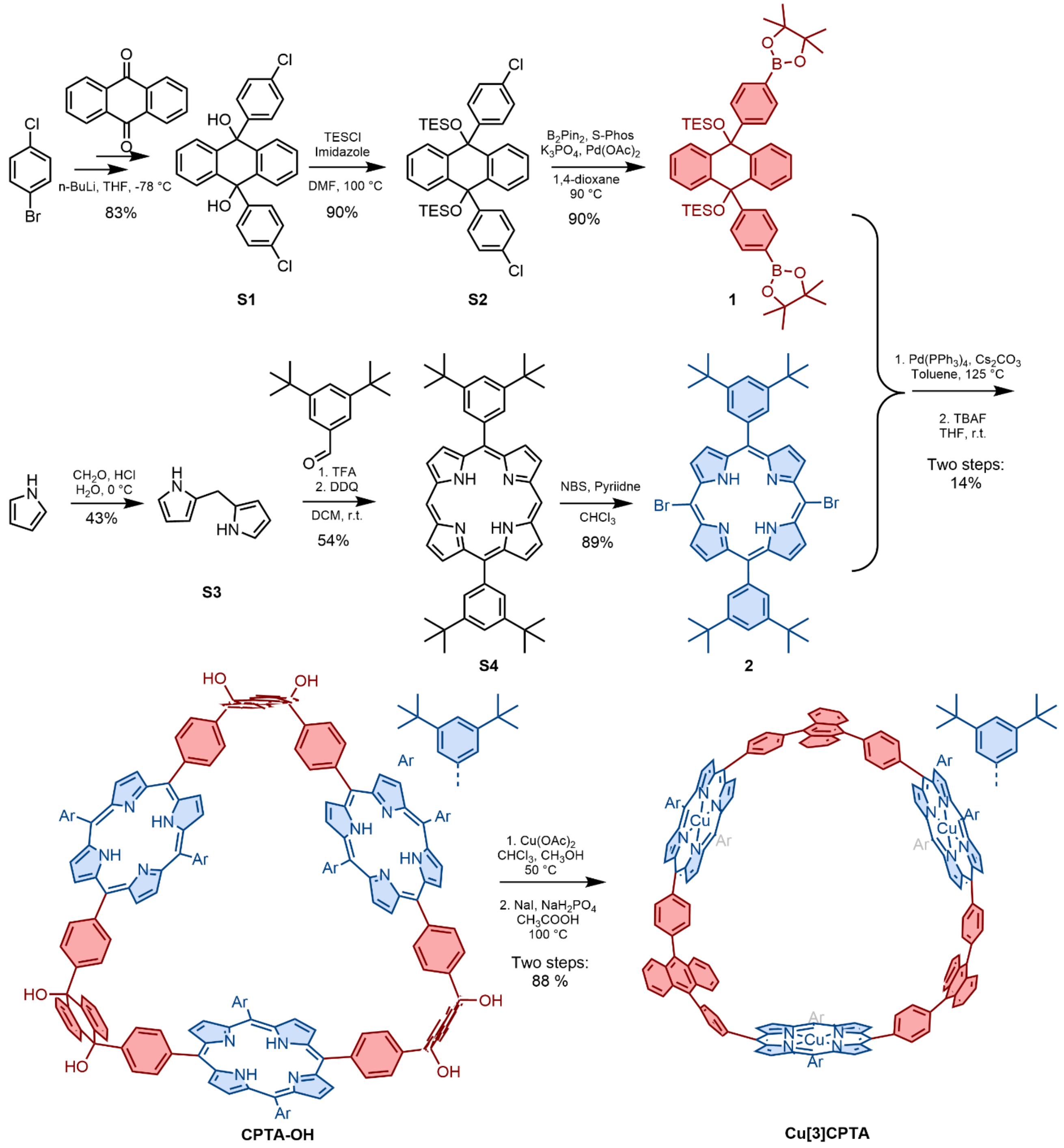


**Scheme S1** Synthesis of **Cu[3]CPTA**

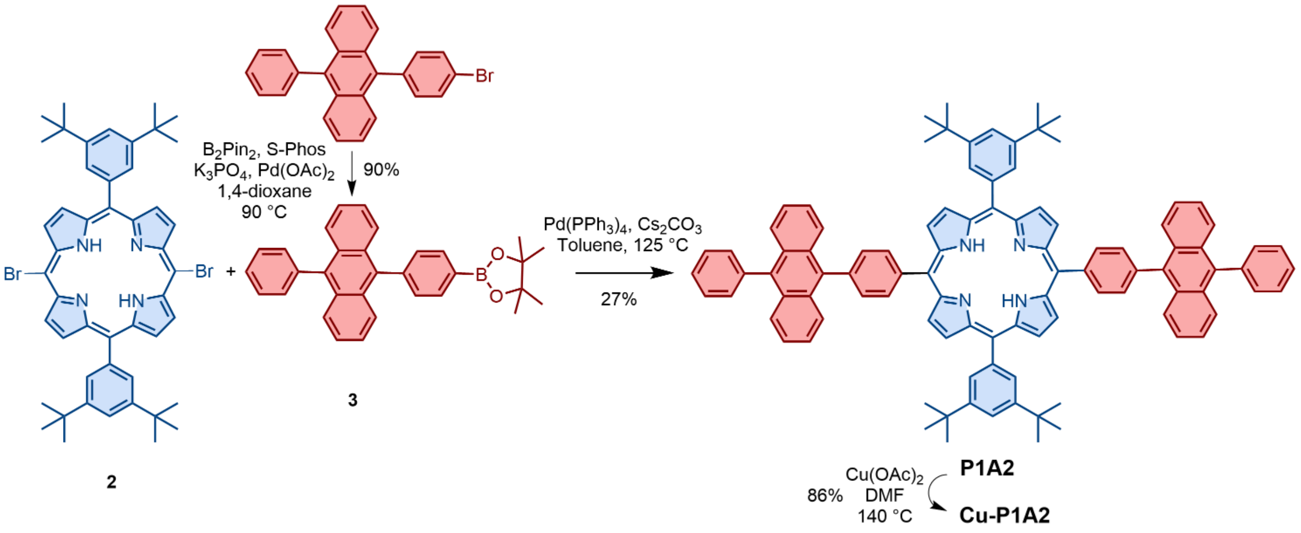


**Scheme S2** Synthesis of **Cu-P1A2**

2.2 Synthesis of compound **1**

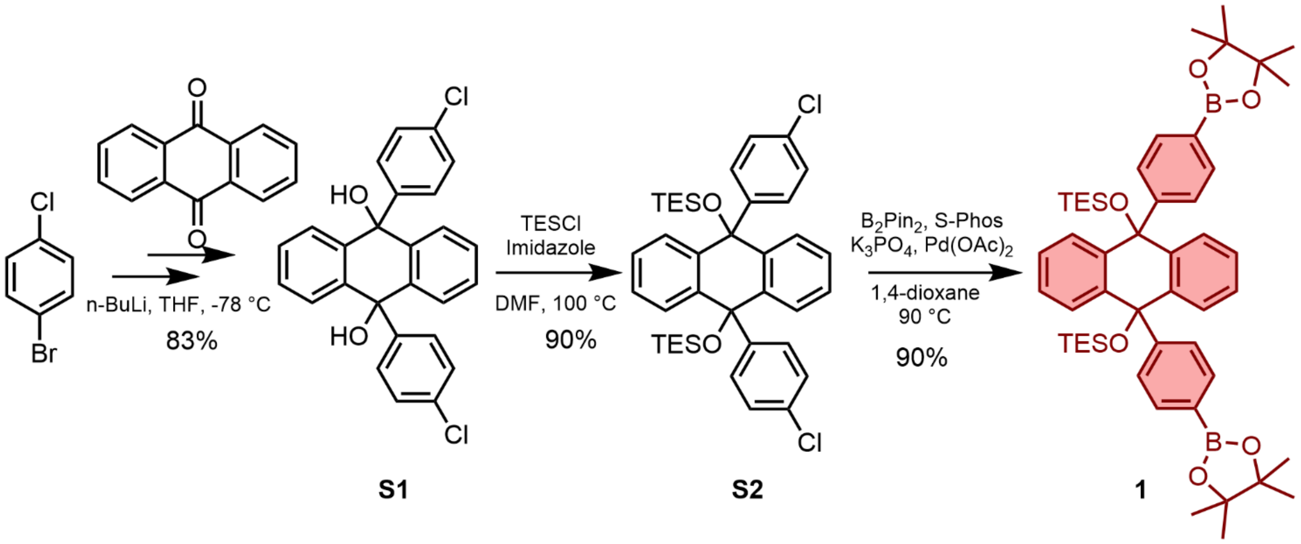


Compound **1** was synthesized based on the method outlined in our previous paper[1] with several modifications to improve the process.

*Synthesis of **S1***

1-Chloro-4-bromobenzene (5.8 g, 30.0 mmol, 3.0 equiv.) was dissolved in degassed, anhydrous THF (100 mL) in a Schlenk flask under argon atmosphere. To this solution, *n*-BuLi (2.5 mol/L, 12.0 mL, 30.0 mmol, 3.0 equiv.) was added dropwise at -78 °C. The mixture was stirred for 1 hour at this temperature before anthraquinone (2.1 g, 10.0 mmol, 1.0 equiv.) was added under argon atmosphere. The reaction was stirred overnight, gradually warming to room temperature. The reaction was quenched with methanol (10 mL), and the solvents were removed under reduced pressure. The residue was washed sequentially with water (3×50 mL) and dichloromethane (1×50 mL). After drying under vacuum, pure compound **S1** was obtained as a white solid (3.6 g, 83%).

**$^1$H NMR** (400 MHz, DMSO-$d_6$, 298 K): δ (ppm) = 7.80-7.72 (dd, 4H), 7.45-7.39 (dd, 4H), 6.97-6.91 (m, 4H), 6.73-6.67 (m, 4H), 6.42-6.38 (s, 2H).

**$^{13}$C NMR** (100 MHz, DMSO-$d_6$, 298 K): δ (ppm) = 144.41, 141.02, 131.00, 129.00, 127.21, 126.97, 126.61, 73.03.

*Synthesis of **S2***

**S1** (1.1 g, 2.5 mmol, 1.0 equiv.) and Imidazole (408 mg, 6 mmol, 2.4 equiv.) were dissolved in anhydrous DMF (15 mL) in a Schlenk flask under argon atmosphere. To this solution, Cholorotriethylsilane (TESCl, 1.0 mL, 6.0 mmol, 2.4 equiv.) was added dropwise at room temperature. The mixture was stirred overnight at 100 °C under argon atmosphere. The solvent was removed under reduced pressure. The residue was dissolved with DCM (50 mL), washed with water (3×50 mL), dried over $MgSO_4$, filtered, removed solvent by reduced pressure, then yielded compound **S2** as brown oil (1.5 g, 90%).

**$^1$H NMR** (400 MHz, $CDCl_3$, 298 K): δ (ppm) = 7.63-7.56 (dd, 4H), 7.32-7.27 (dd, 4H), 7.06-6.95 (m, 8H), 0.90-0.83 (t, 18H), 0.45-0.35 (q, 12H). 13C NMR (101 MHz, CDCl3) δ 148.47, 139.53, 132.47, 128.46, 127.79, 127.54, 77.34, 77.03, 76.71, 76.07, 7.26, 6.56.

**$^{13}$C NMR** (100 MHz, $CDCl_3$, 298 K): δ (ppm) = 148.61, 139.66, 132.60, 128.59, 127.92, 127.67, 76.20, 7.39, 6.70.

*Synthesis of **1***

**S2** (2.6 g, 4 mmol, 1 equiv.), Bis(pinacolato)diboron (10 g, 40 mmol, 10 equiv.), $K_3PO_4$ (5 g, 24 mmol, 6 equiv.), S-Phos (328 mg, 0.8 mmol, 0.2 equiv.), and $Pd(OAc)_2$ (90 mg, 0.4 mmol, 0.1 equiv.) were mixed in a Schlenk flask under argon atmosphere. The anhydrous dioxane (15 mL) was added to the flask, and the mixture was stirred at 90 °C under argon for two days. After cooling to room temperature, the reaction mixture was passed through a short column of active charcoal. The solvent was removed under reduced pressure, and the residue was washed with methanol (100 mL), yielding compound **1** as a white solid (3.0 g, 90%).

**$^1$H NMR** (400 MHz, $CDCl_3$, 298 K): δ (ppm) = 7.65-7.60 (m, 4H), 7.55-7.50 (dd, $J$ = 6.1, 3.4 Hz, 4H), 7.32-6.29 (m, 4H), 7.23-7.17 (dd, $J$ = 6.1, 3.4 Hz, 4H), 1.35-1.30 (s, 24H), 0.92-0.86 (t, $J$ = 7.9 Hz,18H), 0.46-0.36 (q, $J$ = 7.9 Hz, 12H).

**$^{13}$C NMR** (100 MHz, $CDCl_3$, 298 K): δ (ppm) = 154.31, 139.55, 134.26, 129.26, 127.73, 126.28, 83.77, 76.30, 24.99, 7.41, 6.65.

2.3 Synthesis of compound **2**

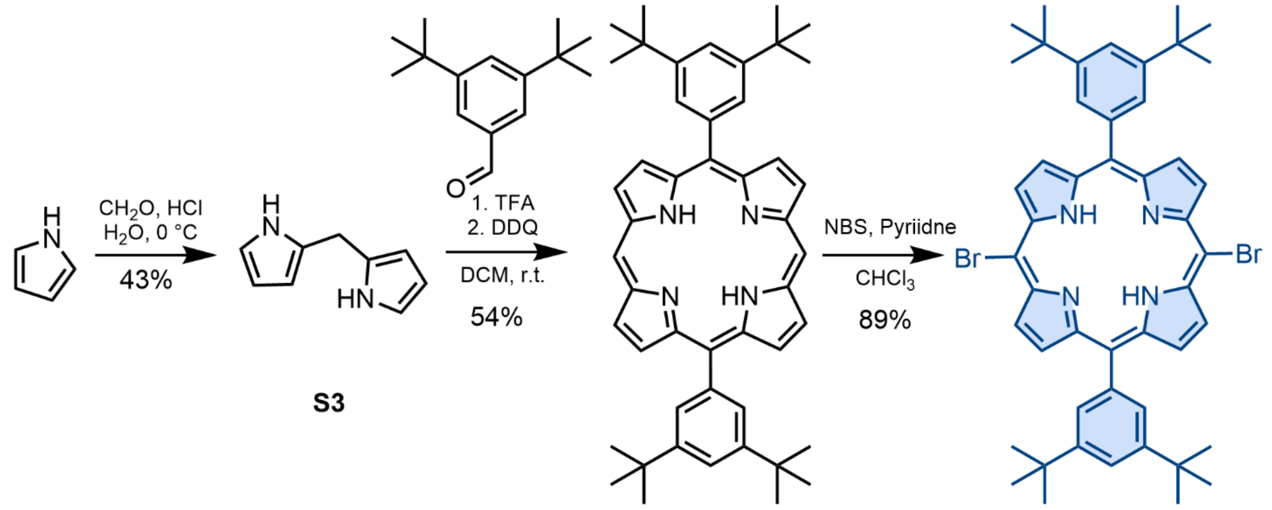


S3 S4 2

Compound **2** was synthesized according to our previous work with some changes.[2]

*Synthesis of compound **S3***

Freshly distilled pyrrole (17.0 mL, 246 mmol, 4.1 equiv.) was dissolved in water (60 mL) and degassed for 30 minutes via ultrasonication under argon. Formaldehyde (4.5 mL, 37% in water, 60 mmol, 1.0 equiv.) and HCl (0.2 mL, 35%, 2 mmol, 0.03 equiv.) were added dropwise at 0 °C. The reaction mixture was stirred at 0 °C for 2.5 hours. The resulting solution was extracted with dichloromethane (DCM, 3 × 50 mL). The organic layers was dried over $MgSO_4$ and then concentrated under reduced pressure to obtain the crude product. The crude product was purified by distillation, with the desired fraction collected at 0.003 mbar and 95 °C, yielding compound **S3** as a slightly yellow solid (3.76 g, 25.7 mmol, 43%). Compound **B1** was stored in the refrigerator to prevent decomposition.

**$^1$H NMR** (400 MHz, $CDCl_3$, 298 K): δ (ppm) = 8.15-7.73 (s, 2H), 6.72-6.67 (dd, *J* = 2.7, 1.6 Hz, 2H), 6.22-6.13 (q, *J* = 2.9 Hz, 2H), 6.09-6.03 (dddt, *J* = 3.4, 2.5, 1.6, 0.8 Hz, 2H), 4.06-3.97 (s, 2H).

**$^{13}$C NMR** (100 MHz, $CDCl_3$, 298 K): δ (ppm) = 129.19, 117.43, 108.50, 106.54, 26.49.

*Synthesis of compound **S4***

3,5-Di-tert-butylbenzaldehyde (1.80 g, 8.23 mmol, 1.0 equiv.) and compound **S3** (1.21 g, 8.27 mmol, 1.0 equiv.) were dissolved in degassed DCM (1200 mL). Trifluoroacetic acid (TFA, 0.24 mL, 3.1 mmol, 0.38 equiv.) was added slowly to the solution. The reaction mixture was stirred in the dark for 2 hours. DDQ (2.36 g, 10.4 mmol, 1.26 equiv.) was added under argon, and the reaction was stirred for an additional hour. The reaction was quenched by adding triethylamine (0.7 mL). The solvent was removed under reduced pressure, and the crude product was purified by column chromatography (petroleum ether/DCM, from 4:1 to 2:1). This yielded compound **S4** as a red solid (1.52 g, 2.21 mmol, 54%).

**$^1$H NMR** (400 MHz, $CDCl_3$, 298 K): δ (ppm) = 10.35-10.29 (s, 2H), 9.44-9.37 (d, *J* = 4.6 Hz, 4H), 9.17-9.11 (d, *J* = 4.6 Hz, 4H), 8.18-8.13 (d, *J* = 1.8 Hz, 4H), 7.87-7.82 (t, *J* = 1.9 Hz, 2H), 1.62-1.54 (s, 36H), -2.97--3.04 (s, 2H).

**$^{13}$C NMR** (100 MHz, $CDCl_3$, 298 K): δ (ppm) = 149.27, 147.60, 145.19, 140.54, 131.63, 131.41, 130.35,

121.24, 120.62, 105.22, 35.26, 31.94.

*Synthesis of compound **2***

Compound **S4** (805 mg, 1.17 mmol, 1.0 equiv.) was dissolved in chloroform (100 mL), and pyridine (0.3 mL, 3.49 mmol, 3.0 equiv.) was added. N-Bromosuccinimide (NBS, 477 mg, 2.68 mmol, 2.3 equiv.) was added stepwise, and the reaction mixture was stirred for 2 hours. The reaction was quenched by adding acetone (20 mL), and the solvent was removed under reduced pressure. The residue was washed with methanol (200 mL), yielding compound **2** as a red solid (881 mg, 1.04 mmol, 89%).

**$^1$H NMR** (400 MHz, $CDCl_3$, 298 K): δ (ppm) = 9.68-9.61 (d, *J* = 4.8 Hz, 2H), 8.95-8.85 (d, *J* = 4.7 Hz, 4H), 8.07-8.03 (d, *J* = 1.8 Hz, 4H), 7.88-7.83 (t, *J* = 1.8 Hz, 2H), 1.58-1.56 (s, 36H), -2.60--2.68 (s, 2H).

**$^{13}$C NMR** spectrum was not obtained due to the very poor solubility of **2**.

2.4 Synthesis of **CPTA-OH**

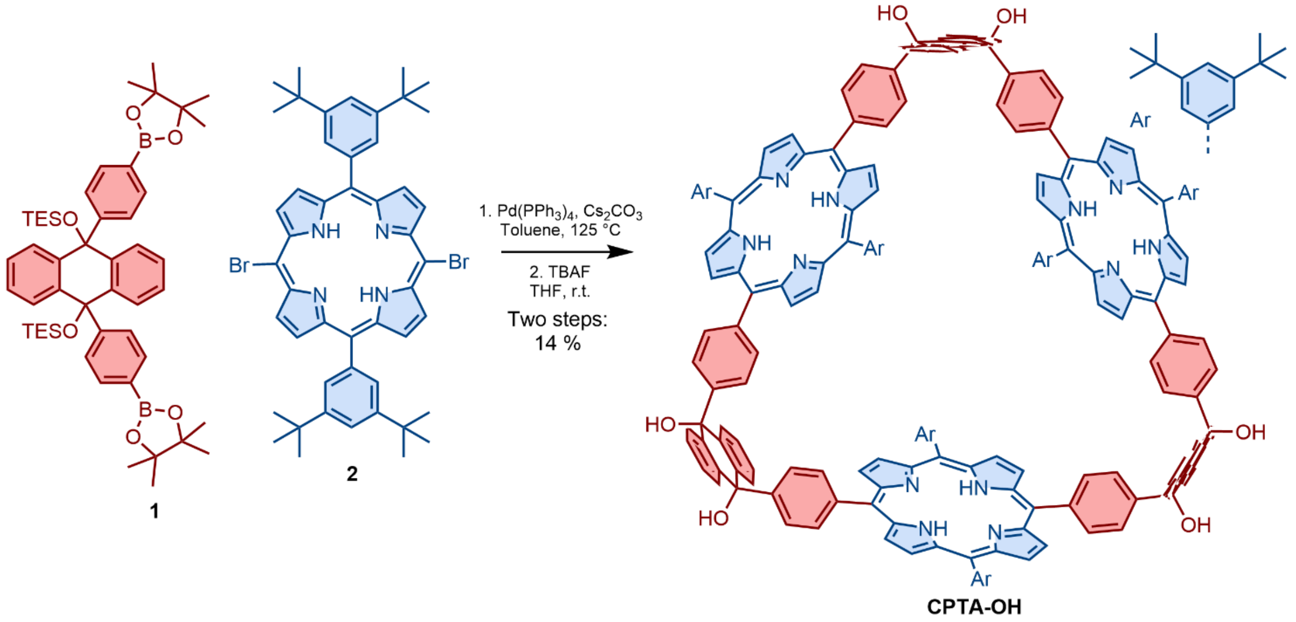


Compound **1** (236.5 mg, 0.28 mmol, 1.0 equiv.), compound **2** (236.5 mg, 0.28 mmol, 1.0 equiv.), $Pd(PPh_3)_4$ (64.7 mg, 0.056 mmol, 0.2 equiv.), and $Cs_2CO_3$ (365.0 mg, 1.12 mmol, 4 equiv.) were mixed in a Schlenk flask under argon atmosphere. Degassed toluene (200 mL) with one drop of water were added to the flask, and the mixture was stirred at 125 °C under argon for two days. After cooling to the room temperature, the mixture was passed through a short aluminum oxide column. The solvent was removed under reduced pressure, and the residue were used directly for deprotection without further purification.

The residue was dissolved in anhydrous THF (20 mL), and then TBAF (3.0 mL, 1.0 mol/L in THF) was added slowly. The reaction mixture was stirred at room temperature for 1 hours. The reaction was quenched by water (5.0 mL), and the solvents were removed under reduced pressure. The crude product was purified by silica gel column with mixture of DCM and EA (from 9:1 to 5:1). This yielded **CPTA-OH** as a red solid (40 mg, 14%). The Rf value of **CPTA-OH** on a TLC plate with the mixture of DCM and EA (8:1) was approximately 0.6.

**$^1$H NMR** (400 MHz, THF-$d_8$, 298 K): δ (ppm) = 8.51-8.46 (d, *J* = 4.9 Hz,12H), 8.46-8.39 (d, *J* = 4.8 Hz, 12H), 7.90-7.81 (m, 36H), 7.72-7.68 (d, *J* = 1.8 Hz,12H), 7.55-7.51 (t, *J* = 1.8 Hz, 6H), 7.38-7.31 (dq, *J* =

6.9, 3.6 Hz, 12 H), 5.67-5.61 (s, 6H), 1.17-1.10 (s, 108H), -3.04--3.17 (s, 6H).

**$^{13}$C NMR** (100 MHz, THF-d$_8$, 298 K): δ (ppm) = 150.68, 149.59, 142.46, 142.32, 141.52, 134.64, 130.90, 129.86, 128.64, 126.31, 121.96, 121.64, 120.49, 74.23, 35.64, 31.99.

**HRMS** (MALDI): Calculated for $C_{222}H_{210}N_{12}O_6$: 3141.6563; Found 3141.6543.

2.5 Synthesis of **Cu[3]CPTA**

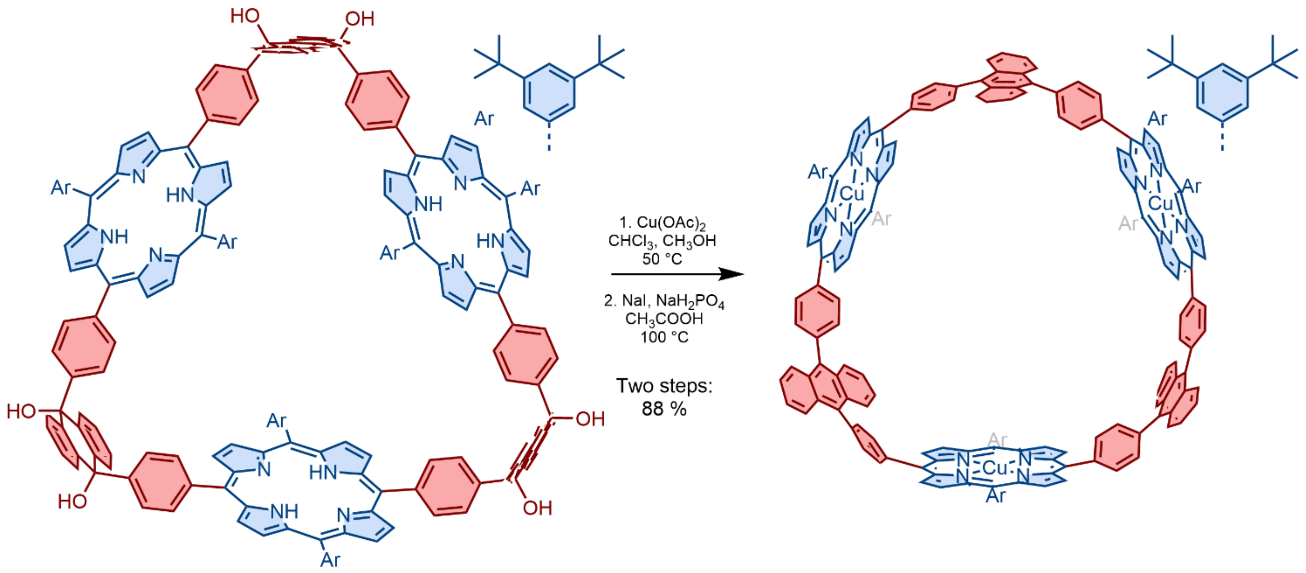


Compound **CPTA-OH** (31 mg, 0.01 mmol, 1 equiv.) and $Cu(OAc)_2$ (20 mg, 0.1 mmol, 10 equiv.) were dissolved in a mixture of $CHCl_3$ (8 mL) and $CH_3OH$ (2 mL) in a flask. The mixture was stirred at 50 °C under argon overnight. After cooling to the room temperature, the solvents were removed under reduced pressure. The residue was dissolved in THF, and the mixture was filtered by a short silica gel column. This yielded **Cu-CPTA-OH** as a red solid almost in quantitively (32 mg, 97%).

**HRMS** (MALDI): Calculated for $Cu_3C_{222}H_{204}N_{12}O_6$: 3326.3964; Found 3326.3950.

**HPLC**: See section 3.2.

The compound **CuCPTA-OH** (19 mg, 0.006 mmol, 1.0 equiv.), NaI (18.9 mg, 0.126 mmol, 21.0 equiv.), and $NaH_2PO_4$ (13.7 mg, 0.114 mmol, 20 equiv.) were mixed in a Schlenk flask under argon atmosphere. Degassed $CH_3COOH$ (15 mL) was added to the flask, and the mixture was stirred at 110 °C under argon for two days. After cooling to the room temperature, the solvents were removed under reduced pressure and the residue was purified by column with the mixture of DCM and Hexane (1:3) gave compound **Cu[3]CPTA** as a red solid (16 mg, 90%). The Rf value of **Cu[3]CPTA** on a TLC plate with the mixture of DCM and Hexane (1:3) was approximately 0.2.

**HRMS** (MALDI): Calculated for $Cu_3C_{222}H_{198}N_{12}$: 3224.3799; Found 3224.3788.

**HPLC**: See section 3.2.

2.6 Synthesis of compound **3**

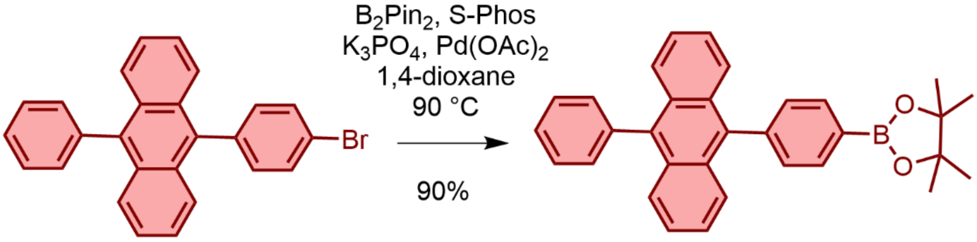


**3**

9-(4-Bromophenyl)-10-phenylanthracene (500 mg, 1.22 mmol, 1 equiv.), Bis(pinacolato)diboron (930 mg, 3.66 mmol, 3.0 equiv.), $K_3PO_4$ (778 mg, 3.66 mmol, 3.0 equiv.), S-Phos (50 mg, 0.12 mmol, 0.1 equiv.), and $Pd(OAc)_2$ (14 mg, 0.06 mmol, 0.05 equiv.) were mixed in a Schlenk flask under argon atmosphere. The degassed anhydrous dioxane (30 mL) was added to the flask, and the mixture was stirred at 90 °C under argon for 24 hours. After cooling to room temperature, the reaction mixture was passed through a short silica gel column with a layer of active charcoal on the top by using DCM as solvent. The solvents were removed under reduced pressure, and the residue was washed with methanol (100 mL), yielding compound **3** as a white solid (503 mg, 90%).

**$^1$H NMR** (400 MHz, $CDCl_3$, 298 K): δ (ppm) = 8.10-8.05 (m, 2H), 7.73-7367 (m, 4H), 7.65-7.56 (m, 1.8 Hz, 3H), 7.55-7.52 (d, 2H), 7.51-7.48 (m, 2H), 7.37-7.30 (m, 4H), 1.48-1.42 (s, 12H).

**$^{13}$C NMR** (100 MHz, $CDCl_3$, 298 K): δ (ppm) = 142.37, 139.21, 137.33, 137.13, 134.94, 131.46, 130.96, 129.99, 129.81, 128.55, 127.61, 127.10, 127.07, 125.17, 125.14, 84.10, 25.18, 25.13.

**HRMS** (MALDI): Calculated for $C_{32}H_{29}BO_2$: 456.2261; Found 456.2255.

2.7 Synthesis of **P1A2**

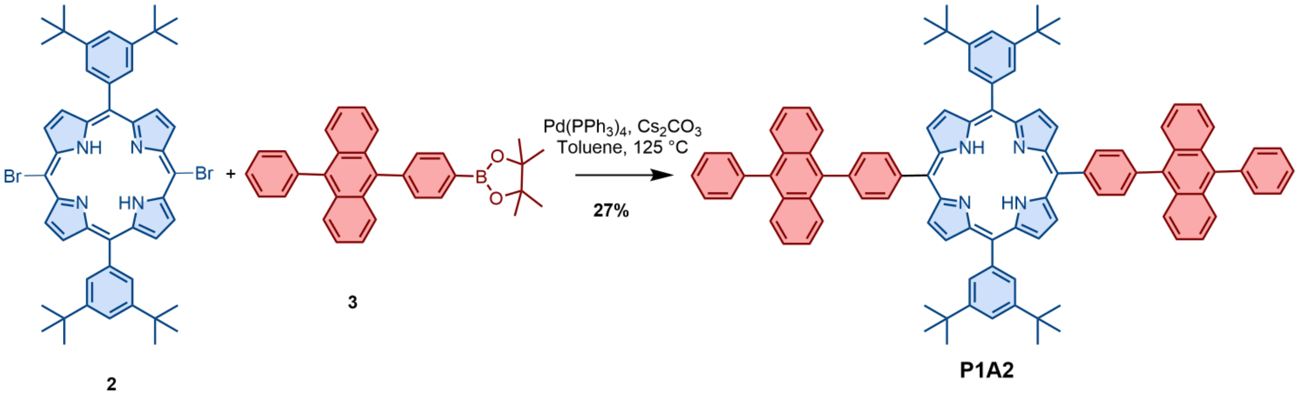


**2**

Compound **2** (100 mg, 0.12 mmol, 1.0 equiv.), compound **3** (162 mg, 0.35 mmol, 3.0 equiv.), $Pd(PPh_3)_4$ (27 mg, 0.023 mmol, 0.2 equiv.), $Cs_2CO_3$ (154 mg, 0.47 mmol, 4.0 equiv.) were mixed in a Schlenk flask under argon atmosphere. Degassed toluene (30 mL) with one drop of water were added to the flask, and the mixture was stirred at 125 °C under argon for two days. After cooling to the room temperature, the crude product was purified by silica gel column with the mixture of DCM/Hexane/Toluene (1:7:1). This yielded compound **P1A2** as a red solid (43 mg, 27%).

**$^1$H NMR** (400 MHz, $CD_2Cl_2$, 298 K): δ (ppm) = 9.03-8.97 (d, $J$ = 4.8 Hz, 4H), 8.97-8.92 (d, $J$ = 4.8 Hz, 4H), 8.50-8.44 (d, $J$ = 8.2 Hz, 4H), 8.11-8.08 (d, $J$ = 1.9 Hz, 4H), 8.06-8.01 (d, $J$ = 8.2 Hz, 4H),7.82-7.79 (t, $J$ =

1.8 Hz, 2H), 7.73-7.68 (m, 4H), 7.67-7.61 (t, *J* = 7.6 Hz, 4H), 7.60-7.53 (m, 6H), 7.40-7.34 (td, *J* = 7.5, 1.3 Hz, 4H), 7.32-7.25 (td, *J* = 7.5, 1.2 Hz, 4H), 7.25-7.18 (m, 4H), 1.50-1.48 (s, 36H), -2.71--2.77 (s, 2H).

**$^{13}$C NMR** spectrum was not able to be obtained due to the very poor solubility of **P1A2**.

**HRMS** (MALDI): Calculated for $C_{100}H_{86}N_4$: 1343.6886; Found 1343.6916.

2.8 Synthesis of **Cu-P1A2**

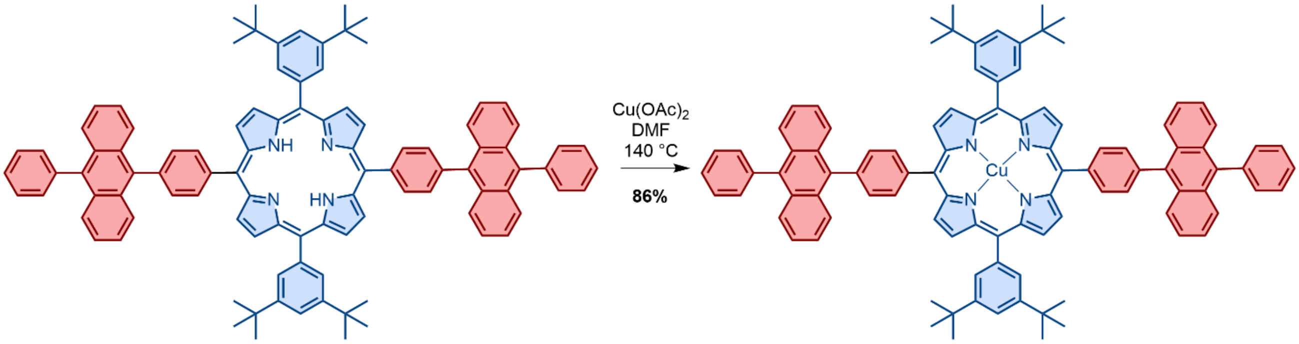


P1A2 Cu-P1A2

Compound **P1A2** (20 mg, 0.149 mmol, 1 equiv.) and $Cu(OAc)_2$ (30 mg, 1.49 mmol, 10 equiv.) were dissolved in DMF (10 mL) in a flask. The mixture was stirred at 140 °C under argon overnight. After cooling to the room temperature, the solvents were removed under reduced pressure. The residue was dissolved in THF, and the mixture was filtered by a short silica gel column. This yielded **Cu-P1A2** as a red solid (18 mg, 86%).

**HRMS** (MALDI): Calculated for $CuC_{100}H_{84}N_4$: 1404.6026; Found 1404.6038.

**HPLC**: See section 3.2.

## 3. HR-MS and HPLC of paramagnetic compounds

Three compounds (**Cu-CPTA-OH**, **Cu[3]CPTA**, and **Cu-P1A2**) are paramagnetic and therefore could not be characterized using $^{1}H$ NMR and $^{13}C$ NMR spectroscopy. To monitor the reactions, step-by-step comparisons of HR-MS data were performed, and HPLC analysis was used to assess the purity of these paramagnetic compounds.

To confirm the purity of **Cu-CPTA-OH** and **Cu[3]CPTA** and rule out the presence of the species lacking one, two, or three copper atoms, the HR-MS with expanded regions are shown in **Figure S3**. The spectra showed no traces of the m/z corresponding to the compounds having lost one, two, or three copper atoms (for **Cu-CPTA-OH**: m/z 3263.4842 [M – Cu + 2H], 3202.5703 [M - 2Cu + 4H], 3141.6563 [M - 3Cu + 6H]; for **Cu[3]CPTA**: m/z 3161.4678 [M – Cu + 2H], 3100.5539 [M - 2Cu + 4H], 3039.6399 [M - 3Cu + 6H]). The purity of the copper-containing compounds was further verified by HPLC analyses in different solvents. (**Section 3.2**) Additionally, our unsuccessful attempts to synthesize the metal-free **[3]CPTA** nanohoop from free base **CPTA-OH** under current conditions rule out the presence of a significant free-base impurity. Based on this collective evidence, we are confident that the compounds are pure at least to the level detectable by HRMS and HPLC (i.e. >98%).

3.1 Comparisons of HR-MS of compounds

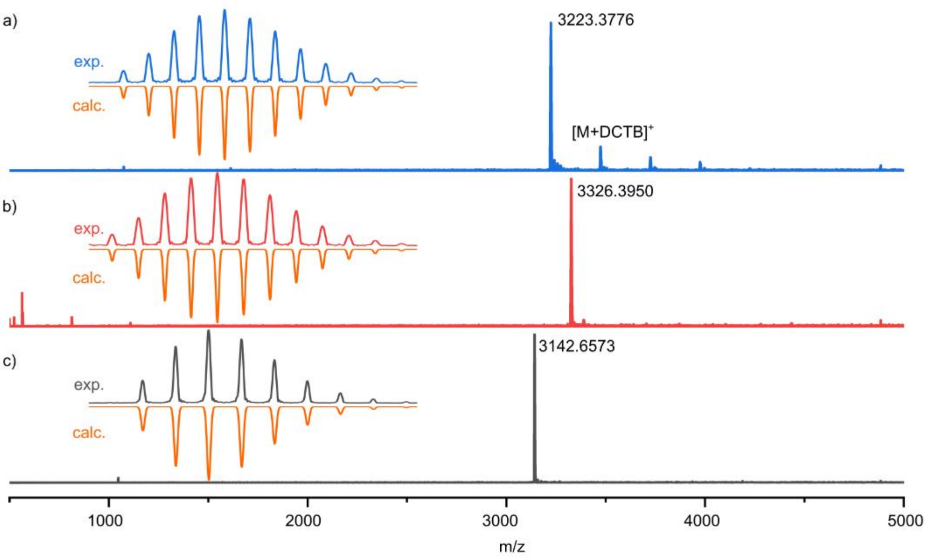


**Figure S1** Comparisons of HRMS of **Cu[3]CPTA** (a), **Cu-CPTA-OH** (b), and **CPTA-OH** (c)

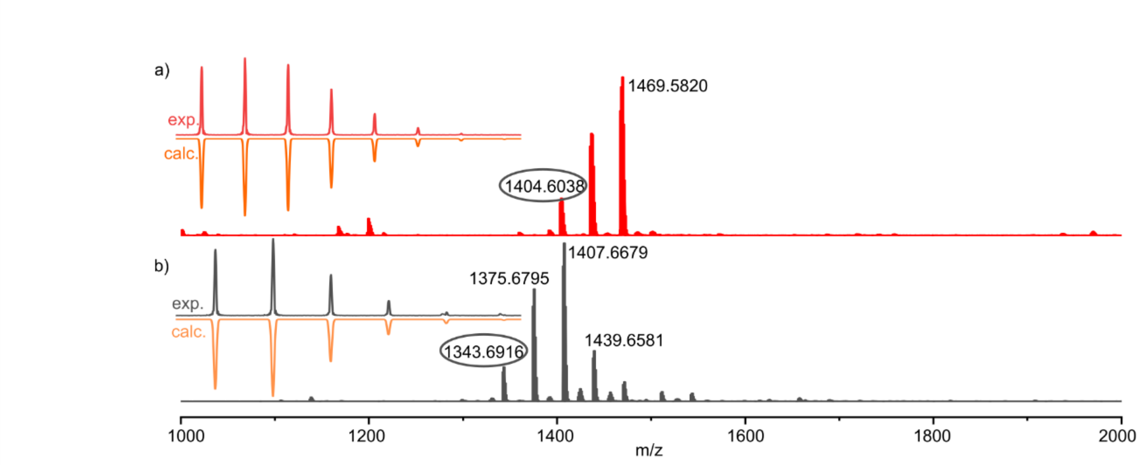


**Figure S2** Comparisons of HRMS of **Cu-P1A2** (a) and **P1A2** (b).

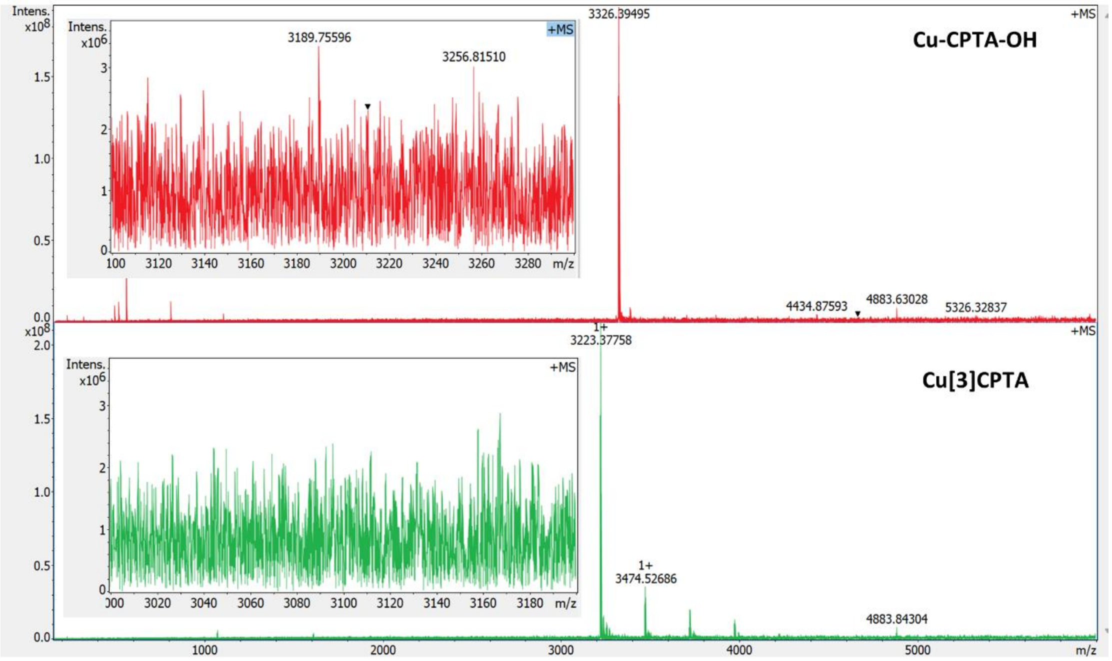


**Figure S3** HR-MS spectra of **Cu-CPTA-OH** (top) and **Cu[3]CPTA** (bottom). The insets show expanded regions for **Cu-CPTA-OH** (m/z 3100–3300) and **Cu[3]CPTA** (m/z 3000–3200).

3.2 HPLC of paramagnetic compounds

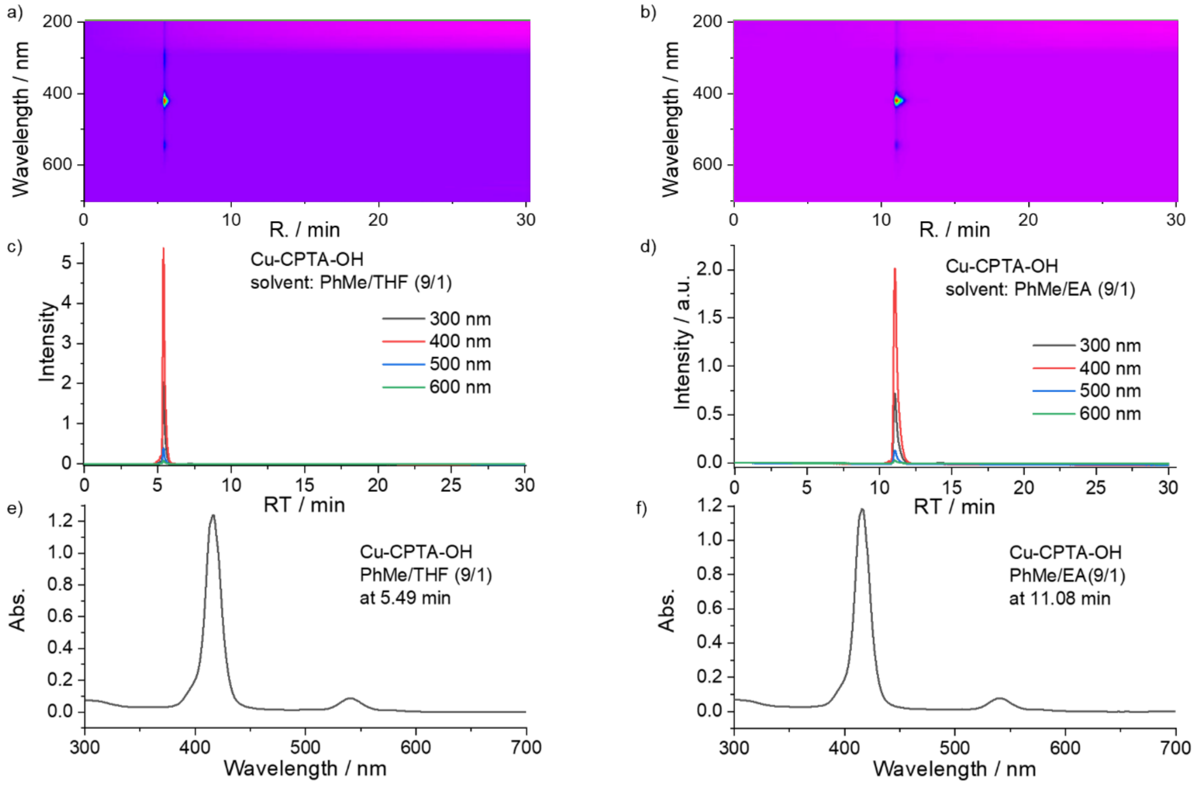


**Figure S4** HPLC characterization of **Cu-CPTA-OH** with mixture toluene and THF (a, c, and e) and mixture of toluene and ethyl acetate (b, d, and f) as mobile phases. HPLC photodiode array mapping (a and b) and chromatogram (c and d) of **Cu-CPTA-OH**. The UV-Vis absorption spectra and mass spectra of sample at 5.49 min (e) in mixture of toluene and THF and 11.9 min (f) in mixture of toluene and ethyl acetate.

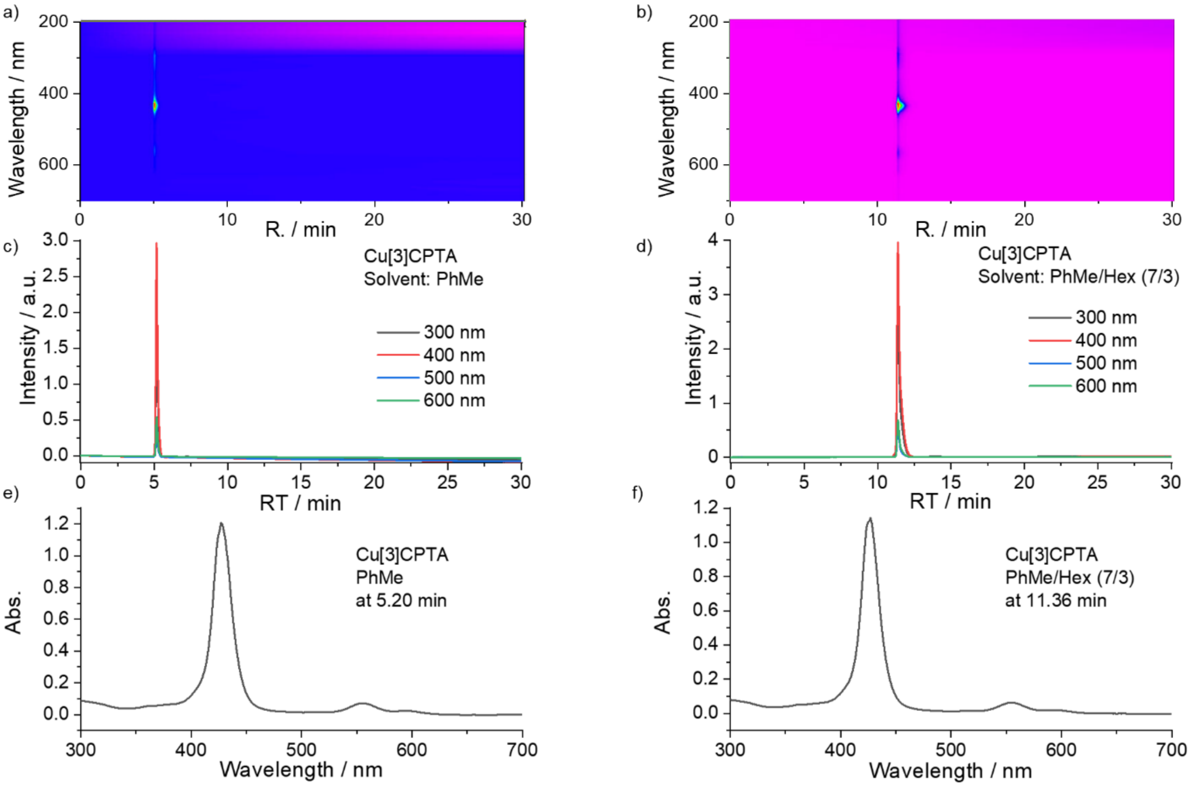


**Figure S5** HPLC characterization of **Cu[3]CPTA** with toluene (a, c, and e) and mixture of toluene and hexane (b, d, and f) as mobile phases. HPLC photodiode array mapping (a and b) and chromatogram (c and d) of **Cu[3]CPTA**. The UV-Vis absorption spectra and mass spectra of sample at 5.20 min (e) in toluene and 11.36 min (f) in mixture of toluene and hexane.

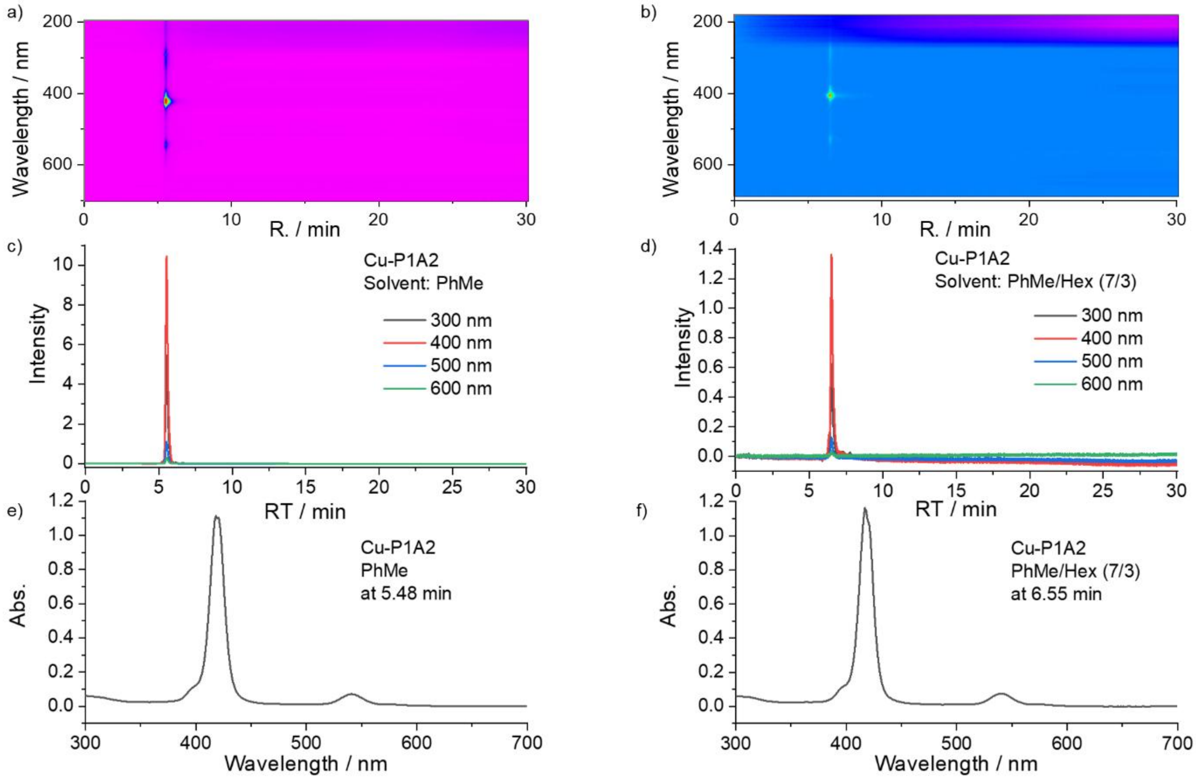


**Figure S6** HPLC characterization of **Cu-P1A2** with toluene (a, c, and e) and mixture of toluene and hexane (b, d, and f) as mobile phases. HPLC photodiode array mapping (a and b) and chromatogram (c and d) of **Cu-P1A2**. The UV-Vis absorption spectra and mass spectra of sample at 5.48 min (e) in toluene and 6.55 min (f) in mixture of toluene and hexane.

## 4. X-Ray crystallography data (Cu[3]CPTA)

Single crystal of **Cu[3]CPTA** was obtained by slow diffusion of hexane into a toluene solution of compound. The crystallographic data of **Cu[3]CPTA** single crystal was collected at 100 K at XALOC beamline at ALBA synchrotron (0.82653 Å).[3] Data were indexed, integrated and scaled using the XDS program.[4] Absorption correction was not applied. The structure was solved by direct methods and subsequently refined by correction of F2 against all reflections, using SHELXT2018 within Olex2 package and WinGX (version 2021.3).[5,6] All nonhydrogen atoms were refined with anisotropic thermal parameters by full-matrix least-squares calculations on F2 using the program SHELXL2018.[7] Thermal motions of some benzene rings and tert-butyl functional groups were restricted by SADI, DELU, EADP and DEFIX. We treated the presence of disordered solvent molecules in the cavities of **Cu[3]CPTA** running solvent mask using Olex2 solvent mask or after location of the cage atoms.[8,9] We counted 994 electrons per unit cell that correspond to 20 toluene molecules. Hydrogens atoms were inserted at calculated positions and constrained with isotropic thermal parameters.

**Table S1** Crystallographic data collection and structure refinement statistics for **Cu[3]CPTA**

| CCDC numbers | **2425217** | |
|---|---|---|
| Bond precision | C-C = 0.0275 | |
| Wavelength | 0.82653 | |
| a/Å | 17.9011(3) | |
| b/Å | 47.6865(8) | |
| c/Å | 36.7042(5) | |
| α/° | 90 | |
| β/° | 90.547(1) | |
| γ/° | 90 | |
| Temperature | 100 K | |
| | **Calculated** | **Reported** |
| Volume/Å$^3$ | 31330.8(9) | 31330.8(9) |
| Space group | $P\ 2_1/n$ | $P\ 1\ 2_1/n\ 1$ |
| Hall group | -P 2yn | -P 2yn |
| Moiety formula | C220 H192 Cu3 N12, 2(C H3) [+ solvent] | C222 H198 Cu3 N12 |
| Sum formula | C222 H198 Cu3 N12 [+ solvent] | C222 H198 Cu3 N12 |
| Mr | 3224.59 | 3224.53 |
| Dx,g cm-3 | 0.684 | 0.684 |
| z | 4 | 4 |
| Mu (mm-1) | 0.350 | 0.351 |
| F000 | 6804.0 | 6804.0 |
| F000’ | 6810.39 | |

| | | |
|---|---|---|
| H, k, lmax | 15, 40, 31 | 15, 40, 31 |
| Nref | 20507 | 20163 |
| Tmin, Tmax | 0.976, 0.979 | |
| Tmin' | 0.976 | |
| Correction method | Not given | |
| Data completeness | 0.983 | |
| Theta(max) | 20.691 | |
| R(reflections) | 0.1296(11230) | |
| wR2(reflections) | 0.3770(20163) | |
| S | 1.318 | |
| Npar | 1856 | |

## 5. Steady optical properties

UV-Vis absorption and photoluminescence spectra of five compounds (**CPTA-OH, Cu-CPTA-OH**, **Cu[3]CPTA**, **P1A2**, and **Cu-P1A2**) were measured in degassed 2-methyltetrahydrofuran (2-MeTHF) at the room temperature with the concentration of 5 × $10^{-7}$ mol/L. Three Cu-containing compounds (**CPTA-OH, Cu-CPTA-OH**, and **Cu-P1A2**) almost don't emit with excitations at 300 nm, 350 nm, 370 nm, 420 nm, and 515 nm.

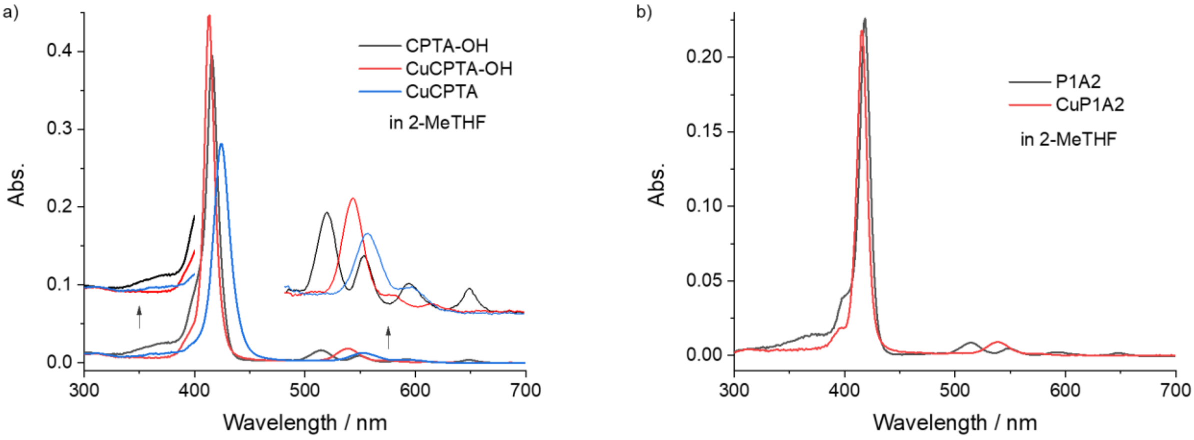


**Figure S7** UV-Vis absorption spectra of five compounds in 2-MeTHF at the concentration of 5 × $10^{-7}$ mol/L (a: **CPTA-OH**, **Cu-CPTA-OH**, and **Cu[3]CPTA**; b: **P1A2** and **CuP1A2**)

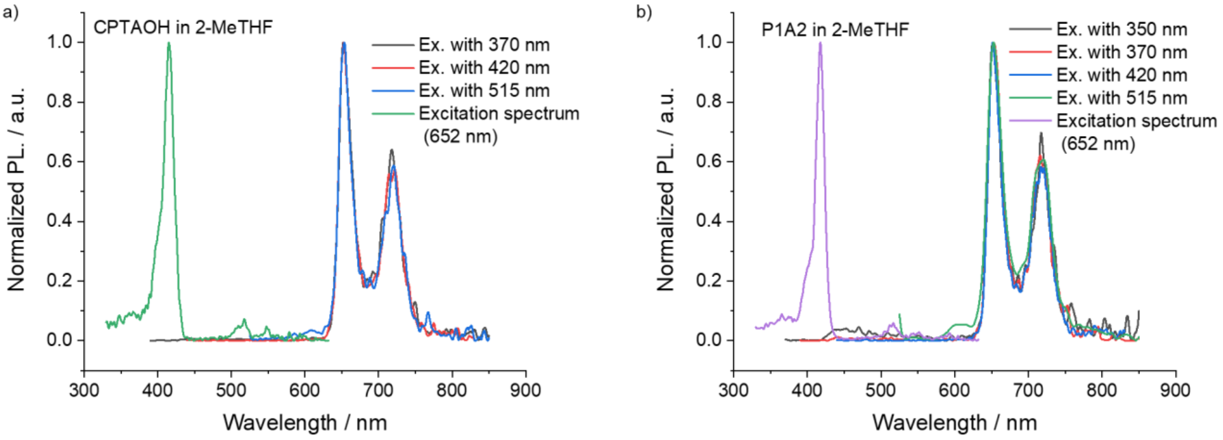


**Figure S8** Normalized photoluminescence and excitation spectra of **CPTA-OH** (a) and **P1A2** (b) in 2-MeTHF at the concentration of 5 × $10^{-7}$ mol/L with different excitations. The excitation spectra were collected by monitoring the emission at 652 nm.

## 6. Transient absorption studies

### 6.1 Ultrafast transient absorption spectroscopy

Femtosecond (fs) transient absorption (TA) spectra were collected using a custom-built setup as previously described.[10] A regenerative Ti:sapphire amplifier (Astrella, Coherent, USA) is used for the fundamental laser, delivering pulses of 5 mJ pulse energy at 1 kHz pulse repetition rate and 800 nm. The fundamental wavelength is frequency doubled in an OPA (TOPAS-C, Lightconversion, Lithuania) for generating the pump at 400 nm of 80 fs pulse duration. As the probe pulse, a white light supercontinuum in the spectral range between 320 and 720 nm is generated by focusing a fraction of the fundamental in a $CaF_2$ plate. The laser power at the sample position is set between 0.4 and 0.5 mW. For TA-spectroscopy, the probe beam is delayed in time with respect to the pump beam by means of an optical delay line and the polarization between probe and pump is set at the magic angle (54.7°). The integrity of the samples was confirmed by recording the UV/Vis absorption spectra (JASCO V-670 spectrometer) at room temperature before and after fs TA measurement.

For data analysis, the fs TA spectra are chirp corrected first. Then the fs TA data were processed and analyzed by a global multi-exponential fit using the Python Package KiMoPack.[11] The data within the temporal window ranging from 0.3 ps to 0.65 ps around time-zero was excluded from the analysis due to contributions from coherent artefacts. Furthermore, a spectral band of 20 nm bandwidth around the pump-wavelength is excluded from the data analysis due to scattering of the pump laser.

For each copper-metalloporphyrin the TA-spectra were measured twice. Exemplary TA spectra and decay associated spectra are shown in Figure 2b in the main text and Figures S9-12 here in the supporting information. TA-features at delay-times shorter than 1 ps are often varying between both measurements due to the influence of the coherent artefact, while TA-features at longer delay times are highly consistent for both measurements. Both mean and standard deviations of the time constants determined by global fitting of the TA raw data are summarized in Table S2.

To collect TA-spectra of the Cu-metalloporphyrin complexes at varying temperature, the sample solutions were measured in a 1 cm pathlength cuvette customized for cryostat experiments. The sample solutions were first purged with argon gas for more than 10 min prior each measurement. Then the cuvette was placed in a temperature-controlled cryostat (Optistat DN, Oxford Instrument) cooled with liquid nitrogen. Temperatures were set (ITC 503S, Oxford Instruments, at 293 K unless otherwise stated) and the real-time temperature inside the cuvette was monitored by a temperature sensor (PT100, SensorShop24, Germany). The corresponding free porphyrin bases **CPTA-OH** and **P1A2** were measured in a standard 1 mm cuvette at room temperature.

**Transient absorption spectroscopy (TAS) of the Cu-porphyrins at 293 K**

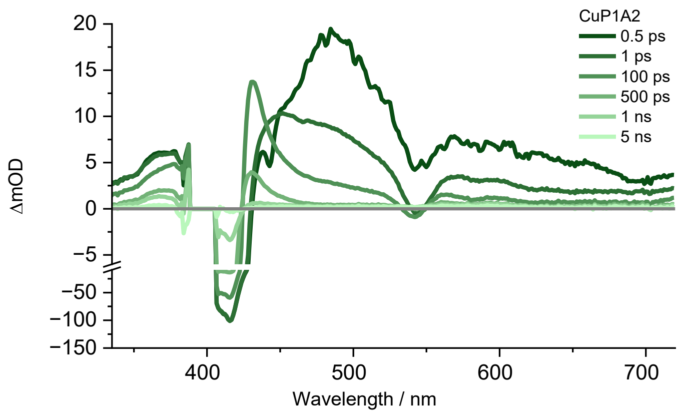


**Figure S9** Transient absorption spectra of **CuP1A2** at different delay times in 2-MeTHF.

The TA-spectra of the linear one-spin reference compound **CuP1A2** closely resembles the TA-spectra of its nanohoop equivalent **Cu[3]CPTA** indicating that the optical excitation is centred on the metalloporphyrin core and does not delocalize over the entire nanohoop (Figure 2b).

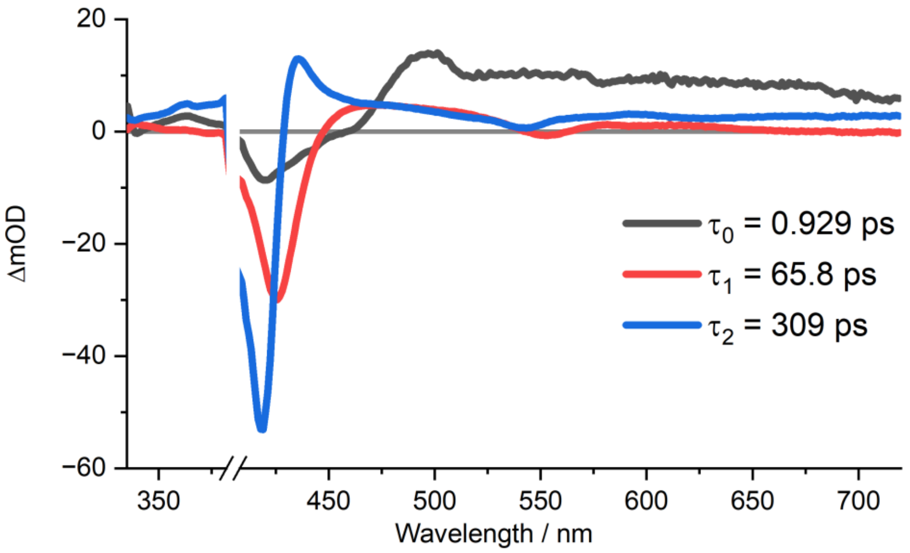


**Figure S10** Decay associated spectra of **Cu[3]CPTA** in 2-MeTHF with the corresponding time constants.

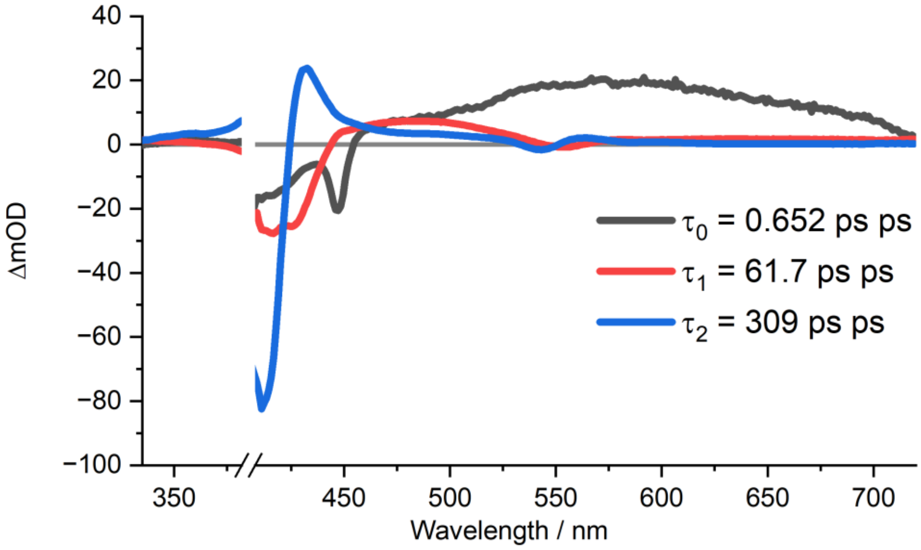


**Figure S11** Decay associated spectra of **Cu-CPTA-OH** in 2-MeTHF with the corresponding time constants.

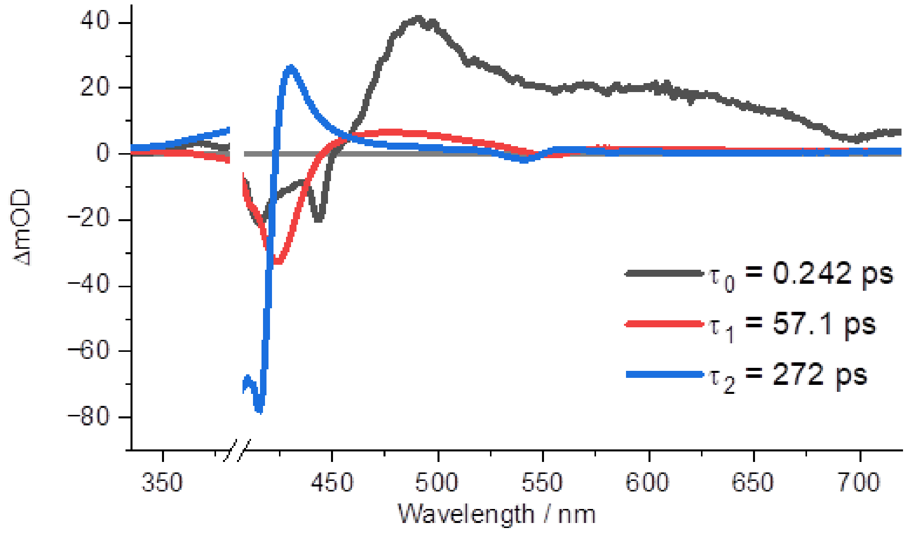


**Figure S12** Decay associated spectra of **CuP1A2** in 2-MeTHF with the corresponding time constants.

**Table S2** Mean values of decay associated time constants according to the scheme in **Figure S13**.

| | $\tau_0$ / ps | $\tau_1$ / ps | $\tau_2$ / ps |
|---|---|---|---|
| **Cu[3]CPTA** | 1.2 ± 0.3 | 65.5 ± 0.3 | 289 ± 20 |
| **Cu-CPTA-OH** | 0.8 ± 0.2 | 61.5 ± 0.2 | 318 ± 10 |
| **CuP1A2** | 0.4 ± 0.2 | 57.6 ± 0.5 | 293 ± 22 |

6.2 Excited states dynamics

In the ground state the $Cu^{2+}$ centre has a $d^9$ electronic configuration, leading to a doublet state due to the unpaired electron in the highest d-orbital in a square planar (SP-4) coordination sphere, the $d_{z^2}$ orbital. Meanwhile the organic framework is in a singlet state. Following Goutermans notation[12] this state is labelled as $^2S_0$ (S = singlet porphyrin, 2 = overall spin, 0 = electronic ground state).

Upon Soret-band excitation at 400 nm a π-π* transition is induced, leading to population of an excited porphyrin centred singlet state $^2S_2$. Fast vibrational cooling and intersystem crossing yield to the population of $^3$(π-π*) states. These ultrafast processes are spectrally characterized by the emergence of the triplet's broad ESA features beginning around 475 nm and extending to the NIR and correlated with the time constant $\tau_0$.[13–15] Ultrafast time-resolved experiments in the gas phase suggest, that the population of the triplet state for copper-metalloporphyrin complexes is enhanced via higher lying spin-allowed (overall multiplicity remains 2) ring to metal charge transfer states.[16–18]

Due to the interaction with the unpaired electron at the $Cu^{2+}$ centre the porphyrin centred $^3$(π-π*) triplet state is split into a doublet- ($^2T_1$) and a quartet state ($^4T_1$).[19] The role of this "tripdoublet" states for the luminescent behaviour and the lifetime of copper-metalloporphyrin triplet states were discussed in the past, but they are not distinguishable in ultrashort TA experiments.[14,17,19,20] Literature reports that an equilibrium between the two triplet state is formed on the hundreds of picosecond scale.

In the triplet state formation of adducts with the solvent, yielding a square-pyramidal (SPY-5) copper complex,[15,18] is promoted due to altered electron density in the excited states.[21] The formation of the SPY-5 exciplexes is diffusion controlled and followed by the population of metal-centred (MC) $^2$(d,d) states due to the promotion of the $d_{x^2-y^2}$ orbital in the SPY-5 coordination sphere, bringing it closer to the $d_{z^2}$ orbital.[15,18,21] The required energy for the d,d transition is likely coming from an energy transfer of the relaxing $^3$(π-π*) state, since the triplet features decrease with increasing delay time as the new narrow ESA feature of the $^2$(d,d) rises with the correlated time constant $\tau_1$. The decay of the long-living $^2$(d,d) state is correlated to the time constant $\tau_2$.

Reports on the excited state dynamics of Cu-Porphyrins suggest the above-described decay path via the excited $^2$(d,d) state solely for O-coordinating solvents.[15,18,22,23] For N-coordinating solvents like pyridine and piperidine the role of adduct formation in the excited state dynamics of Cu-Porphyrins is also discussed.[15,18,23] However, the nitrogen-bonding adduct is suggested to decay via a thermally accessible ring-to-metal charge-transfer state.[18,23] The adduct of N-coordinating solvents is reported to be partially formed already in the ground-state and decays significantly faster compared to the oxygen-bonding adducts.[18] For non-coordinating solvents like toluene two luminescent states from the tripdoublet states $^2T_1$ and $^4T_1$ are reported.[15,18,19,23,24] The corresponding electronic configuration of the copper-metalloporphyrin in SP-4 and SPY-5 coordination sphere are shown in Figures S13 and S14, respectively.

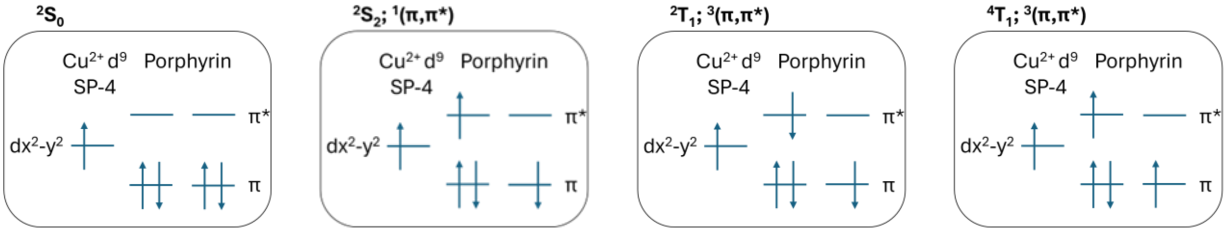


**Figure S13** Participating electronic configurations in the square-planar (SP4) coordination of $Cu^{2+}$.

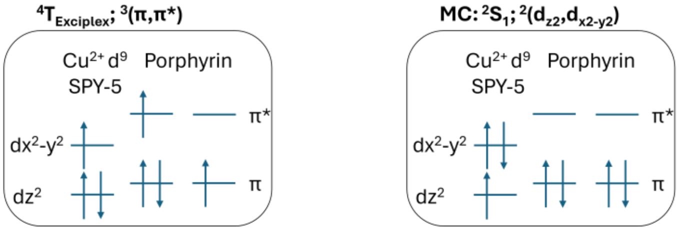


**Figure S14** Participating electronic configurations in the square-pyramidal (SPY-5) coordination of $Cu^{2+}$.

## 6.3 Lower temperature TA-Spectroscopy

Preliminary fs-TA experiments on **Cu-CPTA-OH** and **Cu[3]CPTA** in 2-Me-THF at lower temperature show that the lifetime of both the rise and decay of the $^2$(d,d) state become shorter when lowering the temperature to 225 or 240 K, respectively – indication in respective kinetic traces and time constants. At temperatures close to the freezing point of 2-Me-THF (120 K to 150 K, mp(2-MeTHF) = 137 K) the TA-spectra of **Cu-CPTA-OH** resembles those of the corresponding free porphyrin base, **CPTA-OH,** at room temperature. The increasing time constant for the rise of the $^2$(d,d) state at lower temperatures aligns with the argument that this process is diffusion-controlled,[15,18,21,23,25] since the solvent´s viscosity is inversely proportional to temperature and diffusion coefficient. The purely porphyrin-intrinsic deactivation observed near the freezing point of 2-Me-THF suggests that either the solvent molecules are not available for exciplex formation within the lifetime of the excited metalloporphyrin, or that there are one or more energy barriers within the decay pathway via the $^3$(π,π*) and $^2$(d,d) states. These barriers remain thermodynamically surmountable at 225 K but become insurmountable at 150 K and below.

a)

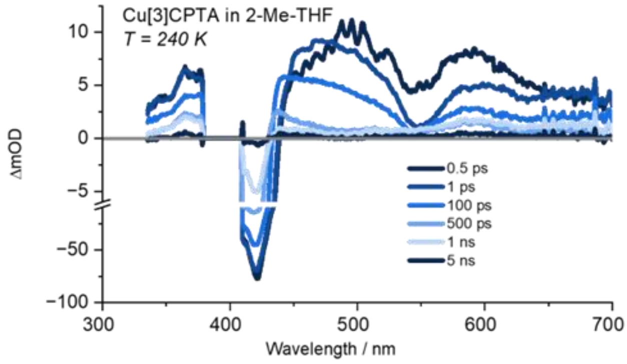


b)

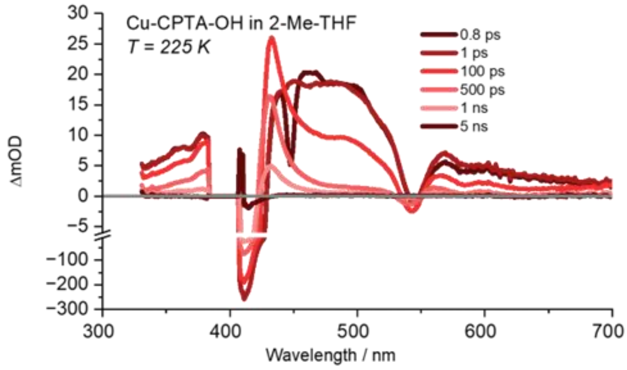


**Figure S15** Key comparative TA-spectra of a) **Cu[3]CPTA** at 240 K and b) **Cu-CPTA-OH** at 225 K.

The spectral features of the two Cu-metalloporphyrin systems basically remain unchanged when lowering the temperature from 293 K (compare Figure 2 in main text) to 240 K or 225 K respectively. However, the

kinetics for the formation and subsequent decay of the $^2$(d,d) state are slowed down – see temporal delayed maximum at lower temperatures for the kinetic traces at 440 nm or 430 nm, respectively in Figure S16 and correlated time constants $\tau_1$ and $\tau_2$ in the DAS in Figure S17.

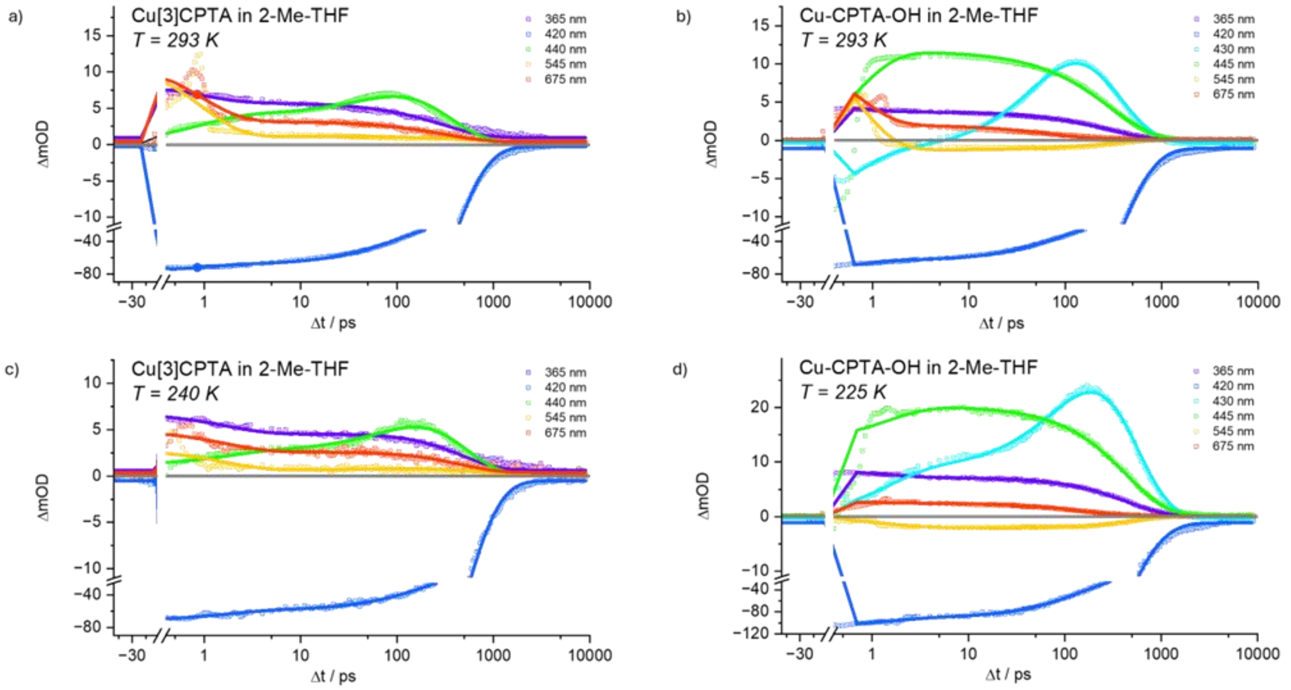


**Figure S16** Comparison of kinetic traces of a) **Cu[3]CPTA** at 293 K, b) **Cu-CPTA-OH** at 293 K, c) **Cu[3]CPTA** at 240 K and d) **Cu-CPTA-OH** at 225 K at selected wavelengths.

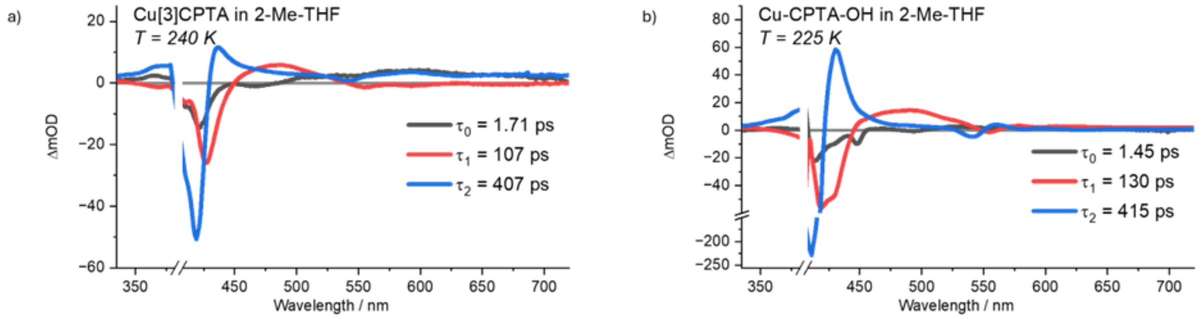


**Figure S17** DAS of a) **Cu[3]CPTA** at 240 K and b) **Cu-CPTA-OH** at 225 K.

The differences in DAS for $\tau_0$ are due to the coherent artefact. The DAS of the two decay components have similar characteristics as compared to 293 K. Namely: DAS($\tau_1$) is associated with the decay of $^3$(π,π*) state and DAS($\tau_2$) with the decay of $^2$(d,d) state.

The increase of the $^3$(π,π*) state lifetime $\tau_1$ by a factor of two may indicate the presence of an energy barrier for exciplex formation or/and for the energy transfer step that yields the $^2$(d,d) state. Alternatively the increasing lifetime $\tau_1$ may be due to the decreasing viscosity of the medium and therefore slower diffusion of the solvent molecules towards the excited $^3$(π,π*), which would be in agreement with the assumption that the exciplex formation is limited by diffusion.[15,18,21,23,25]

The $^{2}$(d,d) lifetime increase indicates the presence of an energy barrier in the decay path of this state. For example, this may be due to the rejection of the ligated solvent, before the metalloporphyrin returns to its electronic ground state.

At lower temperatures (150 K and 120 K) the TA-spectra of the Cu-metalloporphyrin complexes drastically change. After initial relaxation steps in the femto- to picosecond regime **Cu-CPTA-OH** resembles the TA-spectra of the corresponding free porphyrin base **CPTA-OH** at room temperature. Therefore, it seems like the excited porphyrin core is not interacting with the unpaired electron in the metal-centred $d_{z^2}$ orbital in this temperature area anymore. This might be a consequence of a reduced willingness of the solvent molecules for coordination towards the copper centre or missing thermal energy that is required to surmount certain energy barriers in the established decay path at room temperature.

We postulate that the adduct formation of the Cu-metalloporphyrin complexes within the lifetime of the $^{3}(\pi,\pi^{*})$ state is hindered when the solvent is frozen or close to freeze out. The excited **Cu-CPTA-OH** will then slowly decay from the $^{3}(\pi,\pi^{*})$ states ($^{2}T_1$ and $^{4}T_1$ in Figure S13) back to the ground state, as the free porphyrin base **CPTA-OH** does, which could explain the similarity between the TA-spectra shown in Figure S18. The time constant of this $^{3}(\pi,\pi^{*})$ decay ($\tau_2$ in Figure S20) is one magnitude larger than the corresponding time constant of the decay from the metal-centred $^{2}$(d,d) state back to the ground state ($\tau_2$ in Figure S10-S12 and S17).

However further experiments at multiple temperatures points are required to get a full picture of these excited state dynamics. For the present work these preliminary results shall emphasize the impact of the temperature on the kinetics of the excited state relaxation of the presented Cu-metalloporphyrin systems after Soret-band excitation. There appears to be an unsettled thermodynamic threshold for the relaxation via the metal-centred $^{2}$(d,d) state, which is not surmountable anymore in the proximity of the solvents freezing point.

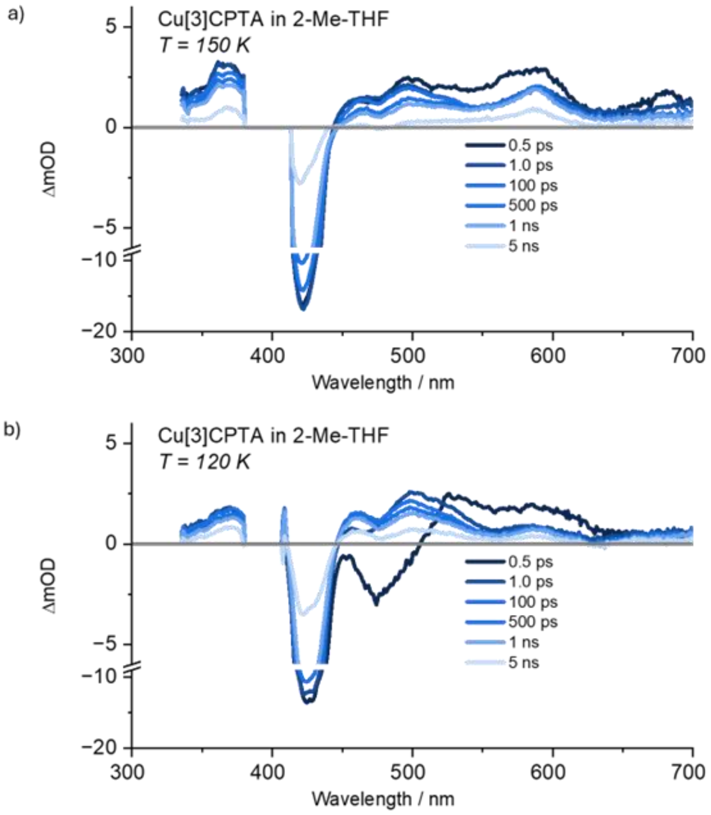


**Figure S18** Comparison of the TA-spectra at selected delay-times of **Cu[3]CPTA** at a) 150 K and b) 120 K.

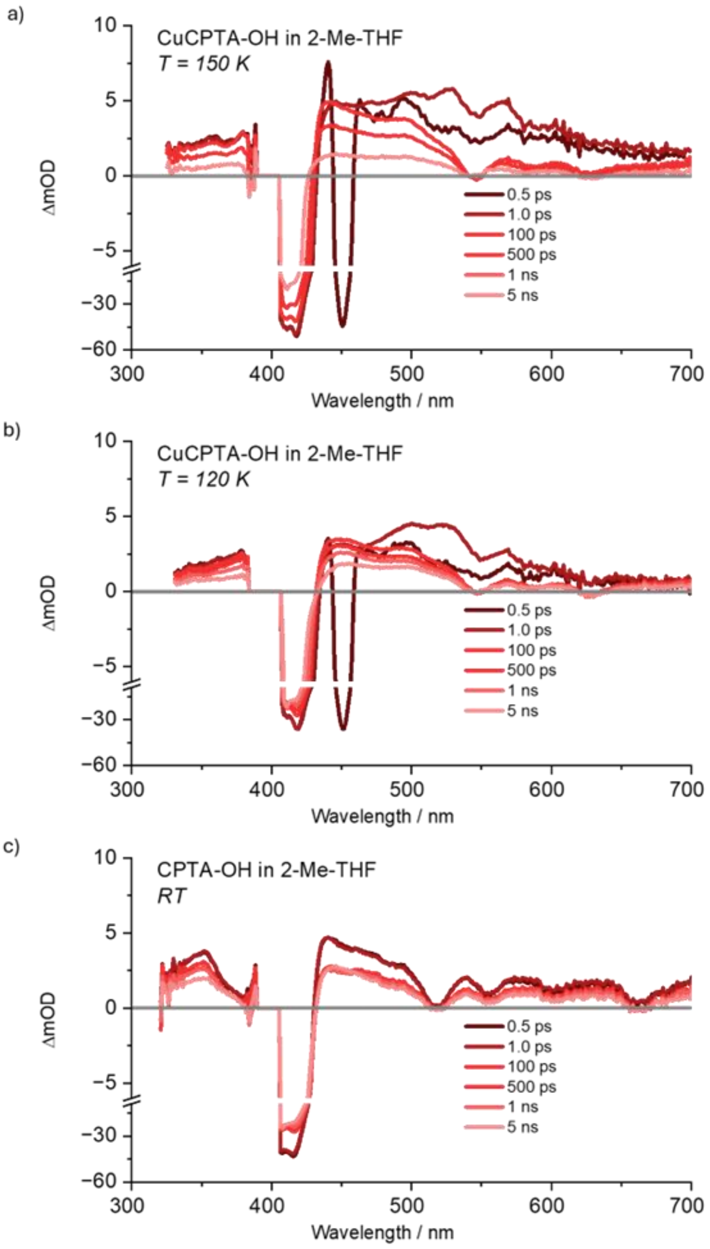


**Figure S19** Comparison of the TA-spectra at selected delay-times of a) **Cu-CPTA-OH** at 150 K and b) 120 K and c) the corresponding metal-free porphyrin base **CPTA-OH** at room temperature.

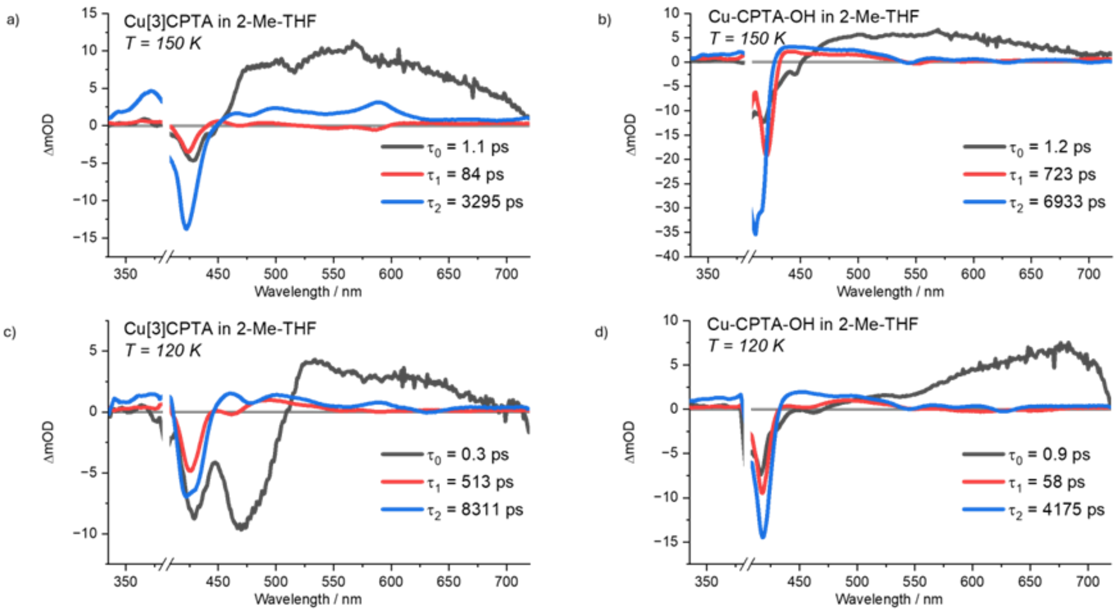


**Figure S20** DAS of a) **Cu[3]CPTA** at 150K, b) **Cu-CPTA-OH** at 150 K, c) **Cu[3]CPTA** at 120K and d) **Cu-CPTA-OH** at 150 K.

## 7. EPR measurements

### 7.1 Materials and methods for EPR part

#### 7.1.1 Sample preparation

For EPR measurements the porphyrin compounds were prepared as 2-MeTHF (passed over a column of activated alumina) solutions to a molecular concentration of roughly 150 µM for continuous-wave and transient EPR measurements and 50–100 µM for pulse EPR measurements. Selected pulse EPR measurements at the Q-band with **Cu[3]CPTA** were performed using toluene-$d_8$ solutions of comparable concentrations. For measurements at the X-band (circa 9.75 GHz) around 60 µL were loaded into 3.8 mm outer-diameter and 3 mm inner-diameter clear-fused quartz tubes; whereas, for measurements at the Q-band (circa 34 GHz) the samples were loaded into 1.6 mm outer-diameter and 1 mm inner-diameter tubes to a filling height of circa 8 mm (roughly 8 µL). For measurements below the solvent freezing-point the samples were rapidly frozen in liquid nitrogen prior to spectrometer insertion—a glassy solid was confirmed by visual inspection.

#### 7.1.2 Continuous-wave EPR

Continuous wave EPR measurements were performed at the X-band ($\nu_{mw}$ ≈ 9.75 GHz) on a Bruker ELEXSYS E580 spectrometer using a Bruker ER-4118X-MD5-W1 dielectric resonator. The experiments were performed at 80 K using liquid nitrogen in combination with a continuous-flow cryostat (CF935, Oxford Instruments) and temperature controller (ITC4, Oxford Instruments). The spectra were acquired with a microwave (mw) power adjusted to below saturation (0.06 mW, corresponding to a mw attenuation of 34 dB) and a modulation amplitude of 0.5 mT, with a modulation frequency of 100 kHz. The data were processed using lab-written Python routines. The spectra were background corrected using a scaled reference measurement on a pure solvent sample. The dc magnetic field was calibrated with a standard carbon fibre sample, with a known *g*-factor ($g$ = 2.002 644),[26] and the frequency corrected to 9.75 GHz.

The numerically integrated cwEPR spectra were subjected to an additional baseline correction with a 5$^{th}$ order polynomial, fitted through the low and high off-resonant field positions, to compensate for minor imperfections in the background correction, *vide supra*.

### 7.1.3 Transient EPR

LASER excitation at 540–592 nm, as indicated, was provided by an Ekspla NT230 series tunable diode-pumped LASER system at a repetition of 50 Hz (pulse duration ≈ 5 ns). Excitation energies were ≈ 2 mJ/pulse incident on the optical window of the cryostat. After the last turning mirror, the light was depolarised using an achromatic depolariser. A Stanford Research Systems digital delay generator (DG645) was used for synchronisation of the LASER system and EPR spectrometer (SpecJet TRIG IN).

Transient EPR experiments were performed at the X-band on a Bruker ELEXSYS E580 spectrometer equipped with a critically coupled Bruker ER-4118X-MD5-W1 dielectric resonator. The experiments were performed at 20 K, using liquid helium in combination with a continuous-flow cryostat (CF935, Oxford Instruments) and temperature controller (ITC4, Oxford Instruments). The data were acquired by direct-detection with the transient recorder (SpecJet-II digitiser) without lock-in amplification using a microwave power of 0.24–1.5 mW (corresponding to an attenuator setting of 28–20 dB). The data were acquired in either transient mode using the IQ-mixer output (VideoAmplifier-II 200 MHz bandwidth) with dcAFC or in cw mode using the diode standard pre-amplifier output with acAFC; for diode detection, the signal was amplified using a Stanford Research Systems low-noise voltage pre-amplifier (SR560) in a 3 kHz–1 MHz bandpass prior to entering the SpecJet Ch2 input. To minimise complicated background signals/artefacts, the trEPR experiments were acquired over several repeats/field sweeps, typically limiting the acquisition of each sweep to 15–20 min. The dataset repeats were acquired using the Python XeprAPI, saving the sweeps individually. The data were processed using lab-written Python routines. During dataset/repeat aggregation, the data were compensated for minor drifts in the mw frequency and interpolated along the field abscissa. The DC offset and LASER backgrounds were removed by two successive 1D baseline-corrections based in the pre-LASER time points as well as the low- and high-field off-resonance transients. The time abscissa was shifted to account for the LASER pulse position and the field abscissa was frequency-corrected to 9.75 GHz and calibrated with a standard carbon fibre sample, with a known *g*-factor ($g$ = 2.002 644).[26]

### 7.1.4 Pulse EPR

Pulse EPR measurements were performed on a Bruker ELEXSYS E580 X/Q-band spectrometer. At the X-band (circa 9.75 GHz), measurements were performed with a Bruker ER-4118X-MD5-W1 dielectric resonator and a 1 kW TWT amplifier (Applied Systems Engineering Inc. 117X) at a temperature of 80 K using liquid nitrogen with a continuous-flow cryostat and temperature controller (ITC4). At the Q-band (circa 33.7 GHz), measurements were performed with a Bruker EN 5107-D2 resonator and either a 150 W TWT amplifier (Applied Systems Engineering Inc. 187Ka) or a 50 W solid-state amplifier (Bruker) at temperature of 15 K or 80 K using liquid helium or nitrogen, respectively, with a continuous-flow cryostat (Oxford Instruments CF935) and a temperature control system (Oxford Instruments ITC 502 or ITC 4). For DEER experiments at the Q-band, pump pulses were formed using an incoherent ELDOR unit (E 580-400U).

Echo-detected field sweeps were recorded using a two-pulse primary echo sequence, typically with pulse lengths of $t_{\pi/2}$ = 16 ns and $t_{\pi}$ = 32 ns, an inter-pulse delay, $\tau$, of 400 ns, a two-step phase cycle and an echo integration window of 160 ns.

Echo decay measurements were performed using the pulse sequence π/2–$\tau$–π–$\tau$–echo, with $t_{\pi/2}$ = 16 ns, $t_{\pi}$ = 32 ns, a two-step phase cycle and an echo integration window of 32 ns. The phase memory times, $T_m$, were determined from stretched exponential fits of the echo decay time traces.

$$I(2\tau) = \exp\left(-\left(\frac{2\tau}{T_m}\right)^{\beta}\right)$$

For decay traces with noticeable ESEEM modulations, the maxima were identified, typically involving the SciPy function argrelmax, and the fit was based on these time points—to maintain a uniform weighting across the entire decay trace, time points located after the modulation pattern had decayed, typically determined by visual inspection of the decay, were sampled at the same rate as the identified maxima.[27,28]

Inversion recovery measurements were performed with an initial inversion pulse of $t_{\pi,\text{ inv.}}$ = 32 ns followed by a two-pulse primary echo sequence for detection with $t_{\pi/2}$ = 16 ns and $t_{\pi}$ = 32 ns, an inter-pulse delay, $\tau$, of 180 ns and a four-step phase cycle ((x)(x)x, with linear-combination coefficients +, −, +, −, cycling the right-most block first, by convention, i.e. [+(+x) +(−x)] for the first pulse and [+(+x) −(−x)] for the second pulse). The spin-lattice relaxation times, $T_1$, were estimated from fits of the inversion recovery traces to a sum of two exponentials model function. The recovery trace could not be adequately modelled using a single exponential term. The longer of the two obtained lifetimes was assumed to correspond to $T_1$.

Four-pulse double electron–electron resonance (DEER) experiments were performed with the observer pulse sequence π/2 –$\tau_1$–π–$\tau_1$–$\tau_2$–π –$\tau_2$–echo applied at the observer frequency, $\nu_{\text{obs}}$, while applying a π pulse at the pump frequency, $\nu_{\text{pump}}$, (incoherent ELDOR unit) at a time, $t_1$ after the first observer π pulse. All pulses used in this work were monochromatic rectangular. During the experiment, the position of the pump π pulse was varied step-wise, with a dwell time Δ$t$, with $t_{10}$ < $\tau$1 and $t_{1\text{end}}$ < $\tau_1$ + $\tau_2$, and the time abscissa in the DEER experiment is obtained as $t_{\text{DEER}} = t_1 - \tau_1$. A two-step phase cycle [+(+$x$) − (−$x$)] was applied on the observer π/2 pulse (($x$)$xx_{\text{p}}x$). Pump pulses were positioned close to the centre of the resonator mode with $\nu_{\text{pump}} = \nu_{\text{obs}} + \Delta\nu$. Parameters for the experimental DEER set-up are presented in Table S3.

The DEER data presented in Figure 3 of the main text for **Cu[3]CPTA** were obtained using a toluene-$d_8$ solution, pumping at the maximum of the echo detected field sweep ($B_0$ = 1178 mT).

**Table S3** DEER set-up and metadata.

| | **Cu-CPTA-OH** (2-MeTHF) | **Cu[3]CPTA** (2-MeTHF) | **Cu[3]CPTA** (toluene-$d_8$) |
|---|---|---|---|
| $t_{\frac{\pi}{2}\text{obs}}$ / ns | 24 | 32 | 32 |
| $t_{\pi\text{obs}}$ / ns | 24 | 32 | 32 |
| $t_{\pi\text{pump}}$ / ns | 12 | 12 | 12 |
| $\Delta\nu$ / MHz | -80 | -100 | -80 |
| $\Delta t$ / ns | 2 | 4 | 4 |
| $\tau_1$ / ns | 300 | 400 | 400$^{\ddagger}$ |
| $\tau_2$ / $\mu$s | 0.8 | 1.5 | 1.5 |
| SRT / ms | 4 | 4 | 4 |
| $t_{gate}$ / ns | 24 | 32 | 32 |
| ESEEM averaging | - | - | 8 steps of 16 ns |

| | | | |
|---|---|---|---|
| averages† | 40960 | 46080 | 14400[a], 19200[b], 33600[c] |

†total number of echos recorded, in each case accounting for shots-per-point, sweeps, phase cycling, and ESEEM averaging (if used), and ‡starting $\tau_1$ for ESEEM modulation averaging.

[a]1178 mT, [b]1167 mT, and [c]1146 mT.

7.1.5 Rabi nutations

Rabi nutation experiments were performed using the pulse sequence $t_p$ − $T$ − $t_{obs}$ − $\tau$ − $2t_{obs}$ − $\tau$ − echo, where the flip angle of the first mw pulse was varied during the experiment by increasing the pulse length, $t_p$, in steps of 2 or 4 ns, starting at 0 ns, and recording the integrated intensity of the full echo. The inter-pulse delay period, $T$, was chosen to be longer than $5T_m$ and the detection sequence used $t_{obs}$ = 10 ns and $\tau$ = 250 ns. A two-step phase cycle, [+(+$x$) − (−$x$)], was applied on the first observer pulse ($x$($x$)$x$). All mw pulses were created using the stripline pulse forming unit and the mw pulse amplitudes were varied by changing the high-power attenuator (HPA) setting, in 3 dB steps. The HPA values (in dB) were converted to relative pulse amplitudes, $10^{\Delta_{HPA}/20\ \mathrm{dB}}$, in each case using the highest attenuator setting as the reference power (relative pulse amplitude equal to unity).

7.2 Additional cwEPR data

The cwEPR spectrum of **CuP1A2** was simulated in MATLAB using functions from the EasySpin package.[29] (Figure S21) The spectrum was simulated as an axial spin-1/2 system with one axial copper hyperfine (natural abundance) and four equivalent axial $^{14}$N hyperfine couplings, all $\underline{g}$- and hyperfine interaction matrices were assumed to be collinear. The simulation was performed using full matrix diagonalisation with an enlarged orientational grid (Opt.GridSize = [61, 4]) and considered all transitions (Opt.Threshold = 0).

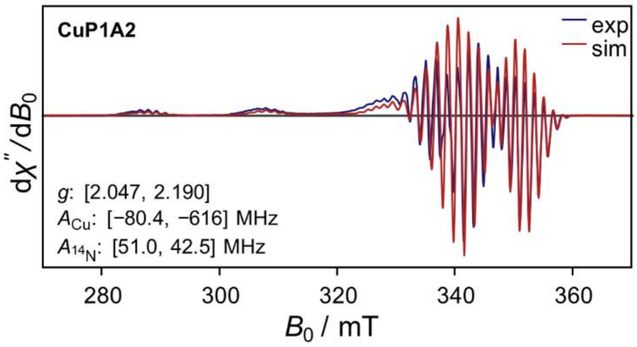


**Figure S21** Continuous-wave EPR spectrum of **CuP1A2** alongside a numerical simulation, $\underline{g}$- and hyperfine interaction matrices are annotated and additional details are discussed in the text.

7.3 Additional trEPR data

The light-induced polarization was not observed for the systems investigated within the experimental time resolution at 80 K, consistent with previous investigations of a free base porphyrin dyad, unlike more organic complexes.[30,31]

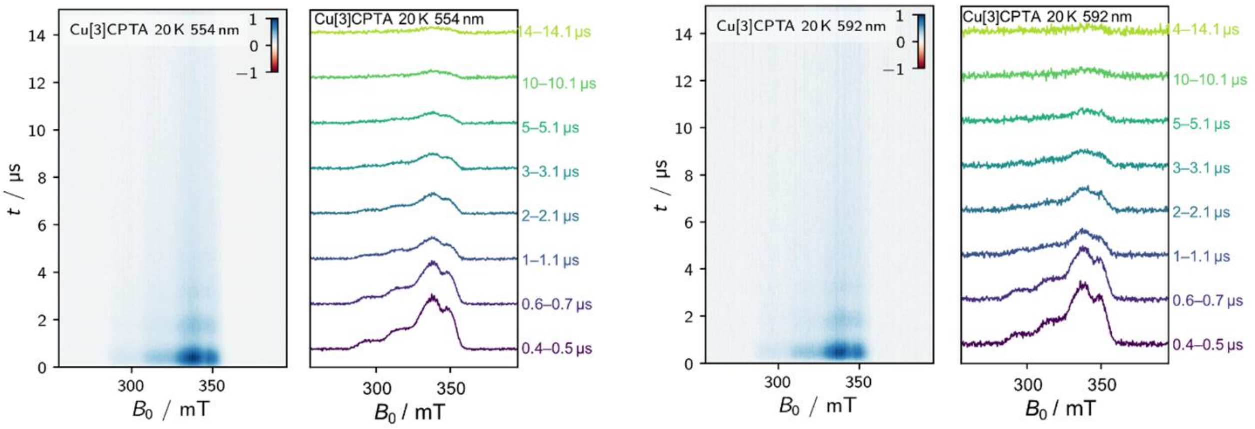


**Figure S22** Transient EPR spectra for **Cu[3]CPTA** measured at 20 K with excitation at the indicated wavelengths. The 2D datasets are presented as contour projections and integrated time-slice spectra, delays as indicated.

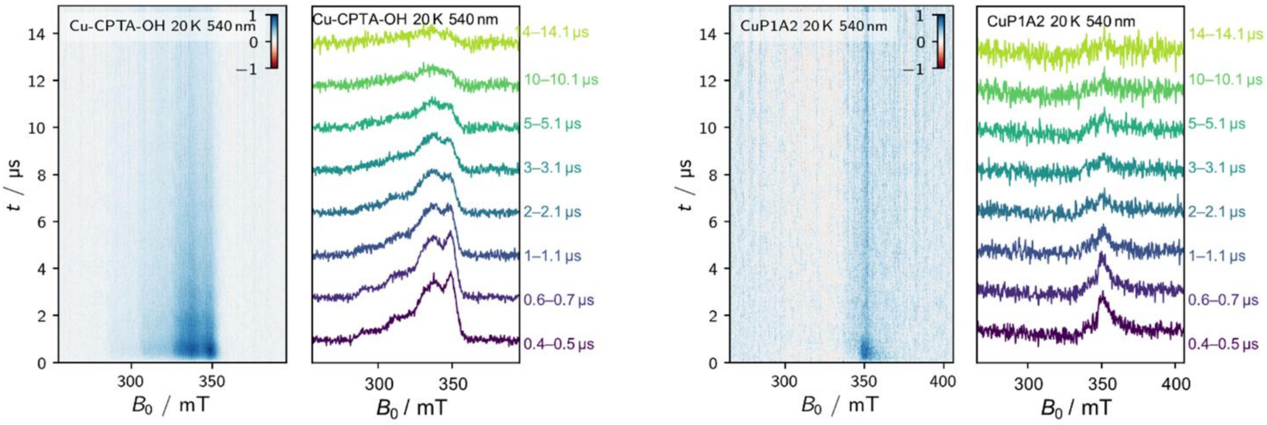


**Figure S23** Transient EPR spectra for **Cu-CPTA-OH** (*left*) and **CuP1A2** (*right*) measured at 20 K with excitation at the indicated wavelengths. The 2D datasets are presented as contour projections and integrated time-slice spectra, delays as indicated.

In general, the decay of the trEPR signal is characterized by a complex dependence on the $\vec{B}_1$ field strength, the phase-memory time, and the spin–lattice relaxation lifetime of the copper center.[32–35] Transients integrated over a narrow magnetic-field range in the $g_\perp$ region are presented in Figure S24. The oscillations present in the decays, most clearly observed for **Cu[3]CPTA**, are transient nutations or Torrey oscillations arising from a precession of the magnetization about $\vec{B}_1$. In the limit limit $v_1^2 T_1 T_2 \ll 1$ (overdamping), which is not satisfied for the data presented herein, the transient signal would decay as an exponential with a lifetime corresponding to the $T_1$ of the ground-state copper centre. Whereas in the limit $v_1 \gg \frac{1}{T_2}$ and $T_1 \gg T_2$ (underdamping) the transient signal takes the form of a damped zeroth-order Bessel function of the first kind, $J_0(v_1 t)$, where the exponential damping has a lifetime corresponding to $2T_2$. In a previous report on ground-state light-induced polarisation in copper porphyrins,[36] where the authors state that the data were measured in the overdamping regime*, it was found that the transient signal did not decay with a single $T_1$ but as a sum of two exponentials, which the authors speculate to arise from either

two types of complexes in the sample, with different relaxation properties, or competition in the rates of electronic transitions and spin–lattice relaxation. (**Note that in this referenced work, as far as we understand, the authors incorrectly refer to the low $\vec{B}_1$ limiting case as underdamping 'The kinetics were found to be independent of microwave power (0.08–2.8 mW) and did not exhibit any transient nutations. Thus, the experiments are carried out at under-damping [sic] conditions'*)

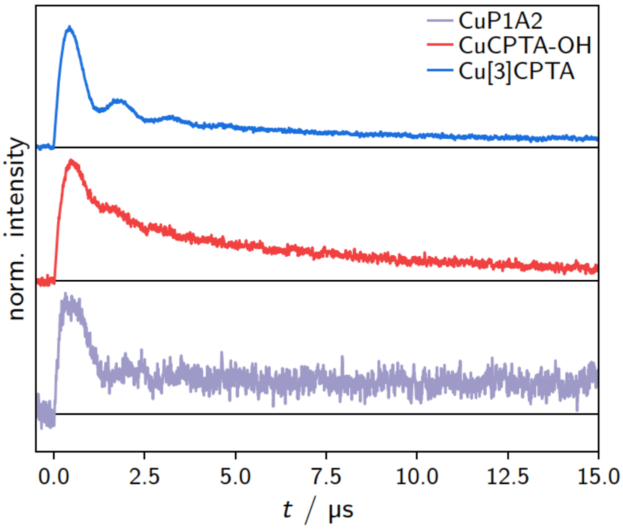


**Figure S24** Comparison of the trEPR transients for **CuP1A2**, **Cu-CPTA-OH**, **Cu[3]CPTA**, 349–351 mT. The transient decay is characterized by a complex dependence on the $\vec{B}_1$ field strength, the phase-memory time, and the spin–lattice relaxation lifetime of the ground-state copper center.

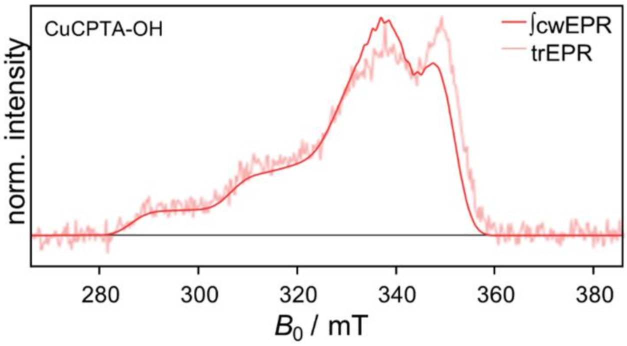


**Figure S25** Comparison of the numerically integrated field-modulated cwEPR spectrum and trEPR (400–500 ns) spectrum of **Cu-CPTA-OH**.

7.4 Relaxation time measurements

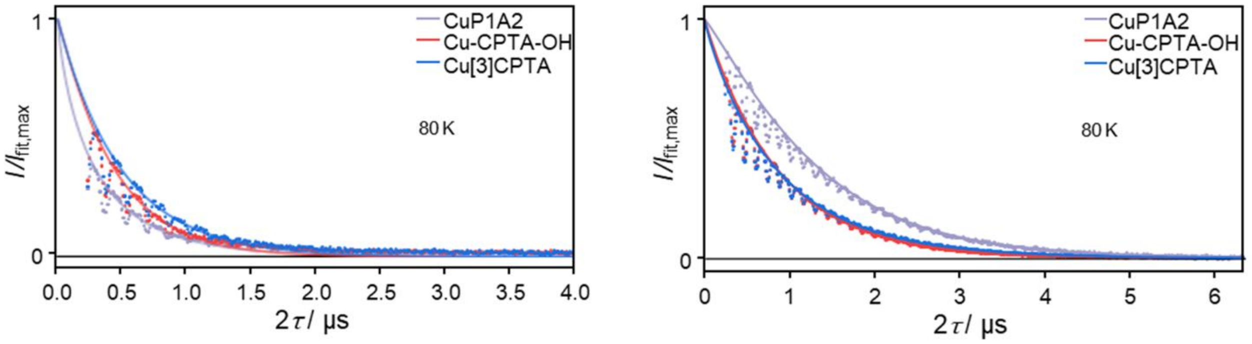


**Figure S26** Two-pulse echo decay traces (marker) and stretched exponential fits (line) for **CuP1A2**, **Cu-CPTA-OH**, and **Cu[3]CPTA**, prepared as 2-MeTHF solutions, recorded at the X-band ($T$ = 80 K). The experiments were performed at magnetic field positions corresponding to $z$ (≈290 mT) and $xy$ (≈339 mT) orientations, *left* and *right*, respectively. The traces were normalised to the intensity at $\tau$ = 0 ns extrapolated from the fits.

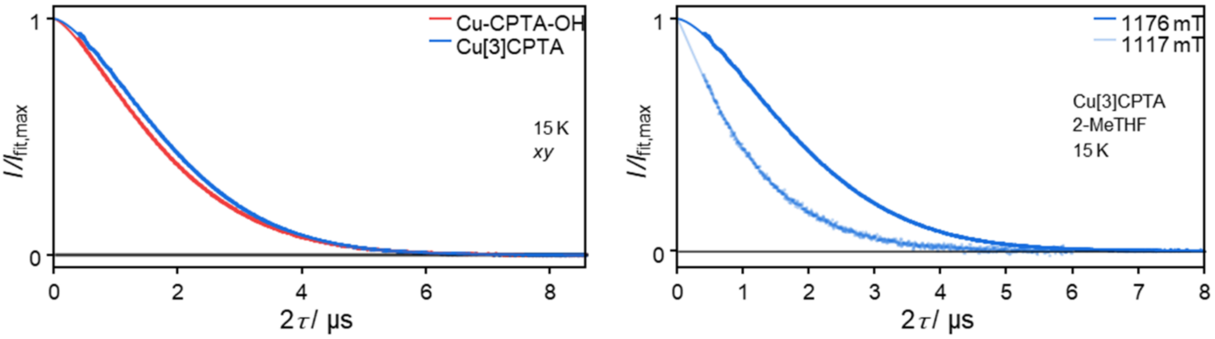


**Figure S27** Two-pulse echo decay traces (marker) and stretched exponential fits (line) for **Cu-CPTA-OH** and **Cu[3]CPTA**, prepared as 2-MeTHF solutions, recorded at the Q-band (T = 15 K). Left shows a comparison of the two macrocycles measured at magnetic field positions corresponding to the $xy$ (≈ 1176 mT) orientation. Right compares the magnetic field/orientational dependence of the two-pulse echo decays for **Cu[3]CPTA** recorded at the $z$ and $xy$ orientation, 1117 and 1176 mT, respectively. The traces were normalised to the intensity at τ = 0 ns, extrapolated from the fits.

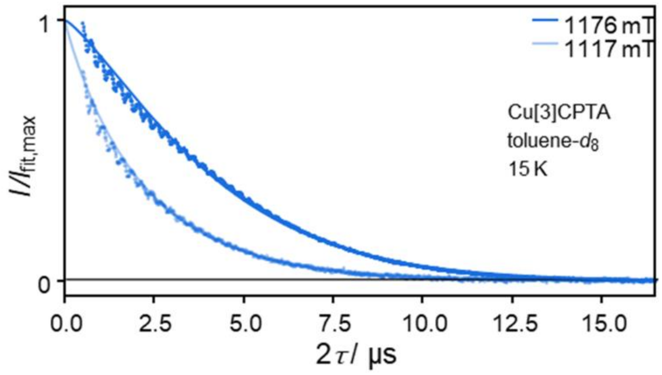


**Figure S28** Two-pulse echo decay traces (marker) and stretched exponential fits (line) for **Cu[3]CPTA**, prepared as a toluene-$d_8$ solution, recorded at the Q-band ($T$ = 15 K). The traces were normalised to the

intensity at $\tau$ = 0 ns extrapolated from the fits.

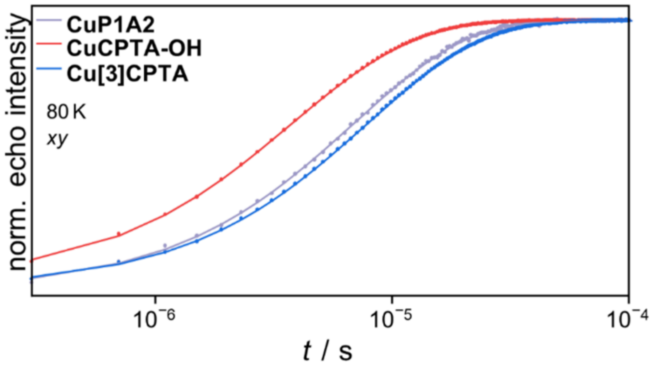


**Figure S29** Inversion recovery traces (marker) and sum of two exponential fits (line) for **CuP1A2**, **Cu-CPTA-OH**, and **Cu[3]CPTA**, prepared as 2-MeTHF solutions, recorded at the X-band ($T$ = 80 K). The recovery traces are normalised according to the mean intensity after complete recovery. The measurements were performed at a field position corresponding to the maximum in the two-pulse echo-detected field-swept spectrum (≈339 mT).

**Table S4** Relaxation parameters for measurements at the X-band (9.75 GHz), 80 K, parameters correspond to the fits presented in Figures S26 and S29.

| | $B_0$ / mT | $T_m$ / $\mu$s | $\beta$ | $T_1$ / s |
|---|---|---|---|---|
| **CuP1A2** | 290 mT | 0.29 | 0.78 | - |
| | 339 mT | 1.35 | 1.14 | $8.45 \times 10^{-6}$ |
| **Cu-CPTA-OH** | 291 mT | 0.44 | 1.04 | - |
| | 338 mT | 0.87 | 1.0 | $6.1 \times 10^{-6}$ |
| **Cu[3]CPTA** | 291 mT | 0.49 | 0.99 | - |
| | 339 mT | 0.86 | 0.93 | $1.14 \times 10^{-5}$ |

**Table S5** Relaxation parameters for measurements at the Q-band (33.73 GHz), 15 K, parameters correspond to the fits presented in Figures S27 and S28.

| | $B_0$ / mT | $T_m$ / $\mu$s | $\beta$ |
|---|---|---|---|
| **Cu-CPTA-OH** | 1174 mT | 2.05 | 1.42 |
| **Cu[3]CPTA** (2-MeTHF) | 1116 mT | 1.19 | 1.14 |
| | 1176 mT | 2.22 | 1.55 |
| **Cu[3]CPTA** (toluene-$d_8$) | 1116 mT | 2.18 | 0.95 |
| | 1177 mT | 4.42 | 1.29 |

7.5 Additional DEER data

In Figures S31, S32, S34 and S36, the distance abscissae have been corrected to account for the copper *g*-factor.

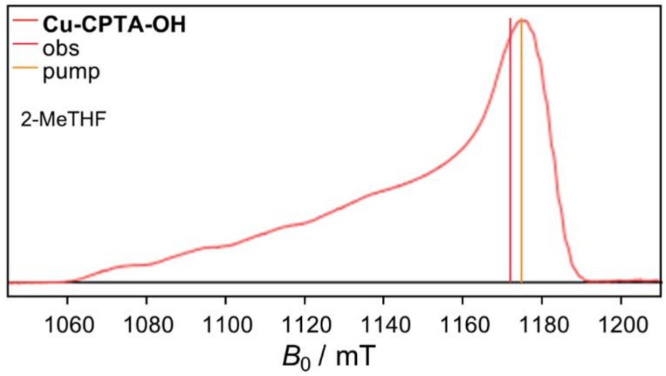


**Figure S30** Two-pulse echo detected field swept spectrum of **Cu-CPTA-OH**, recorded at the Q-band and 15 K. The positions of the pump and observer pulses, in relation to the EPR spectrum, used in the DEER measurements presented in Figure S32 are indicated.

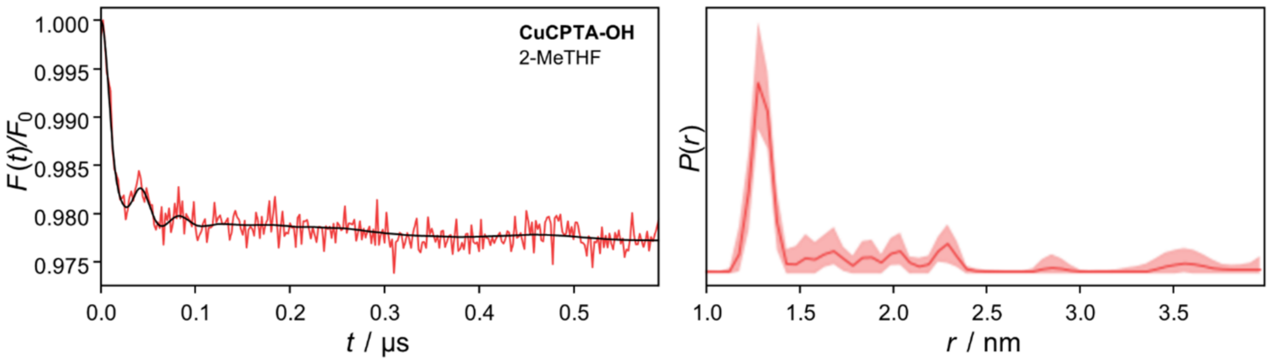


**Figure S31** Additional DEER data for **Cu-CPTA-OH**. Experimental DEER form factor (red) and fit (black) obtained from DeerAnalysis, *left* and associated distance distributions, *right*.

The DEER data were additionally analysed with DeerLab fitting the distance distribution and background simultaneously using Tikhonov regularisation and compactness regularisation.[37,38] The Akaike information criterion (AIC) and the information complexity criterion (ICC) were used to select regularisation parameters for Tikhonov and compactness regularisations, respectively.

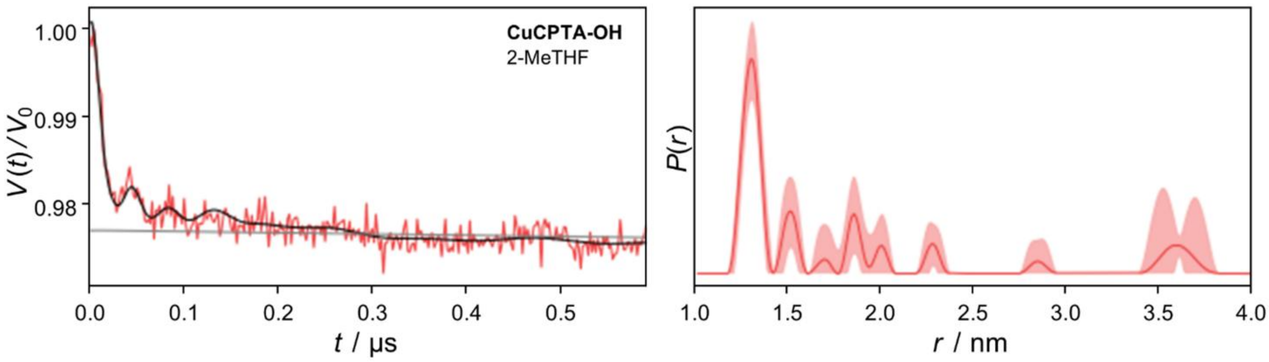


**Figure S32** Additional DEER data for **Cu-CPTA-OH**. Raw DEER time trace (red), foreground fit (black), and background fit (grey), *left* and associated distance distributions, *right*, the 95 % confidence interval is

indicated by the shaded region. $t_0$ = 146.9 ns, $\lambda$ = 0.024, SNR = 20.7, smoothness parameter = 0.006, compactness parameter = 0.

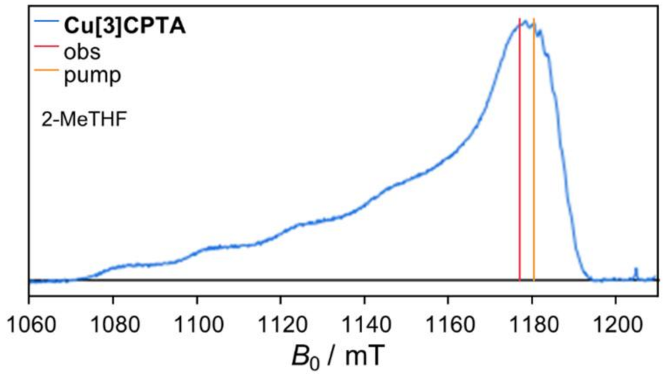


**Figure S33** Two-pulse echo detected field swept spectrum of **Cu[3]CPTA** as a 2-MeTHF solution, recorded at the Q-band and 15 K. The positions of the pump and observer pulses, in relation to the EPR spectrum, used in the DEER measurements presented in Figure S34 are indicated.

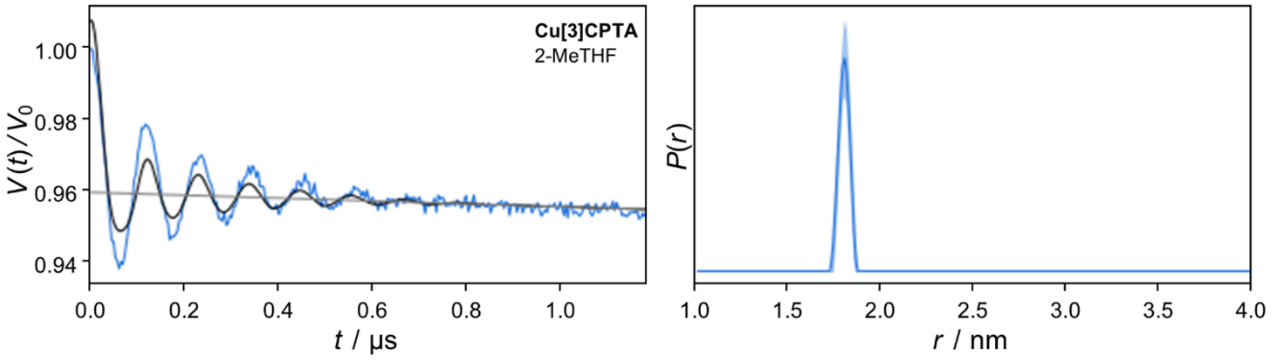


**Figure S34** Additional DEER data for **Cu[3]CPTA**. Raw DEER time trace (blue), foreground fit (black), and background fit (grey), *left* and associated distance distributions, *right*, the 95 % confidence interval is indicated by the shaded region. The DEER data were analysed with DeerLab fitting the distance distribution and background simultaneously using Tikhonov regularisation and compactness regularisation. The Akaike information criterion (AIC) and the information complexity criterion (ICC) were used to select regularisation parameters for Tikhonov and compactness regularisations, respectively. $t_0$ = 112.3 ns, $\lambda$ = 0.048, SNR = 37.0, smoothness parameter = 0.004, compactness parameter = 0.449.

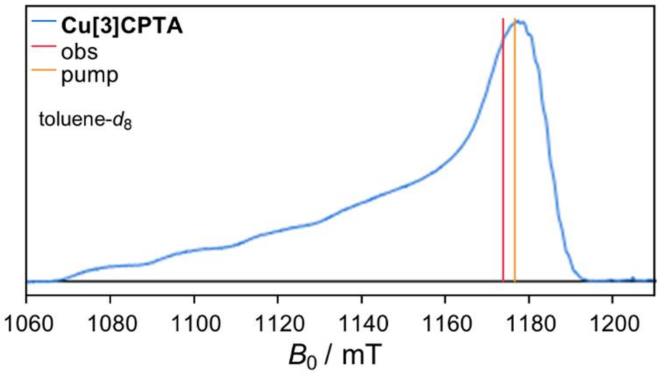


**Figure S35** Two-pulse echo detected field swept spectrum of **Cu[3]CPTA** as a toluene-$d_8$ solution, recorded at the Q-band and 15 K. The positions of the pump and observer pulses, in relation to the EPR spectrum, used in the DEER measurements presented in Figure S36 are indicated.

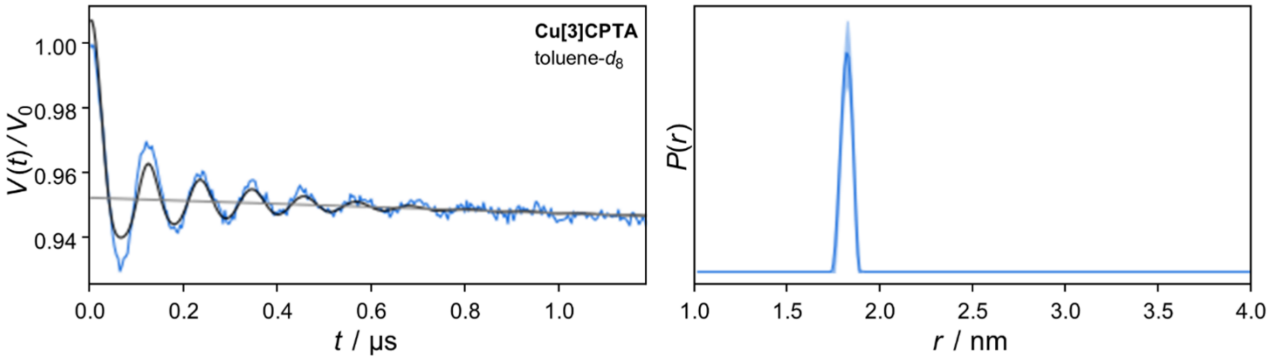


**Figure S36** Additional DEER data for **Cu[3]CPTA**. Raw DEER time trace (blue), foreground fit (black), and background fit (grey), *left* and associated distance distributions, *right*, the 95 % confidence interval is indicated by the shaded region. The DEER data were analysed with DeerLab fitting the distance distribution and background simultaneously using Tikhonov regularisation and compactness regularisation. The Akaike information criterion (AIC) and the information complexity criterion (ICC) were used to select regularisation parameters for Tikhonov and compactness regularisations, respectively. $t_0$ = 109.9 ns, $\lambda$ = 0.055, SNR = 39.8, smoothness parameter = 0.003, compactness parameter = 0.606.

7.5.1 **Cu[3]CPTA** DEER with orientation selection

In an attempt to further characterise the geometry of the **Cu[3]CPTA** system, DEER traces were recorded at additional magnetic-field positions. In this section the raw DEER data measured the field position corresponding to pumping on the maximum of the two-pulse field sweep (1178 mT) is the same as that presented in the main text Figure 3 and Figure S36.

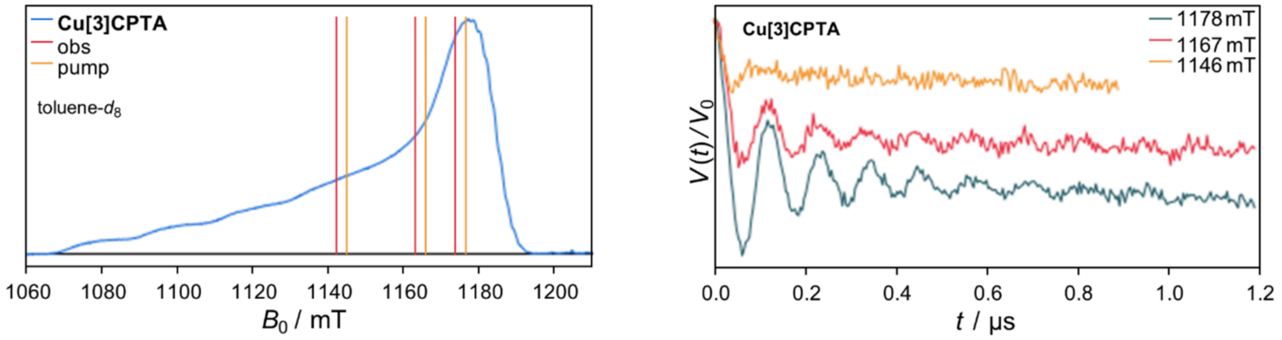


**Figure S37** Two-pulse echo detected field sweep recorded at the pump mw frequency, *left*, the orange vertical lines indicate the three field positions for the DEER and the red vertical lines indicate the observer position (80 MHz). The DEER form factors for the three field positions are depicted, as indicated, *right*.

The resulting datasets were analysed taking orientation selection into account using the PDSFit package.[39] Based on existing knowledge of the crystal structure and basic geometry, the data were first analysed using a constrained model. Within this model, the copper centres were assumed to be positioned at the vertices of an equilateral triangle and the angles were defined according to Figure S38.

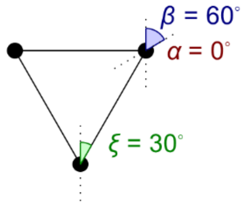


**Figure S38** In the constrained model, the copper centres are positioned at the vertices of an equilateral triangle. Assuming that the principle axis vector of each copper centre associated with the $g_z$ eigenvalue is collinear with the relevant circle radius, it is expected that, assuming axial spin centres, the dipolar angles are as follows: $\xi$ = 30°, $\alpha$ = 0°, and $\beta$ = 60° (by the inversion symmetry of $\underline{g}$). The $\xi$, $\alpha$, and $\beta$ angles are defined in line with reference.[39]

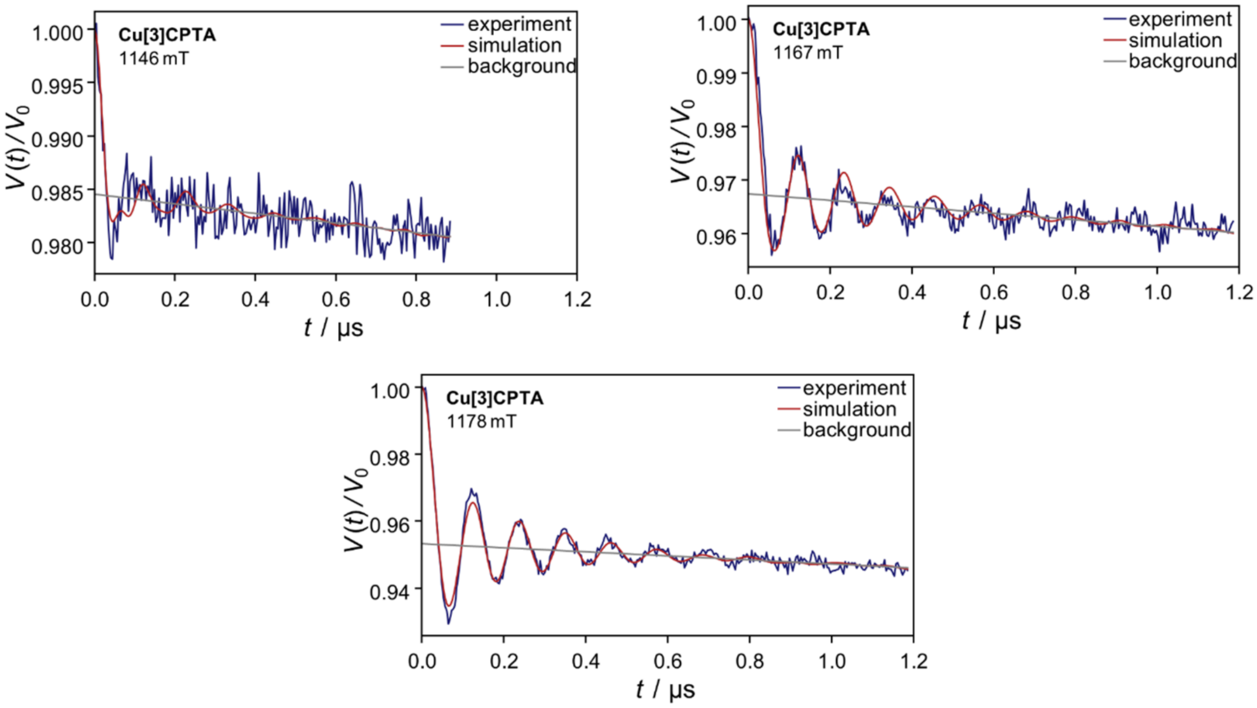


**Figure 39** Experimental DEER traces and best-fit simulations from a constrained axial model for the three magnetic field positions indicated in Figure S37. The ⟨$\xi$⟩, ⟨$\alpha$⟩, and ⟨$\beta$⟩ were used as defined in Figure S38 and the best-fit orientational parameters are presented in Table S6.

An additional analysis was performed where the angles $\xi$, $\alpha$, and $\beta$ were allowed to vary to explore how these parameters influenced the analysis. It is clear that the obtained distance distribution is highly conserved but the fitted angles are poorly defined for the measured datasets.

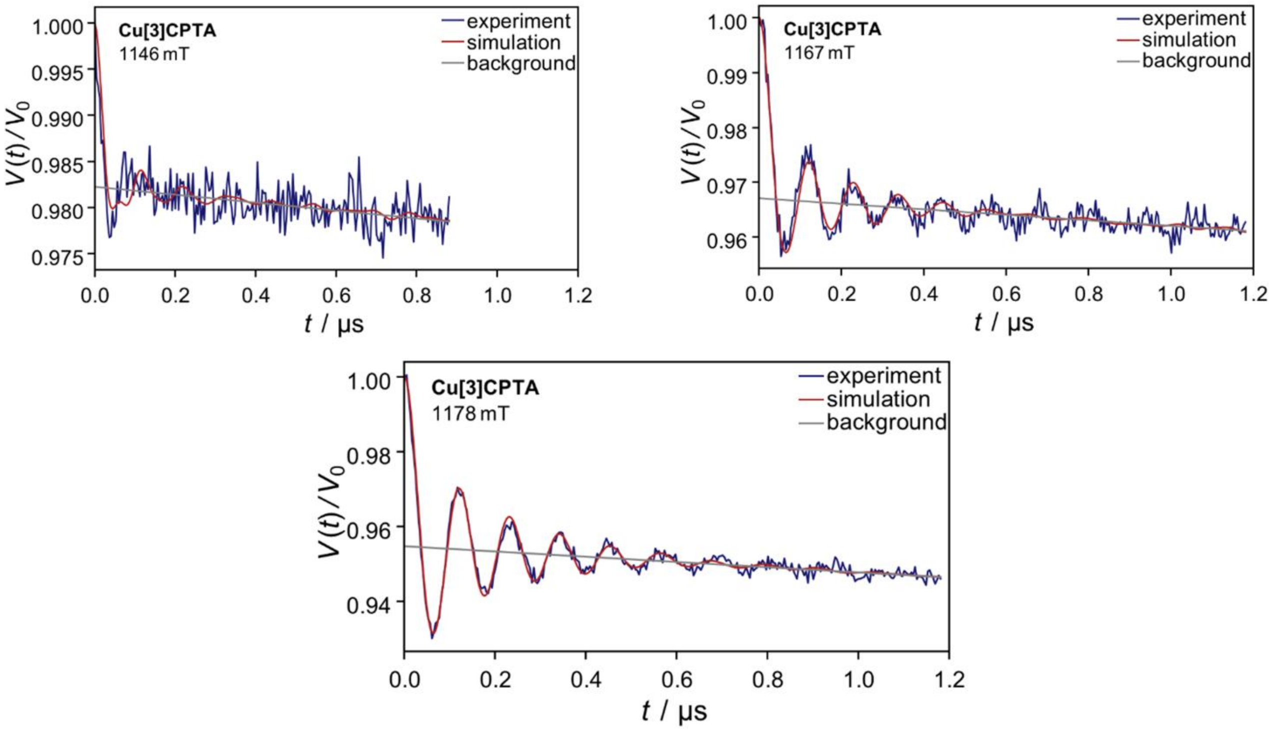


**Figure S40** Experimental DEER traces and best-fit simulations from an unconstrained axial model for the three magnetic field positions indicated in Figure S37. The best-fit orientational parameters are presented in Table S6.

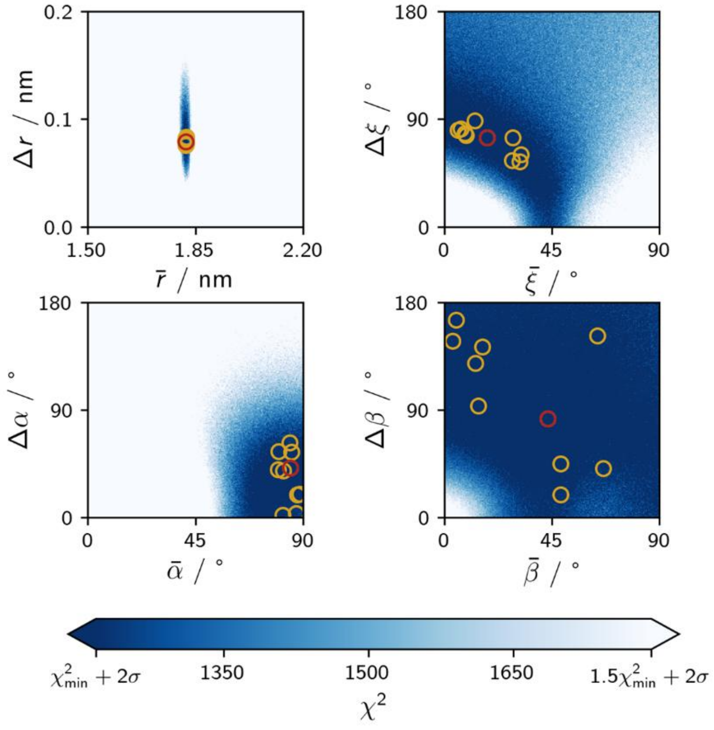


**Figure S41** Fit error surfaces of the distance and angle distributions for the simulations presented in Figure S40, defined by a mean value and a width. The best-fit for 10 independent fittings are indicated by the circular markers, the overall best-fit simulation parameters, determined by $X^2$, are indicated by the red

circle marker. In the colourmap the darker the blue the better the fit, as quantified by $X^2$.

**Table S6** Best fit distance and angle orientational parameters obtained from the orientation selective DEER experiments and simulations presented in Figures S39 and S40. ⊤ this parameter was fixed during the fitting and is defined in Figure S38.

| | axial unconstrained | axial constrained |
|---|---|---|
| $\langle r \rangle$ / nm | 1.82 | 1.83 |
| $\Delta r$ / nm | 0.08 | 0.06 |
| $\langle \xi \rangle$ / ° | 17.9 | 30⊤ |
| $\Delta \xi$ / ° | 74.3 | 0.4 |
| $\langle \alpha \rangle$/ ° | 84.6 | 0⊤ |
| $\Delta \alpha$/ ° | 41.5 | 174.2 |
| $\langle \beta \rangle$/ ° | 43.4 | 60⊤ |
| $\Delta \beta$/ ° | 82.7 | 173.2 |

7.6 Rabi nutations

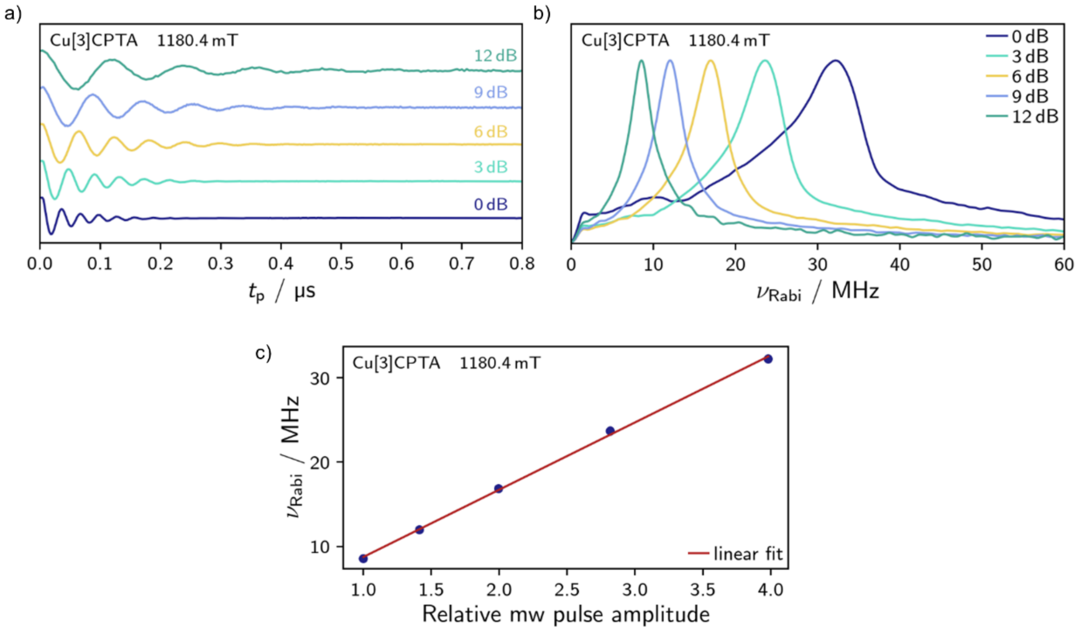


**Figure 42** Power-dependent nutation time traces (a) and magnitude FFT spectra (b) for the Rabi oscillation measurements for **Cu[3]CPTA**, acquired at a magnetic-field position in the $g_{\perp}$ region at 80 K. (c) plot of the Rabi frequency as a function of the relative $\vec{B}_1$ microwave field strength.

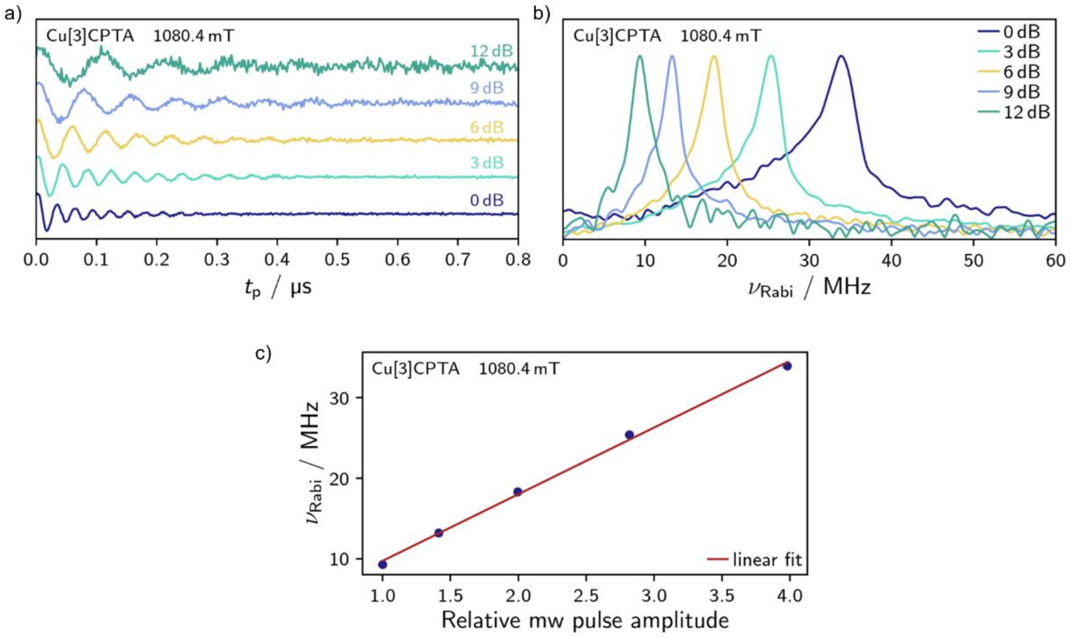


**Figure 43** Power-dependent nutation time traces (a) and magnitude FFT spectra (b) for the Rabi oscillation measurements for **Cu[3]CPTA**, acquired at a magnetic-field position in the $g_{//}$ region at 80 K. (c) plot of the Rabi frequency as a function of the relative $\vec{B}_1$ microwave field strength.

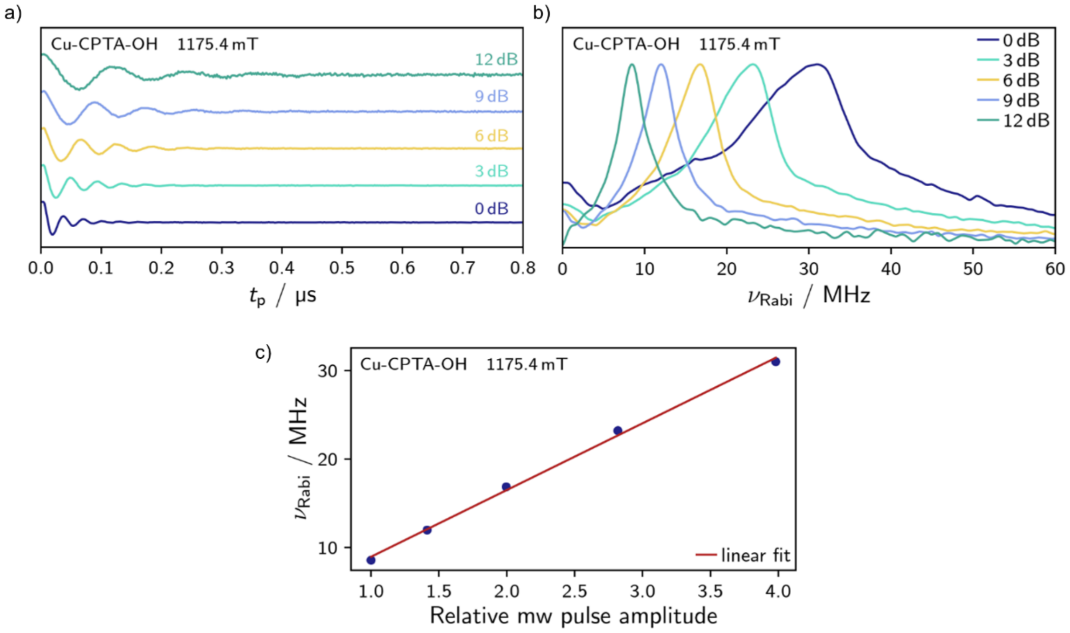


**Figure 44** Power-dependent nutation time traces (a) and magnitude FFT spectra (b) for the Rabi oscillation measurements for **Cu-CPTA-OH**, acquired at a magnetic-field position in the $g_{\perp}$ region at 80 K. (c) plot of the Rabi frequency as a function of the relative $\vec{B}_1$ microwave field strength.

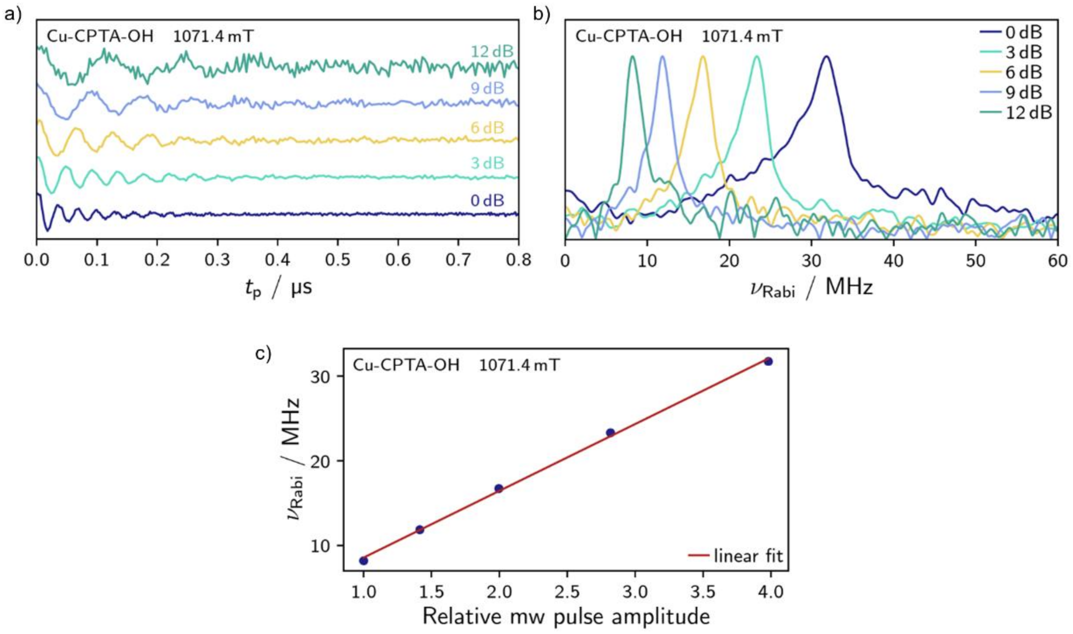


**Figure 45** Power-dependent nutation time traces (a) and magnitude FFT spectra (b) for the Rabi oscillation measurements for **Cu-CPTA-OH**, acquired at a magnetic-field position in the $g_{//}$ region at 80 K. (c) plot of the Rabi frequency as a function of the relative $\vec{B}_1$ microwave field strength.

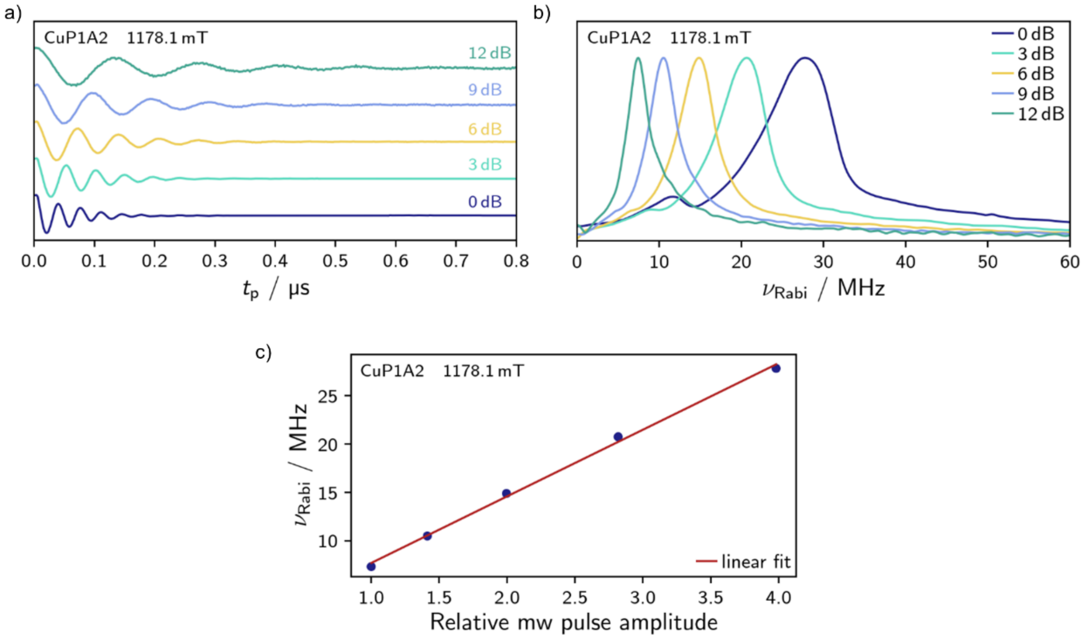


**Figure 46** Power-dependent nutation time traces (a) and magnitude FFT spectra (b) for the Rabi oscillation measurements for **CuP1A2**, acquired at a magnetic-field position in the $g_{\perp}$ region at 80 K. (c) plot of the Rabi frequency as a function of the relative $\vec{B}_1$ microwave field strength.

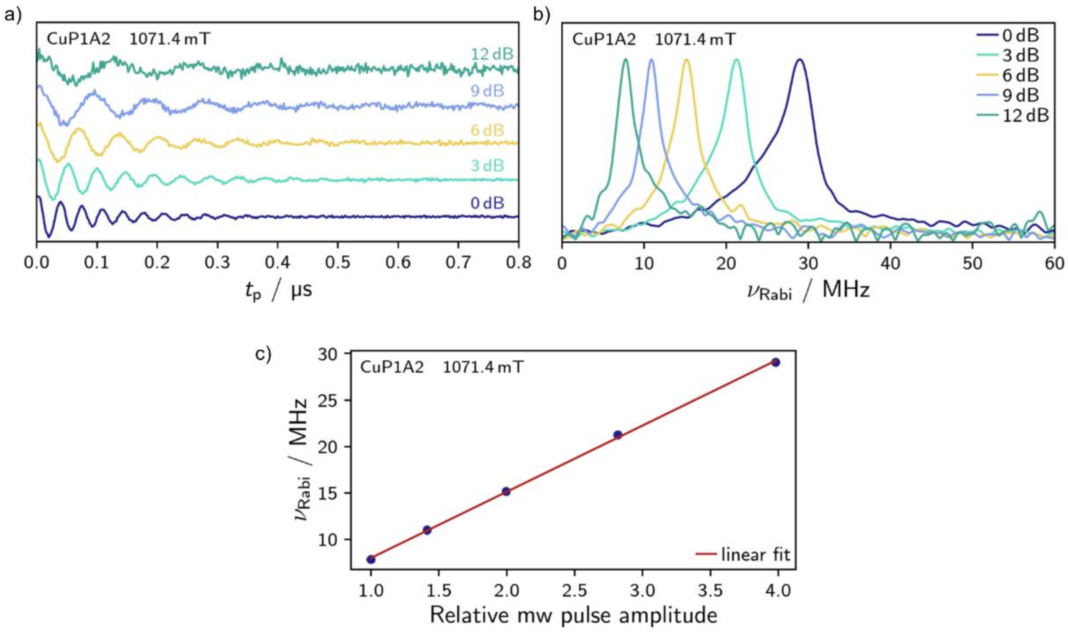


**Figure 47** Power-dependent nutation time traces (a) and magnitude FFT spectra (b) for the Rabi oscillation measurements for **CuP1A2**, acquired at a magnetic-field position in the $g_{//}$ region at 80 K. (c) plot of the Rabi frequency as a function of the relative $\vec{B}_1$ microwave field strength.

## 8. DFT calculations

The computations were performed using the Gaussian quantum chemistry software suite (Gaussian® 16)[40] on the high-performance computing system 'bwForCluster Chemistry' provided by the state of Baden-Württemberg. The B97D3 functional combined with the 6-31G(d,p) basis set was employed, incorporating empirical dispersion corrections via Grimme's GD3BJ model with Becke-Johnson damping.[41–46] The molecular geometry of CPTA-OH was optimized without constraints, and the resulting structures were validated through vibrational frequency analysis.

Due to the flexibility from non-aromatic anthracene units before aromatization, the macrocycle acquired a tighter conformation in which there are multiple interactions between tButyl groups from adjacent porphyrins and neighboring porphyrin cores**.** (Figure S48)

The data of geometry optimized structure can be downloaded under the following link: https://zenodo.org/records/15365308.

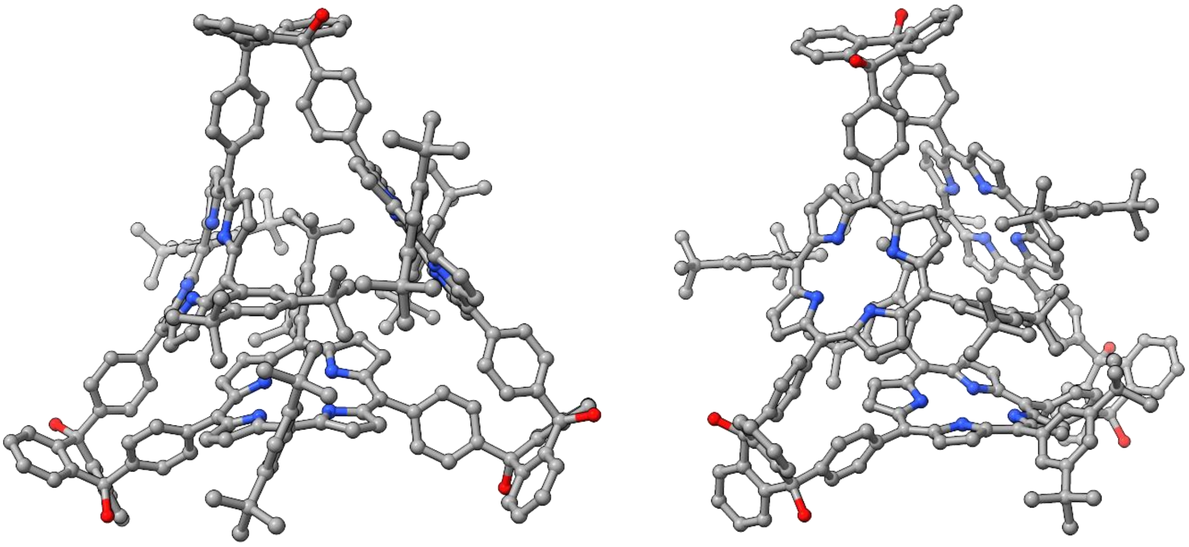

**Figure S48** Optimized structure of CPTA-OH. Hydrogen atoms are omitted for clarity.

## 9. Supplementary NMR and mass spectra

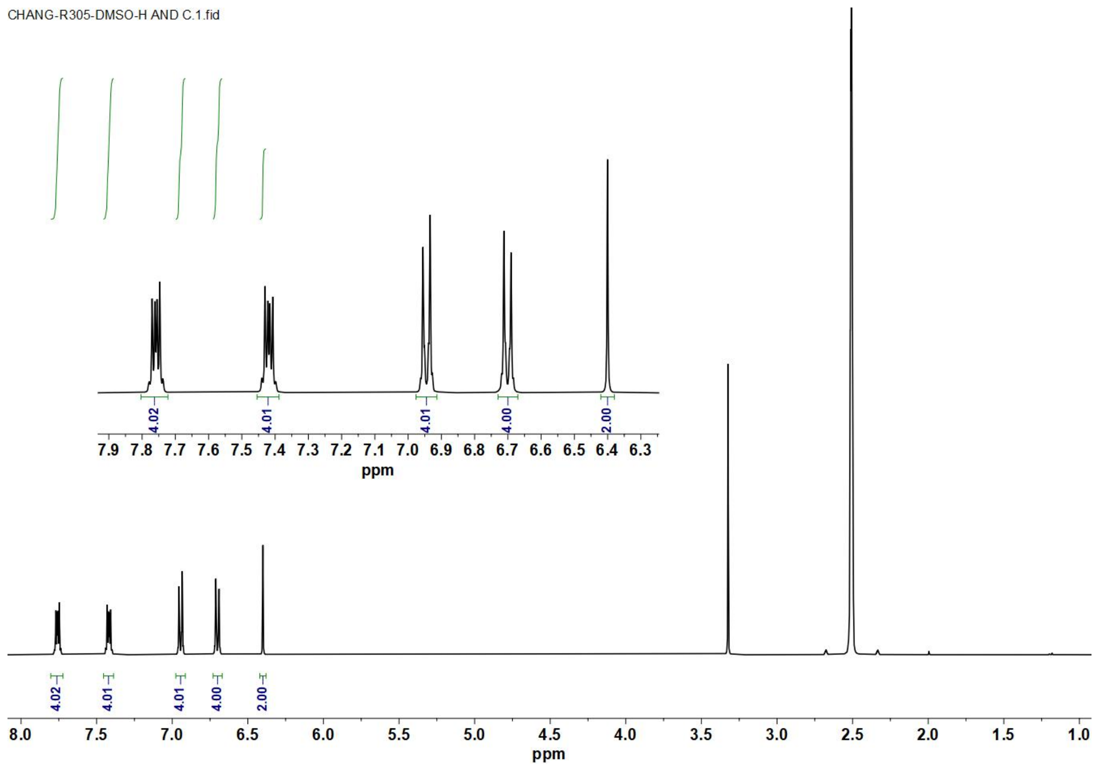


**Figure S49** $^{1}$H NMR of compound **S1** in DMSO-d6 (400 MHz, 298 K)

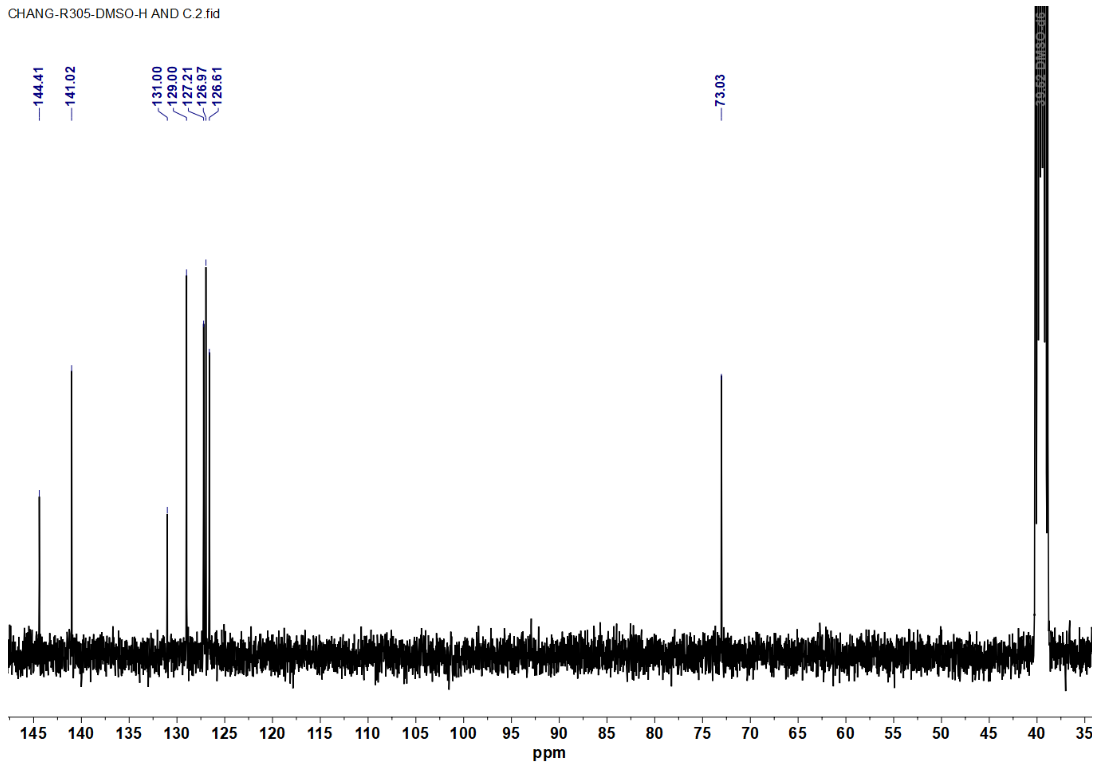


**Figure S50** $^{13}$C NMR of compound **S1** in DMSO-d6 (100 MHz, 298 K)

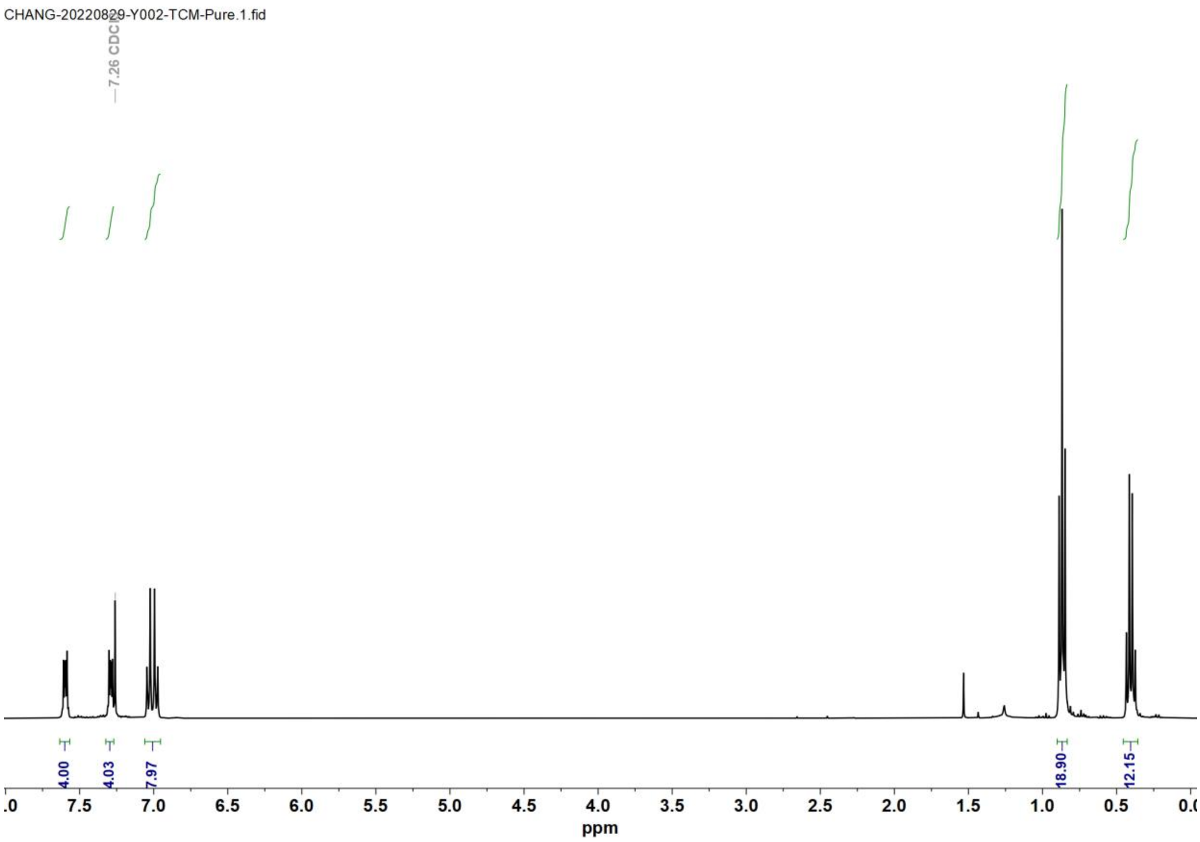


**Figure S51** $^{1}H$ NMR of compound **S2** in $CDCl_3$ (400 MHz, 298 K)

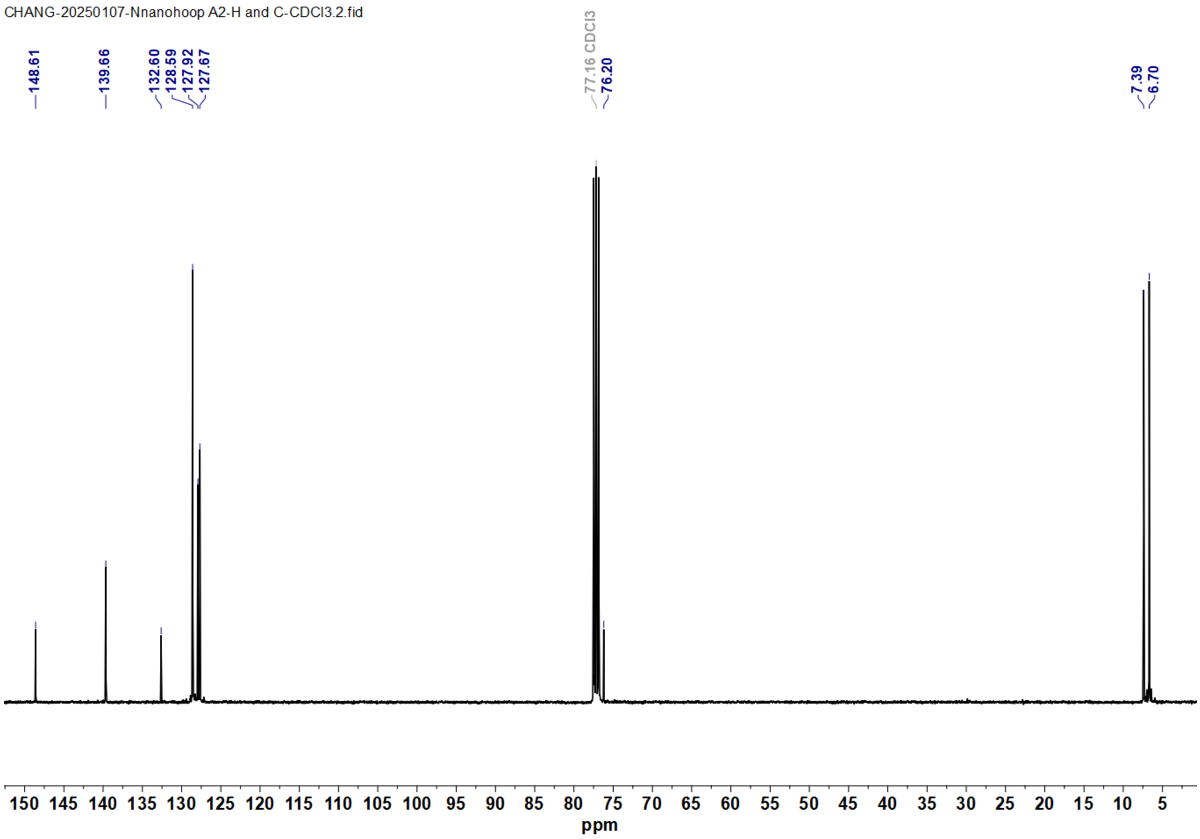


**Figure S52** $^{13}C$ NMR of compound **S2** in $CDCl_3$ (100 MHz, 298 K)

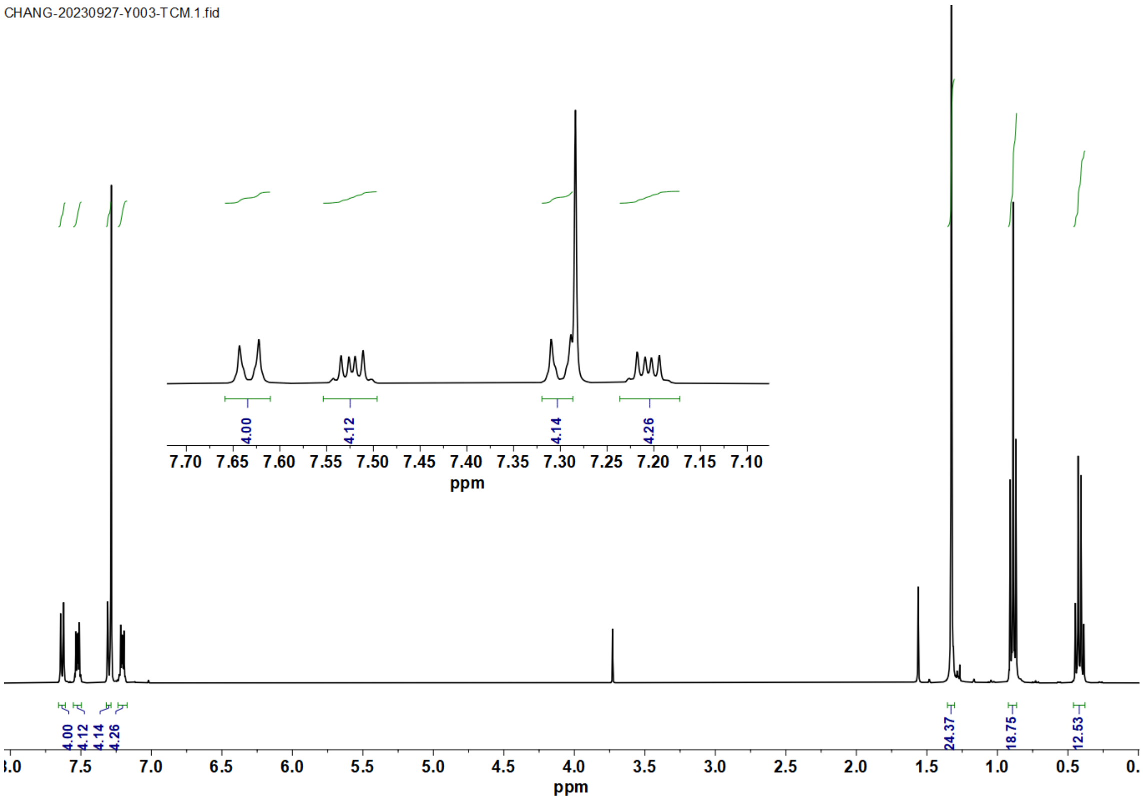


**Figure S53** $^{1}H$ NMR of compound **1** in $CDCl_3$ (400 MHz, 298 K)

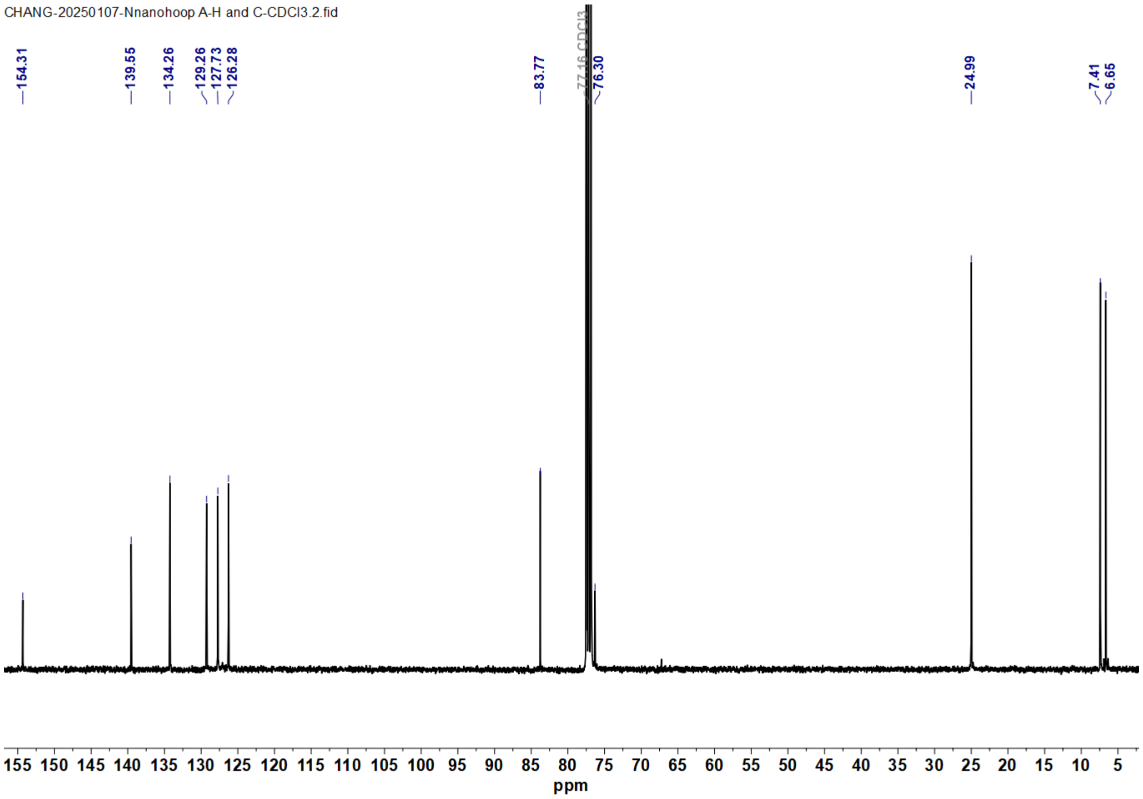


**Figure S54** $^{13}C$ NMR of compound **1** in $CDCl_3$ (100 MHz, 298 K)

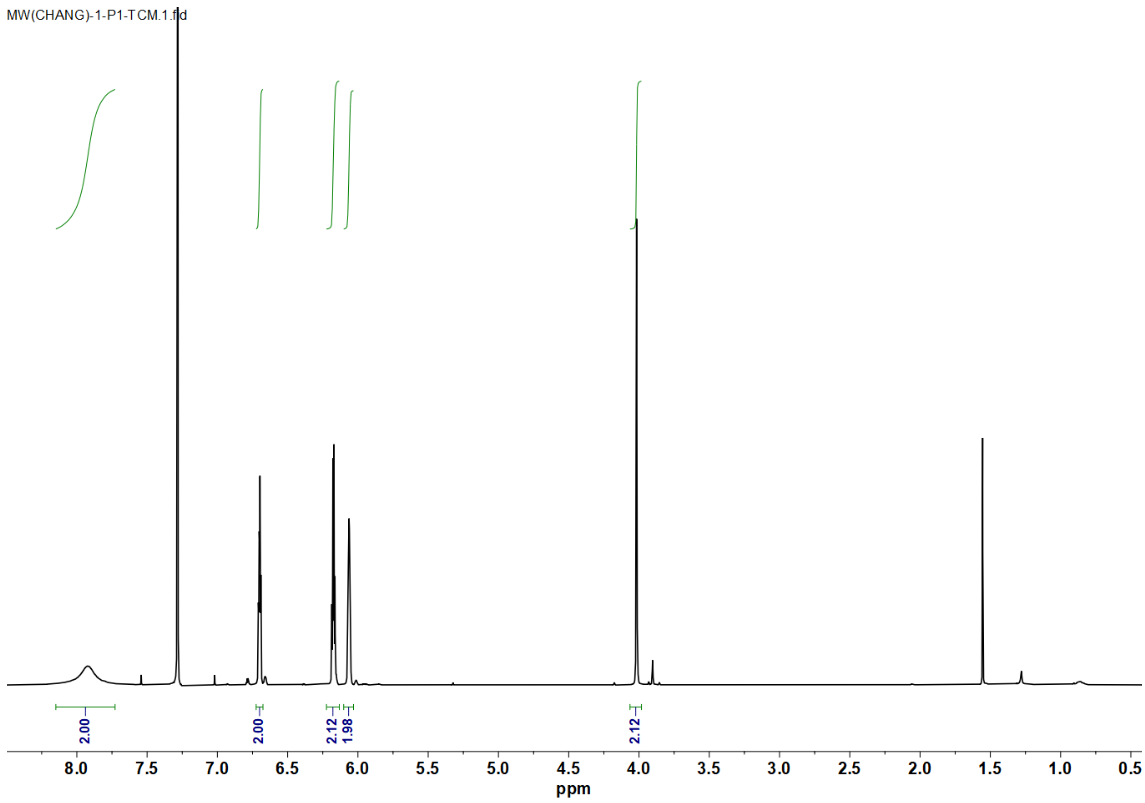


**Figure S55** $^1$H NMR of compound **S3** in $CDCl_3$ (400 MHz, 298 K)

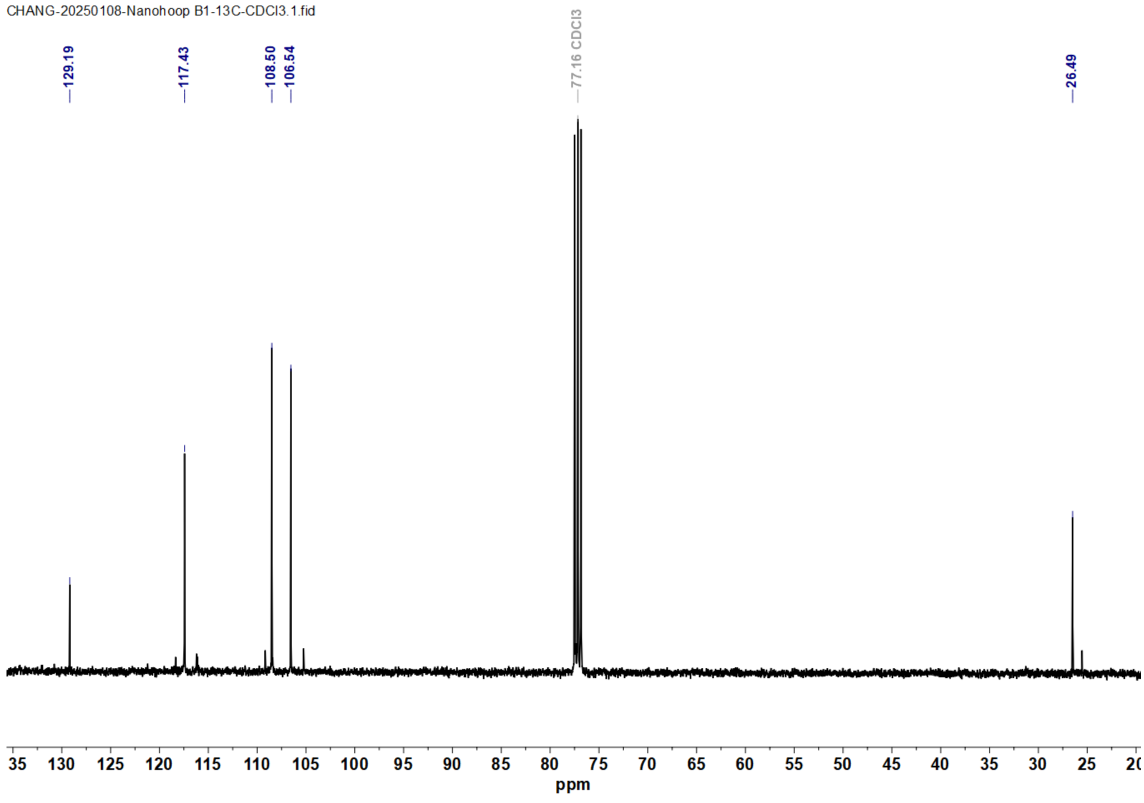


**Figure S56** $^{13}$C NMR of compound **S3** in $CDCl_3$ (100 MHz, 298 K)

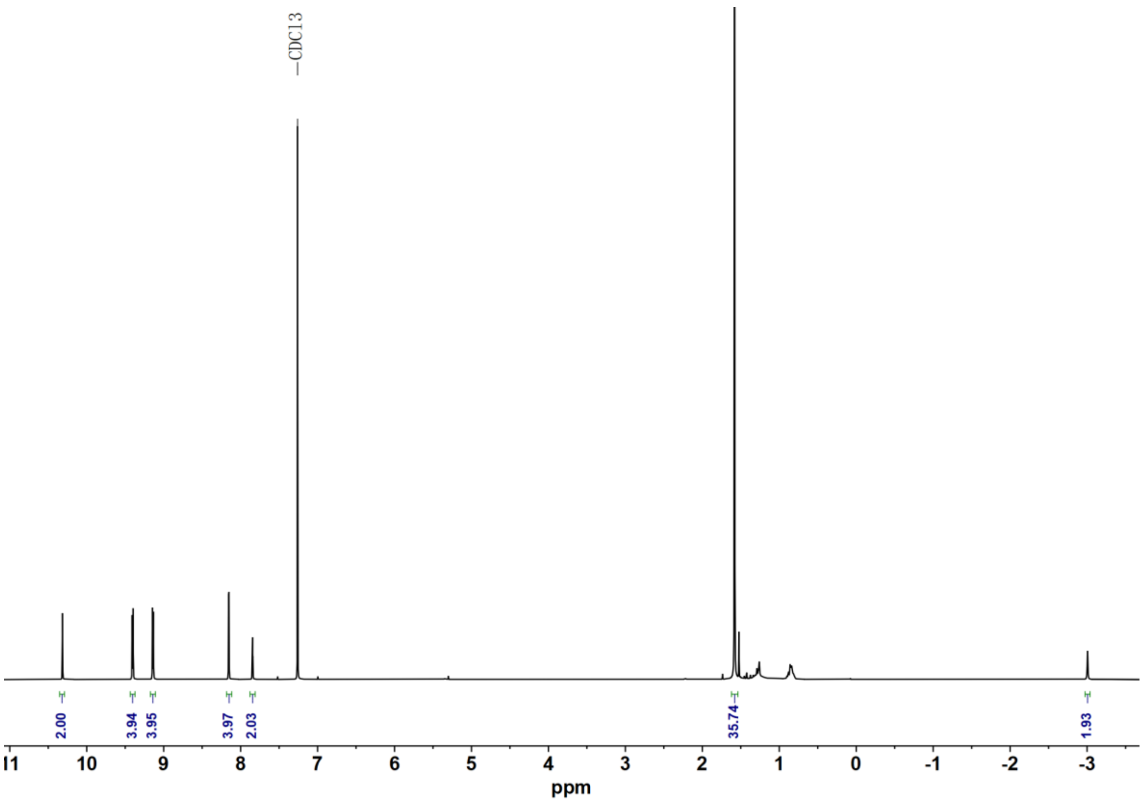


**Figure S57** $^{1}H$ NMR of compound **S4** in $CDCl_3$ (400 MHz, 298 K)

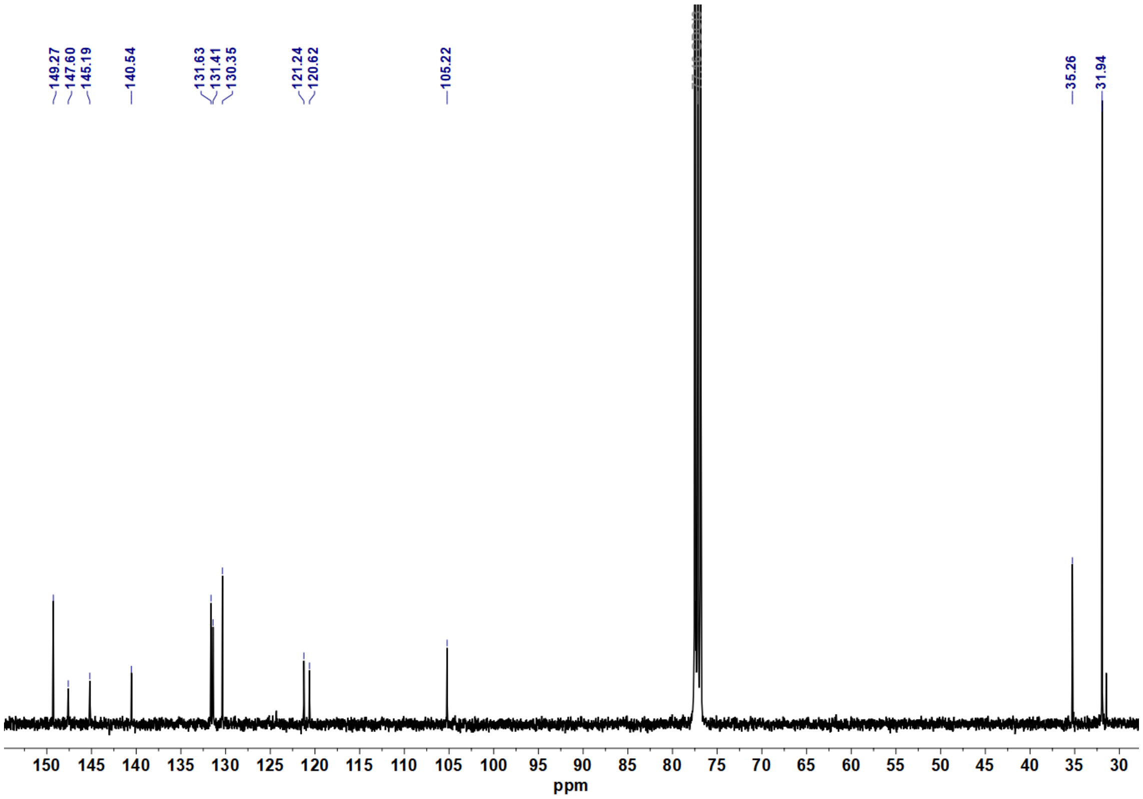


**Figure S58** $^{13}C$ NMR of compound **S4** in $CDCl_3$ (100 MHz, 298 K)

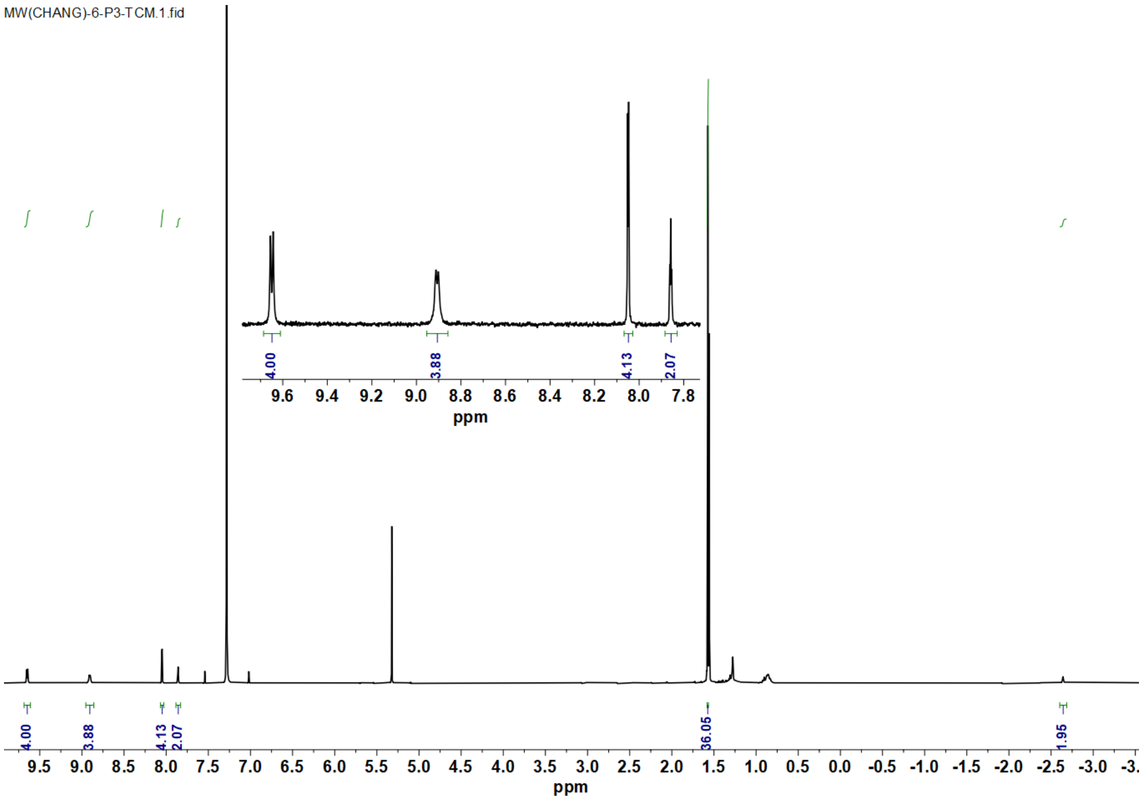


**Figure S59** $^1$H NMR of compound **2** in $CDCl_3$ (400 MHz, 298 K)

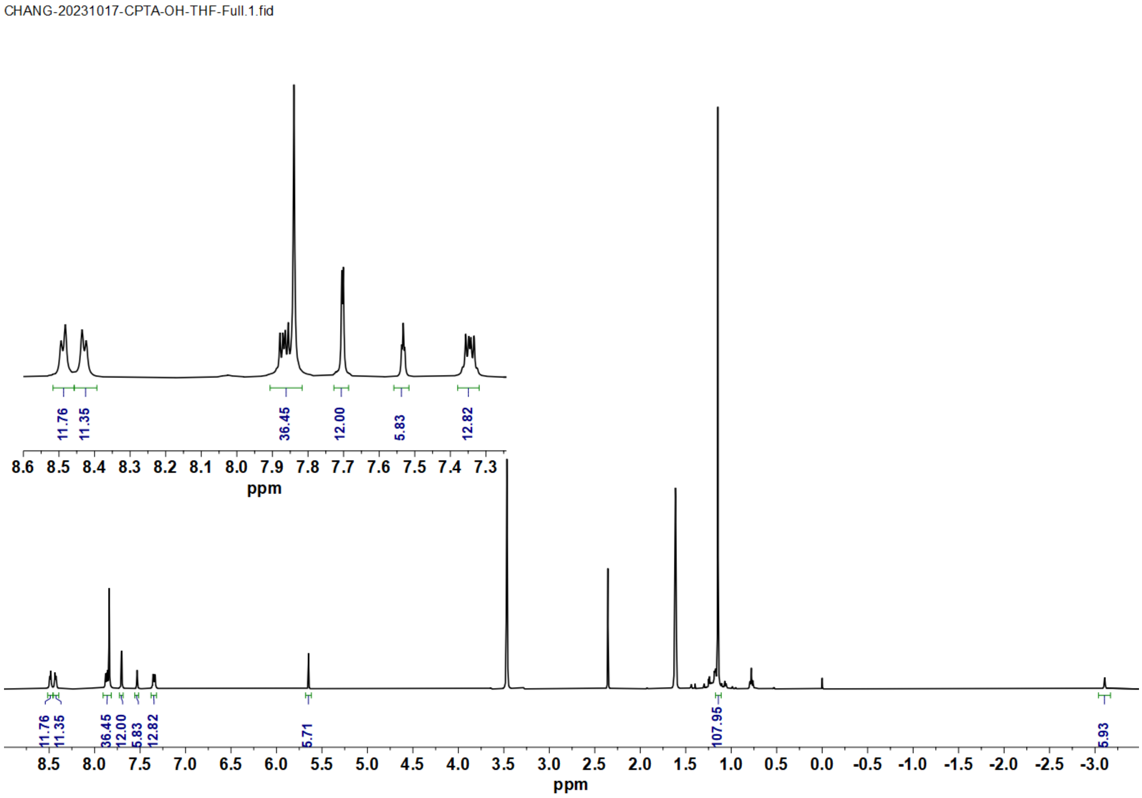


**Figure S60** $^1$H NMR of compound **CPTA-OH** in THF-$d_8$ (400 MHz, 298 K)

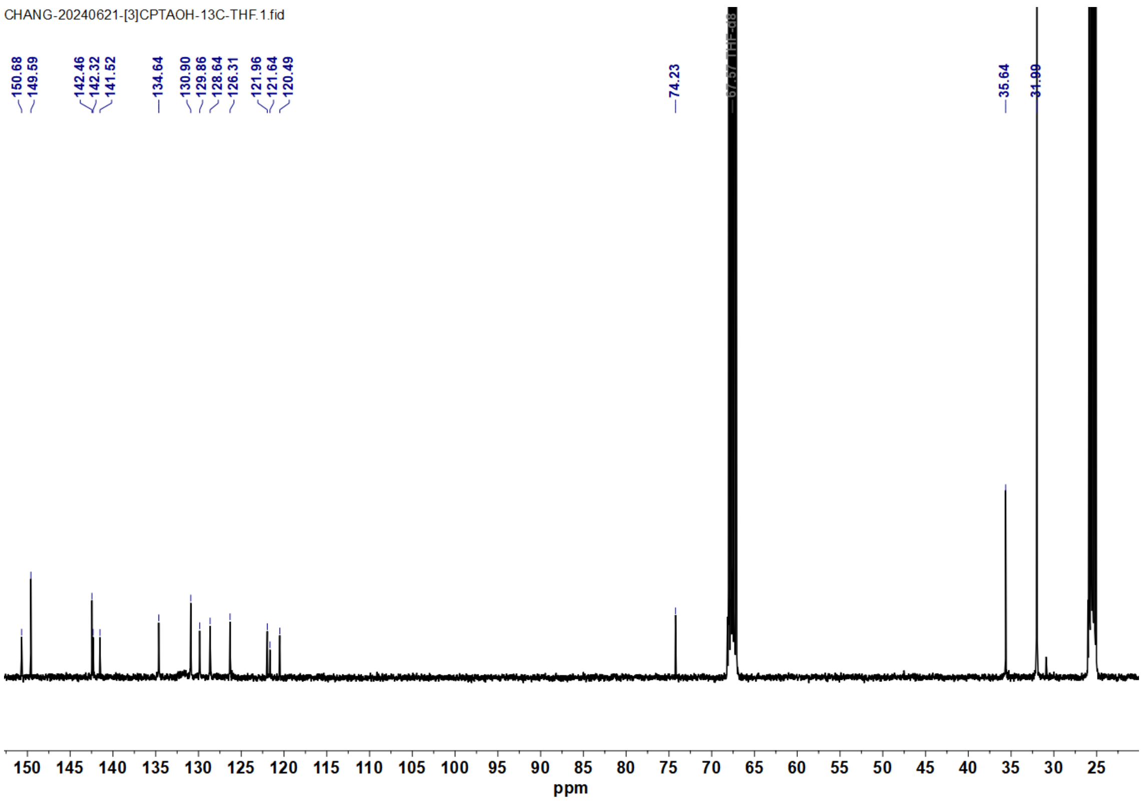


**Figure S61** $^{13}C$ NMR of compound **CPTA-OH** in THF-$d_8$ (100 MHz, 298 K)

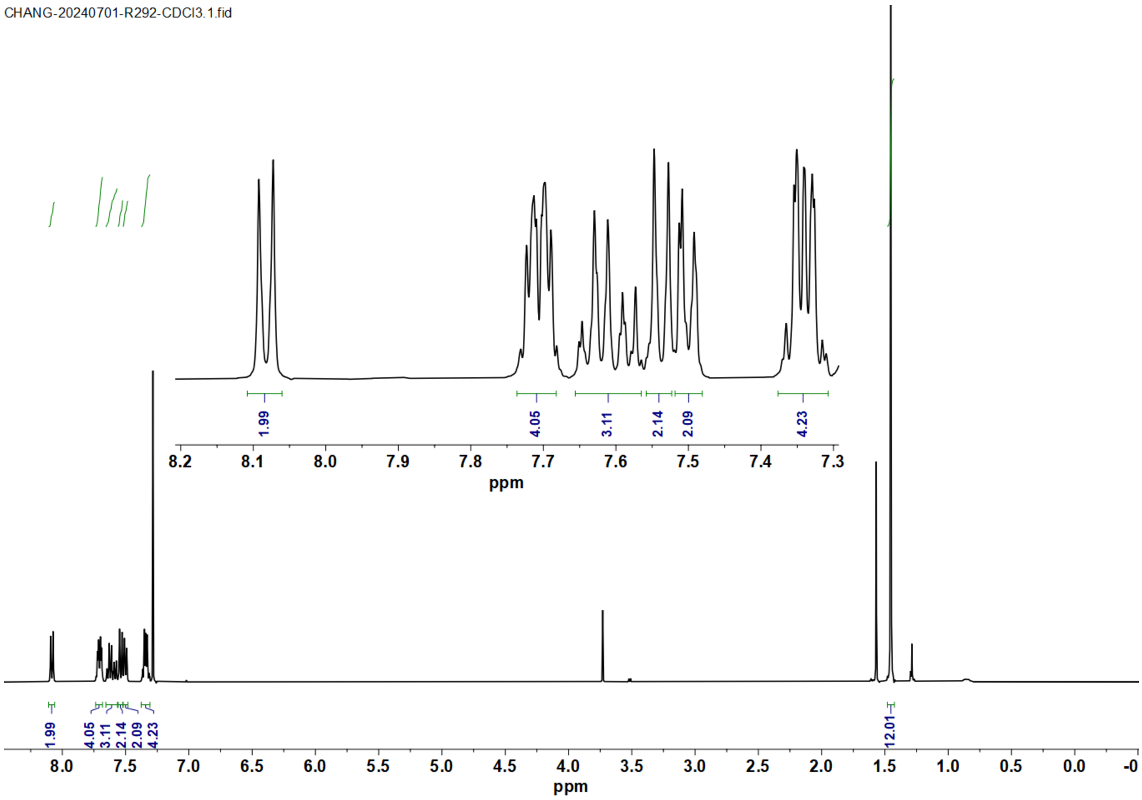


**Figure S62** $^{1}H$ NMR of compound **3** in $CDCl_3$ (400 MHz, 298 K)

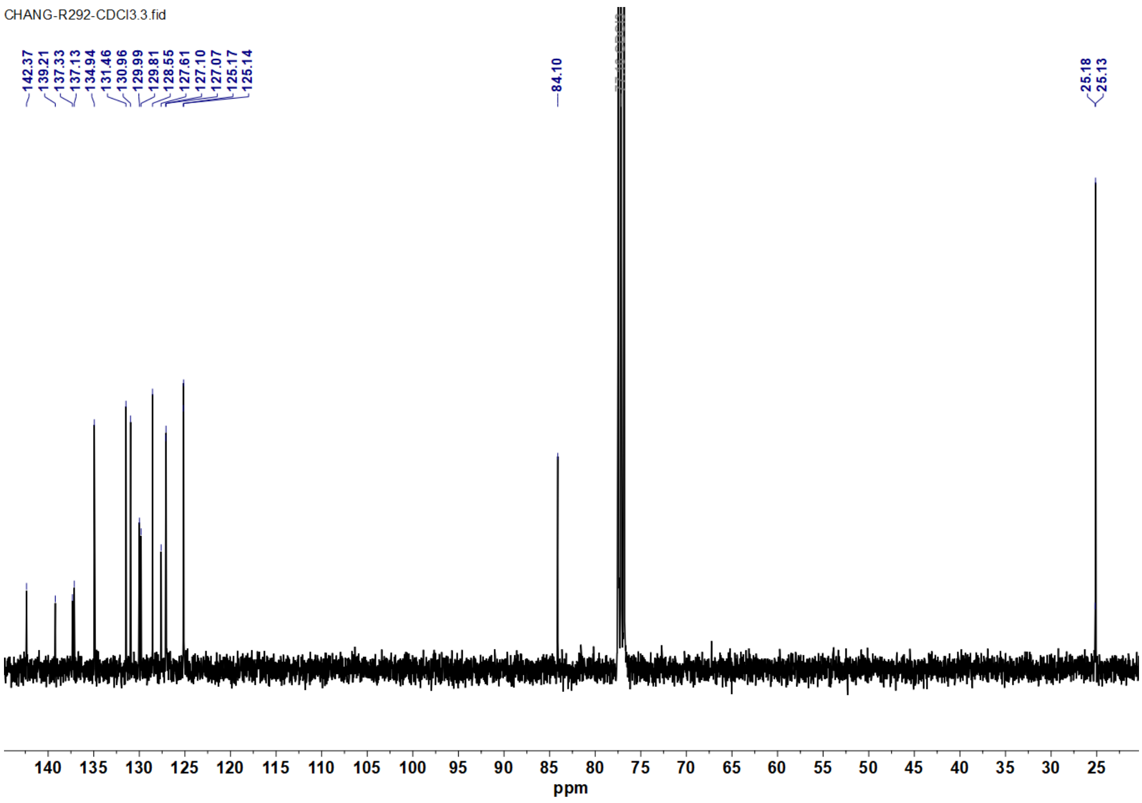


**Figure S63** $^{13}$C NMR of compound **3** in $CDCl_3$ (100 MHz, 298 K)

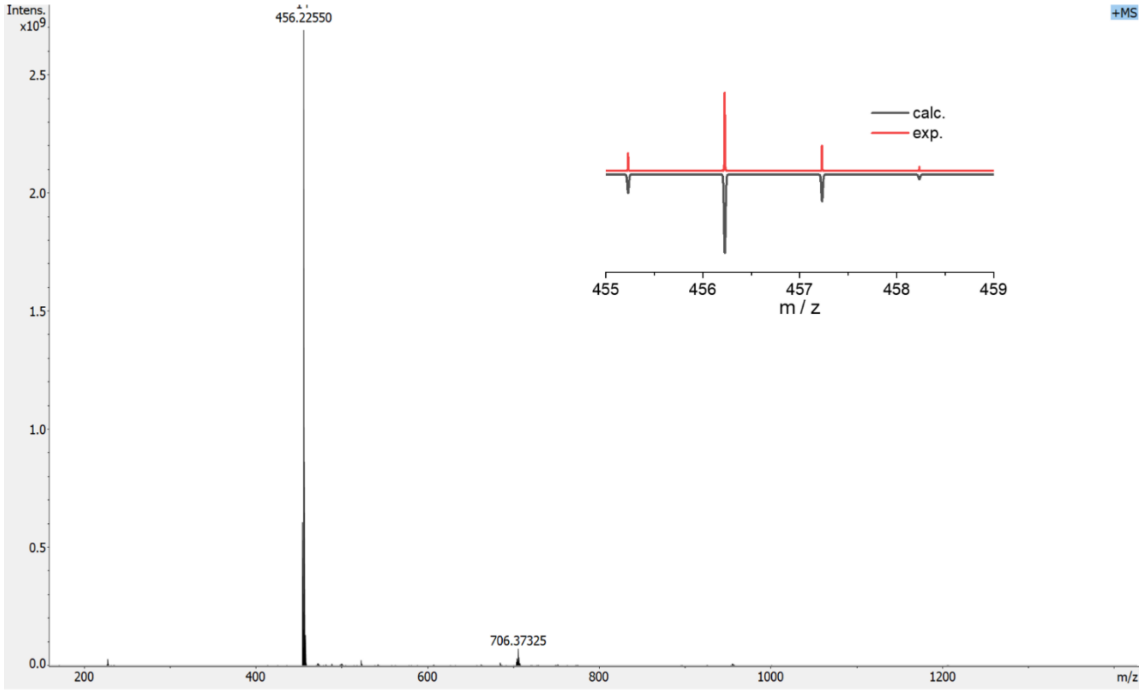


**Figure S64** HR-MS (MALDI-MS; positive mode; Matrix: DCTB) of compound **3**, including isotopic patterns.

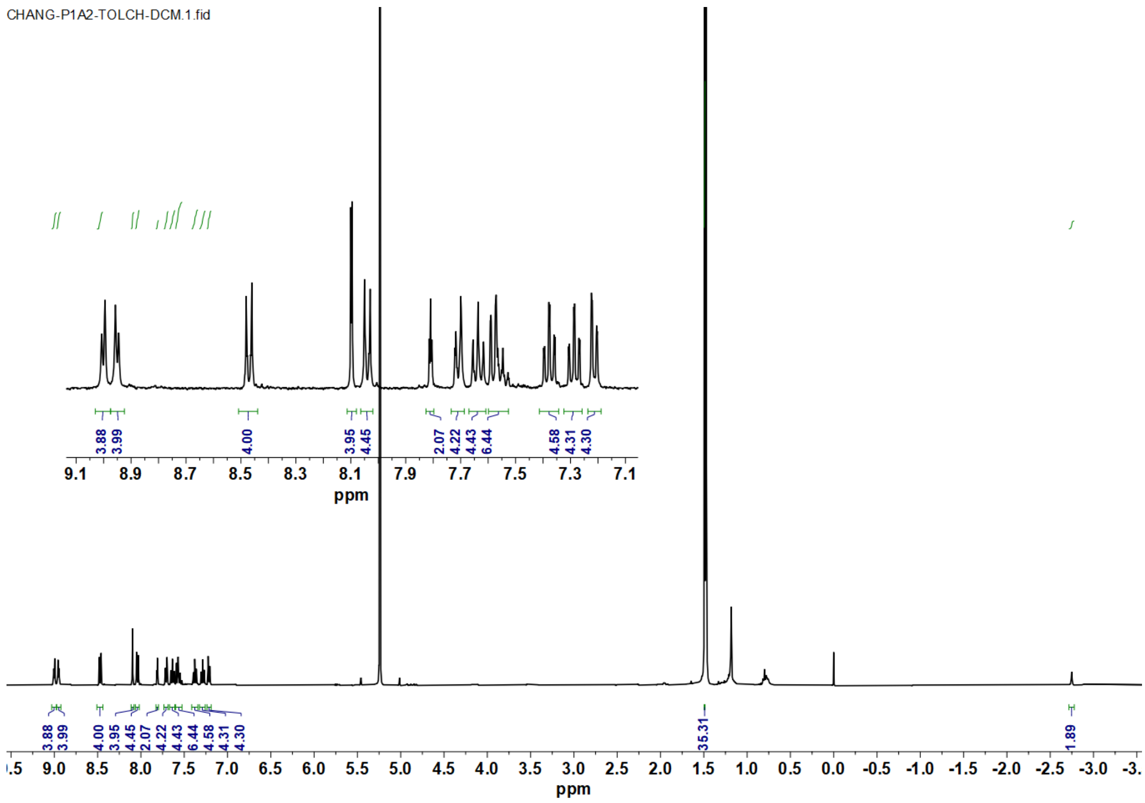


**Figure S65** $^{1}$H NMR of compound **P1A2** in $CD_2Cl_2$ (400 MHz, 298 K)

## 10. References


(1) Xu, Y.; Gsänger, S.; Minameyer, M. B.; Imaz, I.; Maspoch, D.; Shyshov, O.; Schwer, F.; Ribas, X.; Drewello, T.; Meyer, B.; von Delius, M. Highly Strained, Radially π-Conjugated Porphyrinylene Nanohoops. *J. Am. Chem. Soc.* **2019**, *141* (46), 18500–18507. https://doi.org/10.1021/jacs.9b08584.

(2) Jökel, J.; Schwer, F.; Von Delius, M.; Apfel, U.-P. A Dinuclear Porphyrin-Macrocycle as Efficient Catalyst for the Hydrogen Evolution Reaction. *Chem. Commun.* **2020**, *56* (91), 14179–14182. https://doi.org/10.1039/D0CC05229A.

(3) Juanhuix, J.; Gil-Ortiz, F.; Cuní, G.; Colldelram, C.; Nicolás, J.; Lidón, J.; Boter, E.; Ruget, C.; Ferrer, S.; Benach, J. Developments in Optics and Performance at BL13-XALOC, the Macromolecular Crystallography Beamline at the Alba Synchrotron. *J. Synchrotron Radiat.* **2014**, *21* (4), 679–689. https://doi.org/10.1107/S160057751400825X.

(4) Kabsch, W. Integration, Scaling, Space-Group Assignment and Post-Refinement. *Acta Crystallogr. Sect. D* **2010**, *66* (2), 133–144. https://doi.org/10.1107/S0907444909047374.

(5) Farrugia, L. J. WinGX and ORTEP for Windows: An Update. *J. Appl. Crystallogr.* **2012**, *45* (4), 849–854. https://doi.org/10.1107/S0021889812029111.

(6) Sheldrick, G. M. SHELXT – Integrated Space-Group and Crystal-Structure Determination. *Acta Crystallogr. Sect. Found. Adv.* **2015**, *71* (1), 3–8. https://doi.org/10.1107/S2053273314026370.

(7) Sheldrick, G. M. Crystal Structure Refinement with SHELXL. *Acta Crystallogr. Sect. C Struct. Chem.* **2015**, *71* (1), 3–8. https://doi.org/10.1107/S2053229614024218.

(8) Dolomanov, O. V.; Bourhis, L. J.; Gildea, R. J.; Howard, J. a. K.; Puschmann, H. OLEX2: A Complete Structure Solution, Refinement and Analysis Program. *J. Appl. Crystallogr.* **2009**, *42* (2), 339–341. https://doi.org/10.1107/S0021889808042726.

(9) Spek, A. L. Single-Crystal Structure Validation with the Program PLATON. *J. Appl. Crystallogr.* **2003**, *36* (1), 7–13. https://doi.org/10.1107/S0021889802022112.

(10) Blechschmidt, L.; Zedler, L.; Mengele, A. K.; Rau, S.; Dietzek-Ivanšić, B. Ligand Labilization Gates Intramolecular Electron Transfer in Molecular Photocatalyst. *Chem. Commun.* **2025**, *61* (12), 2508–2511. https://doi.org/10.1039/D4CC05844E.

(11) Müller, C.; Pascher, T.; Eriksson, A.; Chabera, P.; Uhlig, J. KiMoPack: A Python Package for Kinetic Modeling of the Chemical Mechanism. *J. Phys. Chem. A* **2022**, *126* (25), 4087–4099. https://doi.org/10.1021/acs.jpca.2c00907.

(12) Gouterman, M. 1 - Optical Spectra and Electronic Structure of Porphyrins and Related Rings. In *The Porphyrins*; Dolphin, D., Ed.; Academic Press, 1978; pp 1–165. https://doi.org/10.1016/B978-0-12-220103-5.50008-8.

(13) Rodriguez, J.; Kirmaier, C.; Holten, D. Optical Properties of Metalloporphyrin Excited States. *J. Am. Chem. Soc.* **1989**, *111* (17), 6500–6506. https://doi.org/10.1021/ja00199a004.

(14) Chirvony, V. S.; Négrerie, M.; Martin, J.-L.; Turpin, P.-Y. Picosecond Dynamics and Mechanisms of Photoexcited Cu(II)-5,10,15,20-Meso-Tetrakis(4-N-Methylpyridyl)Porphyrin Quenching by Oxygen-Containing Lewis-Base Solvents. *J. Phys. Chem. A* **2002**, *106* (24), 5760–5767. https://doi.org/10.1021/jp0134998.

(15) Jeong, D.; Kang, D.; Joo, T.; Kim, S. K. Femtosecond-Resolved Excited State Relaxation Dynamics of Copper (II) Tetraphenylporphyrin (CuTPP) After Soret Band Excitation. *Sci. Rep.* **2017**, *7* (1), 16865. https://doi.org/10.1038/s41598-017-17296-z.

(16) Ha-Thi, M.-H.; Shafizadeh, N.; Poisson, L.; Soep, B. An Efficient Indirect Mechanism for the Ultrafast

Intersystem Crossing in Copper Porphyrins. *J. Phys. Chem. A* **2013**, *117* (34), 8111–8118. https://doi.org/10.1021/jp4008015.
(17) Grollau, F.; Pothier, C.; Gaveau, M. A.; Briant, M.; Shafizadeh, N.; Soep, B. Action Spectroscopy of Spin Forbidden States in the Gas Phase: A Powerful Probe for Large Non-Luminescent Molecules. *J. Chem. Phys.* **2020**, *152* (14), 144306. https://doi.org/10.1063/5.0002890.
(18) McGarry, R. J.; Varvarezos, L.; Pryce, M. T.; Long, C. Excited-State Dynamics Leading Either to Triplet Formation or Coordinative Expansion Following Photolysis of Cu(II)-Porphyrins: A DFT, TD-DFT, Luminescence and Femtosecond Time-Resolved Absorbance Study. *Molecules* **2023**, *28* (17), 6310. https://doi.org/10.3390/molecules28176310.
(19) Gouterman, M.; Mathies, R. A.; Smith, B. E.; Caughey, W. S. Porphyrins. XIX. Tripdoublet and Quartet Luminescence in Cu and VO Complexes. *J. Chem. Phys.* **1970**, *52* (7), 3795–3802. https://doi.org/10.1063/1.1673560.
(20) Eastwood, D.; Gouterman, M. Porphyrins: XII. Luminescence of Copper Complexes at Liquid Nitrogen Temperature. *J. Mol. Spectrosc.* **1969**, *30* (1), 437–458. https://doi.org/10.1016/0022-2852(69)90276-8.
(21) Kruglik, S. G.; Apanasevich, P. A.; Chirvony, V. S.; Kvach, V. V.; Orlovich, V. A. Resonance Raman, CARS, and Picosecond Absorption Spectroscopy of Copper Porphyrins: The Evidence for the Exciplex Formation with Oxygen-Containing Solvent Molecules. *J. Phys. Chem.* **1995**, *99* (10), 2978–2995. https://doi.org/10.1021/j100010a006.
(22) Lorenc, M.; Ziolek, M.; Naskrecki, R.; Karolczak, J.; Kubicki, J.; Maciejewski, A. Artifacts in Femtosecond Transient Absorption Spectroscopy. *Appl. Phys. B* **2002**, *74* (1), 19–27. https://doi.org/10.1007/s003400100750.
(23) Kim, D.; Holten, D.; Gouterman, M. Evidence from Picosecond Transient Absorption and Kinetic Studies of Charge-Transfer States in Copper(II) Porphyrins. *J. Am. Chem. Soc.* **1984**, *106* (10), 2793–2798. https://doi.org/10.1021/ja00322a012.
(24) Smith, B. E.; Gouterman, M. Quartet Luminescence from Copper Porphyrins. *Chem. Phys. Lett.* **1968**, *2* (8), 517–519. https://doi.org/10.1016/0009-2614(63)80001-9.
(25) Yan, X.; Holten, D. Effects of Temperature and Solvent on Excited-State Deactivation of Copper(II) Octaethyl- and Tetraphenylporphyrin: Relaxation via a Ring-to-Metal Charge-Transfer Excited State. *J. Phys. Chem.* **1988**, *92* (21), 5982–5986. https://doi.org/10.1021/j100332a029.
(26) Herb, K.; Tschaggelar, R.; Denninger, G.; Jeschke, G. Double Resonance Calibration of g Factor Standards: Carbon Fibers as a High Precision Standard. *J. Magn. Reson.* **2018**, *289*, 100–106. https://doi.org/10.1016/j.jmr.2018.02.006.
(27) Lindgren, M.; Eaton, G. R.; Eaton, S. S.; Jonsson, B.-H.; Hammarström, P.; Svensson, M.; Carlsson, U. Electron Spin Echo Decay as a Probe of Aminoxyl Environment in Spin-Labeled Mutants of Human Carbonic Anhydrase II †. *J. Chem. Soc. Perkin Trans. 2* **1997**, No. 12, 2549–2554. https://doi.org/10.1039/a702470c.
(28) Soetbeer, J.; Hülsmann, M.; Godt, A.; Polyhach, Y.; Jeschke, G. Dynamical Decoupling of Nitroxides in *o*-Terphenyl: A Study of Temperature, Deuteration and Concentration Effects. *Phys. Chem. Chem. Phys.* **2018**, *20* (3), 1615–1628. https://doi.org/10.1039/C7CP07074H.
(29) Stoll, S.; Schweiger, A. EasySpin, a Comprehensive Software Package for Spectral Simulation and Analysis in EPR. *J. Magn. Reson.* **2006**, *178* (1), 42–55. https://doi.org/10.1016/j.jmr.2005.08.013.
(30) Asano-Someda, M.; Ichino, T.; Kaizu, Y. Triplet−Triplet Intramolecular Energy Transfer in a Covalently Linked Copper(II) Porphyrin−Free Base Porphyrin Hybrid Dimer: A Time-Resolved ESR Study. *J. Phys. Chem. A* **1997**, *101* (25), 4484–4490. https://doi.org/10.1021/jp962634r.

(31) Kirk, M. L.; Shultz, D. A.; Marri, A. R.; Hewitt, P.; van der Est, A. Single-Photon-Induced Electron Spin Polarization of Two Exchange-Coupled Stable Radicals. *J. Am. Chem. Soc.* **2022**, *144* (46), 21005–21009. https://doi.org/10.1021/jacs.2c09680.

(32) Atkins, P. W.; McLauchlan ,K.A.; and Percival, P. W. Electron Spin-Lattice Relaxation Times from the Decay of E.S.R. Emission Spectra. *Mol. Phys.* **1973**, *25* (2), 281–296. https://doi.org/10.1080/00268977300100271.

(33) Torrey, H. C. Transient Nutations in Nuclear Magnetic Resonance. *Phys. Rev.* **1949**, *76* (8), 1059–1068. https://doi.org/10.1103/PhysRev.76.1059.

(34) Furrer, R.; Fujara, P.; Lange, C.; Stehlik, D.; Vieth, H. M.; Vollmann, W. Transient ESR Nutation Signals in Excited Aromatic Triplet States. *Chem. Phys. Lett.* **1980**, *75* (2), 332–339. https://doi.org/10.1016/0009-2614(80)80526-4.

(35) Atkins, P. W.; Dobbs, A. J.; McLauchlan, K. A. Transient Nutations in Electron Spin Resonance. *Chem. Phys. Lett.* **1974**, *25* (1), 105–107. https://doi.org/10.1016/0009-2614(74)80343-X.

(36) Rozenshtein, V.; Berg, A.; Levanon, H.; Krueger, U.; Stehlik, D.; Kandrashkin, Y.; Van Der Est, A. Light-Induced Electron Spin Polarization in the Ground State of Water-Soluble Copper Porphyrins. *Isr. J. Chem.* **2003**, *43* (3–4), 373–381. https://doi.org/10.1560/FF1Q-02XD-Q4VC-DHX0.

(37) Fábregas Ibáñez, L.; Jeschke, G.; Stoll, S. DeerLab: A Comprehensive Software Package for Analyzing Dipolar Electron Paramagnetic Resonance Spectroscopy Data. *Magn. Reson.* **2020**, *1* (2), 209–224. https://doi.org/10.5194/mr-1-209-2020.

(38) Fábregas-Ibáñez, L.; Jeschke, G.; Stoll, S. Compactness Regularization in the Analysis of Dipolar EPR Spectroscopy Data. *J. Magn. Reson.* **2022**, *339*, 107218. https://doi.org/10.1016/j.jmr.2022.107218.

(39) Abdullin, D.; Rauh Corro, P.; Hett, T.; Schiemann, O. PDSFit: PDS Data Analysis in the Presence of Orientation Selectivity, g-Anisotropy, and Exchange Coupling. *Magn. Reson. Chem.* **2024**, *62* (1), 37–60. https://doi.org/10.1002/mrc.5415.

(40) Frisch, M. J.; Trucks, G. W.; Schlegel, H. B.; Scuseria, G. E.; Robb, M. A.; Cheeseman, J. R.; Scalmani, G.; Barone, V.; Petersson, G. A.; Nakatsuji, H.; Li, X.; Caricato, M.; Marenich, A. V.; Bloino, J.; Janesko, B. G.; Gomperts, R.; Mennucci, B.; Hratchian, H. P.; Ortiz, J. V.; Izmaylov, A. F.; Sonnenberg, J. L.; Williams; Ding, F.; Lipparini, F.; Egidi, F.; Goings, J.; Peng, B.; Petrone, A.; Henderson, T.; Ranasinghe, D.; Zakrzewski, V. G.; Gao, J.; Rega, N.; Zheng, G.; Liang, W.; Hada, M.; Ehara, M.; Toyota, K.; Fukuda, R.; Hasegawa, J.; Ishida, M.; Nakajima, T.; Honda, Y.; Kitao, O.; Nakai, H.; Vreven, T.; Throssell, K.; Montgomery Jr., J. A.; Peralta, J. E.; Ogliaro, F.; Bearpark, M. J.; Heyd, J. J.; Brothers, E. N.; Kudin, K. N.; Staroverov, V. N.; Keith, T. A.; Kobayashi, R.; Normand, J.; Raghavachari, K.; Rendell, A. P.; Burant, J. C.; Iyengar, S. S.; Tomasi, J.; Cossi, M.; Millam, J. M.; Klene, M.; Adamo, C.; Cammi, R.; Ochterski, J. W.; Martin, R. L.; Morokuma, K.; Farkas, O.; Foresman, J. B.; Fox, D. J. Gaussian 16 Rev. C.01, 2016.

(41) Ditchfield, R.; Hehre, W. J.; Pople, J. A. Self-Consistent Molecular-Orbital Methods. IX. An Extended Gaussian-Type Basis for Molecular-Orbital Studies of Organic Molecules. *J. Chem. Phys.* **1971**, *54*, 724–728. https://doi.org/10.1063/1.1674902.

(42) Hehre, W. J.; Ditchfield, R.; Pople, J. A. Self—Consistent Molecular Orbital Methods. XII. Further Extensions of Gaussian—Type Basis Sets for Use in Molecular Orbital Studies of Organic Molecules. *J. Chem. Phys.* **1972**, *56* (5), 2257–2261. https://doi.org/10.1063/1.1677527.

(43) Francl, M. M.; Pietro, W. J.; Hehre, W. J.; Binkley, J. S.; Gordon, M. S.; DeFrees, D. J.; Pople, J. A. Self-Consistent Molecular Orbital Methods. XXIII. A Polarization-Type Basis Set for Second-Row Elements. *J. Chem. Phys.* **1982**, *77* (7), 3654–3665. https://doi.org/10.1063/1.444267.

(44) Becke, A. D. Density-functional Thermochemistry. III. The Role of Exact Exchange. *J. Chem. Phys.* **1993**,

*98* (7), 5648–5652. https://doi.org/10.1063/1.464913.

(45) Grimme, S.; Antony, J.; Ehrlich, S.; Krieg, H. A Consistent and Accurate Ab Initio Parametrization of Density Functional Dispersion Correction (DFT-D) for the 94 Elements H-Pu. *J. Chem. Phys.* **2010**, *132* (15), 154104. https://doi.org/10.1063/1.3382344.

(46) Grimme, S.; Ehrlich, S.; Goerigk, L. Effect of the Damping Function in Dispersion Corrected Density Functional Theory. *J. Comput. Chem.* **2011**, *32* (7), 1456–1465. https://doi.org/10.1002/jcc.21759.
